# Conserved Signatures in Protein Sequences Reliably Demarcate Different Clades of Rodents/Glires Species and Consolidate Their Evolutionary Relationships

**DOI:** 10.3390/genes13020288

**Published:** 2022-02-01

**Authors:** Radhey S. Gupta, Carson Suggett

**Affiliations:** Department of Biochemistry and Biomedical Sciences McMaster University, Hamilton, ON L8N 3Z5, Canada; suggettcarson@gmail.com

**Keywords:** genome sequences, molecular markers (synapomorphies), phylogenetic trees, conserved signature indels, evolutionary relationships among Glires, Rodentia and Lagomorpha orders, Castorimorpha, Hystricomorpha, Myomorpha and Sciuromorpha suborders, *Muridae* superfamily

## Abstract

The grandorder Glires, consisting of the orders Rodentia and Lagomorpha, encompasses a significant portion of the extant mammalian species including Rat, Mouse, Squirrel, Guinea pig and Beaver. Glires species play an important role in the ecosystem and provide valuable animal models for genetic studies and animal testing. Thus, it is important to reliably determine their evolutionary relationships and identify molecular characteristics that are specific for different species groups within the Glires. In this work, we have constructed a phylogenetic tree for >30 genome sequenced Glires species based on concatenated sequences of 25 conserved proteins. In this tree, members of different orders, suborders, and families within Glires formed strongly supported clades, and their interrelationships were also generally reliably resolved. In parallel, we conducted comparative analyses on more than 1500 protein sequences from Glires species to identify highly conserved molecular markers. These markers were comprised of conserved signature indels (CSIs) in proteins, which are specific for different Rodentia/Glires clades. Of the 41 novel CSIs identified in this work, some are specific for the entire Glires, Rodentia, or Lagomorpha clades, whereas many others reliably demarcate different family/suborder level clades of Rodentia (viz. Myomorpha, Castorimorpha, Sciuromorpha, Hystricomorpha, and Muroidea). Additionally, some of the CSIs also provide information regarding the interrelationships among Rodentia subgroups. Our analysis has also identified one CSI that is commonly shared by the Glires and Scandentia species (tree shrew), however, its evolutionary significance is unclear. Several of the identifed rodents-specific CSIs are present in conserved disease-related proteins. Thus, they provide novel molecular markers for genetic and biochemical studies on the functions of these proteins.

## 1. Introduction

The grandorder Glires is made up of two important orders, Rodentia and Lagomorpha containing several household animals [1,2,3]. Of these, the order Rodentia, which contains >2500 species of rodents, represents approximately 40% of extant mammals [2,3]. Members of this order were initially defined by teeth and masseter muscles that have mastered the ability to break down hard organic matter [2,3]. Rodent species are found on all continents except Antarctica, and they differ considerably in shapes and sizes [2,3]. Since rodent/glires species comprise a large and important group of mammals it is of much importance to understand the evolutionary relationships and classification of these species. This knowledge should be of great value as rodent species are widely used as animal models for studying different diseases and in evaluating the effects of different chemicals and therapeutics [4,5,6]. 

The early attempts to sort Rodentia were based on morphological characteristics [2,3], but these classifications were full of convergent and parallel evolution [1,7]. However, in the past two decades the application of molecular sequence-based approaches to the field of rodent phylogeny, has provided much clarity in terms of understanding their evolutionary relationships [1,8,9,10,11,12,13,14]. Based on phylogenetic trees constructed using different gene and protein sequences, and insertion patterns of transposable elements [8], the order Rodentia forms a monophyletic lineage, and it is a sister group of the order Lagomorpha. Together these two orders comprise the grandorder Glires [1,13,15,16]. The Glires in turn forms a sister group of the grandorder Euarchonta (consisting of the orders Scandentia, Dermoptera, and Primates, and together these two grandorders form the superorder Euarchontoglires [1,7,13,15,16]. Although there is no one widely accepted branching pattern for the evolution of Rodentia, most researchers agree on the presence of four main clades within Rodentia viz., Myomorpha, Sciuromorpha, Hystricomorpha, and Castorimorpha [1,7,8,9,10,11,12,13,14,17]. Of these suborders, Myomorpha, the mouse-related clade constitutes the largest suborder, and some classification/investigators also recognize its close association with another suborder Anomaluromorpha [2,7,17]. The other suborders within Rodentia include Sciuromorpha, or the squirrel-related clade and Hystricomorpha, or the guinea-pig related clade [2,7,17]. In addition, the current classification also recognizes Castorimorpha as a separate suborder, but many studies have placed it within Myomorpha [1,2,7,12,13,15,16]. The order Lagomorpha contains limited number of species such as rabbits and hares [1,16,18,19,20]. 

Although phylogenetic studies in recent years have considerably advanced our understanding of the evolutionary relationships among rodent species, the trees based on different gene/protein sequences often differ in terms of branching order of the main lineages of Rodentia [1,8,9,10,11,12,13,14]. Thus, based on phylogenetic approaches, the branching order and interrelationships among the major clades or suborders of Rodentia are not clearly understood [2,13]. Besides the phylogenetic studies, Churakov et al. [8] have used the presence/absence of retroposons to investigate the evolutionary relationships among the Rodentia clades. Their work identified 65 retroposons, which were shared by specific groups/clades of rodent species, providing important information regarding their evolutionary relationships. This study identified several retroposons which were specific for all rodents and provided evidence for the squirrel related clade (i.e., Sciuromorpha) to be the most basal suborder with Rodentia [8]. However, the interrelationships among some of the suborders of Rodentia (viz. Myomorpha and Castorimorpha) was not resolved and some retroposons yielded conflicting results [8]. Despite the important advancements in genomics, different main subgroups within Rodentia (i. e. suborders and families) are currently mainly distinguished from each other based on their branching in phylogenetic trees and some morphological traits [2,8,13]. Besides the retroposons, there are very reliable molecular/biochemical characteristics known which can clearly distinguish different suborders of rodents from each other [8].

Genome sequences are now available for multiple species from different suborders of rodents, as well as representative Lagomorpha species, and species from other groups/orders of Euarchontoglires. These genomes provide a valuable resource for examining the evolutionary relationships among these species by construction of phylogenetic trees, based on large datasets of genes/proteins sequences. However, the construction of whole genome trees for mammalian species presents several practical problems. One of the main difficulties in this regard is that mammalian species contain multiple isoforms/homologs for numerous proteins, which are not readily distinguished from each other. This greatly increases the chances that sequence alignments for many proteins will be made up of paralogs and the constructed tree could be misleading. To avoid this problem, we have constructed a phylogenetic tree based on concatenated sequences of 25 conserved proteins that are either present in a single copy in all genomes, or where paralogs can be reliably distinguished. Based on earlier studies, the resolving power of a tree based on 20 or more average size proteins is comparable to that based on whole genomes [21,22], hence this tree should be reliable. In addition to the construction of more robust phylogenetic trees, genomes provide a unique resource for discovery of novel molecular markers that are uniquely shared by different main groups/suborders within the Glires. These markers should provide reliable means for the demarcation of different Rodents/Glires clades and for the understanding of their interrelationships. One important class of molecular markers whose discovery has been facilitated by analyses of genome sequence, is comprised of conserved signature indels (insertions/deletions) (CSIs) in gene/protein sequences that are uniquely shared by an evolutionarily related group of species [23,24,25,26,27]. The CSIs within conserved regions of genes/proteins result from rare genetic changes. Even a 1 aa insertion or deletion within a protein coding sequence involves a 3-base pair in-frame insertion or deletion in the gene and thus constitutes a rare event [23,27,28,29]. In view of the discrete nature of these genetic changes which are located at specific positions in protein sequences, their presence or absence in different lineages is generally not affected by various factors that can confound inferences from phylogenetic trees [29,30,31,32]. Hence, when a CSI of a definite length is present at a specific position, in a protein present within a phylogenetically coherent group of organisms, its most parsimonious explanation is that the genetic change giving rise to this CSI occurred in a common ancestor of the group and then vertically inherited by the other group members [23,28,29,30]. Furthermore, based upon the presence or absence of a CSI in outgroup species, it is possible to infer whether a given CSI represents an insert or a deletion. Thus, based on this information a rooted relationship can be developed independently of phylogenetic trees [12,23,25,33,34]. Due to the above characteristics, the CSIs in protein sequences have proven very useful in clarifying several important evolutionary relationships, which had proven difficult to establish by other means [12,23,24,25,33,34,35]. Although, while most of the studied CSIs constitute synapomorphies, in some instances, when they are commonly shared by phylogenetically unrelated group of organisms, they can result from homoplasy or lateral gene transfers [27,30,36]. 

In the present study, we have used genome sequences for Glires as well as other representatives of Euarchontoglires species, to construct a robust phylogenetic tree based on concatenated sequences for 25 conserved proteins. In this tree, all the major groups/clades within the Glires, as well as different suborders of Rodentia viz. Castorimorpha, Hystricomorpha, Myomorpha, and Sciuromorpha formed strongly supported clades. Furthermore, in this rooted tree, most of the internal branches and clades within the Glires were also well-resolved with high degree of bootstrap support. Besides the construction of a phylogenetic tree, an important aspect of this work focused on comparative genomic studies on >1500 proteins from the Glires and other Euarchontoglires species. These comparative genomic studies have the aim of identifying CSIs that are specific for either the Rodentia and Lagomorpha orders or different main clades and suborders within the Glires species. This resulted in the identification of 41 CSIs in diverse proteins that are specific for different orders and suborders of Glires, in addition to providing information regarding their evolutionary relationships. The molecular markers identified include some that are specific for the orders Rodentia and Lagomorpha as well as multiple other signatures demarcating the suborders Castorimorpha, Myomorpha, Sciuromorpha, and Hystricomorpha [37,38,39,40,41]. Due to their predicted functional importance, the molecular markers described here, which are specific for different groups of rodents, also provide novel markers for genetic and biochemical studies on rodent species [12,18,42]. 

## 2. Materials and Methods

### 2.1. Construction of Phylogenetic Tree 

A phylogenetic tree was constructed for 30 glires and related species, whose annotated genome sequences were available in the NCBI database (https://www.ncbi.nlm.nih.gov/genome/) as of 1 April 2021. In addition to the glires species, our dataset also included sequences for five different Euarchontoglires species, which served as outgroups for rooting of the tree. The tree was constructed based on concatenated sequences for 25 conserved proteins, which based on our analysis are present in a single copy within the glires and related species. Information for the proteins that were used for tree construction is provided in Table S1. Multiple sequence alignments for each of these proteins were created using the ClustalX 2.1 program [43]. After arranging these sequences in the same order using an internally developed script, the alignment files were concatenated into a single large file. Sequence regions showing poor sequence conservation were removed from this file using the Gblocks_0.91b program [44]. The resulting sequence alignment which contained a total of 20106 aligned characters was used for phylogenetic analyses. A maximum likelihood trees based on 100 bootstrap replicates of this alignment was constructed using MEGA X software [45] employing the Whelan and Goldman [46] model of protein sequence evolution and Jones–Taylor–Thornton [47] substitution models, respectively, as described in our earlier work [34,48]. During analysis, all positions with less than 95% site coverage were eliminated. The tree with the highest log likelihood (−250500.22) is shown. The percentage of trees in which the associated taxa clustered together is shown next to the branches. 

### 2.2. Identification of Conserved Signature Indels (CSIs)

The identification of CSIs was carried out as described in earlier work [25,27,34]. BLASTp (Basic Local Alignment Search Tool, p refers to protein) searches were carried out on >1400 protein sequences from rat genome (*Rattus rattus*) (accession numbers range XP_032740061.- XP_032741506.1) that were >100 amino acids in length, against the NCBI non-redundant database. Multiple sequence alignments of these proteins were constructed using ClustalX 2.1 [43] on 10–15 protein homologs covering different glires species and 8–10 homologs from other mammalian species. In addition to these sequence alignments, >500 other protein sequence alignments constructed in our earlier work [26] were also utilized. The alignments were visually inspected for insertions or deletions of fixed lengths which were flanked on both sides by at least 4–5 conserved amino acids in the adjacent 40–50 amino acids and appeared to be exclusive to some or all glires species. The indels which were not flanked by conserved regions were not investigated as they do not provide reliable molecular markers [27,29,49,50]. Additionally, in the present work, we have primarily looked for those CSIs, which are specific for most of the species from different observed clades of Glires. Hence, the CSIs that were present in 1–2 isolated rodent species were generally not further studied. Query sequences encompassing the indel and its flanking 50–100 amino acids were subjected to another BLASTp search against the nr database. The top 250 hits from these blast searches were examined to determine the group specificities of the CSIs. In addition, specific BLASTp searches were also carried out for Euarchontoglires to detect the presence or absence of the CSIs in different Euarchontoglires species. Signature files for all CSIs were created using SIG_CREATE and SIG_STYLE programs described in our earlier work [27,34] that are available on the GLEANS (Gupta Lab Evolutionary Analysis Software) (Gleans.net) server. The CSIs reported here, unless otherwise indicated, are specific for all members of the indicated groups, whose homologs were detected by BLASTp searches. For larger clades within the Glires (viz. Rodentia, Glires), sequence information is shown for other Euarchontoglires as well as several other mammalian/vertebrate species. However, for the smaller clades within the glires, sequence information is shown only for the other glires and Euarchontoglires species and was used for determining the specificities of the identified CSIs. The dashes (–) in all sequence alignment figures denote identity with the amino acid found on the top line. More detailed sequence information for the outgroup species is presented in supplemental figures.

## 3. Results

### 3.1. Phylogenetic Analysis of Rodentia

**Figure 1:**
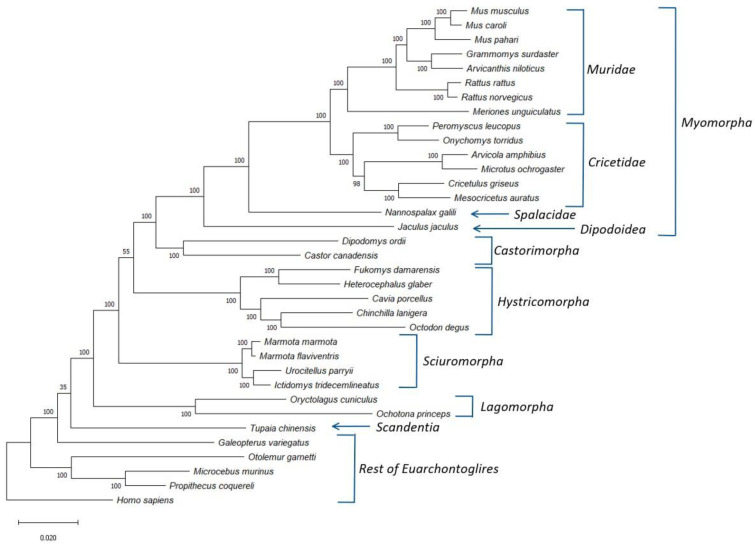
A maximum likelihood distance tree for the Glires species based on concatenated sequences for 25 conserved proteins. The bootstrap score for each branch point is shown at the nodes and numbers on the bar at the bottom indicates the number of changes per position. Major clades within the Glires/Rodentia are labelled and the tree was rooted using the *Homo sapiens* sequence.

Using protein sequences from the genomes of more than 30 glires species, we have identified 25 proteins (Table S1) that are present in different glires species and are found in a single copy within the studied genomes. We have used the concatenated sequence alignment of these proteins to construct a boot-strapped maximum-likelihood (ML) tree for the glires species. The constructed tree also contained information for some other Euarchontoglires species, which were used for the rooting of the tree. The resultant boot-strapped tree rooted using the sequence for *Homo sapiens* is shown in Figure 1**.** The tree shown in Figure 1 displays high degree of resolution and except for two branches, all other major nodes are supported by 100% bootstrap scores indicating that the observed relationships are reliable. Earlier studies have shown that the resolving power of a tree based on concatenated sequences for 20 or so average size proteins is comparable to that based on whole genomes [21,22]. At the highest level, the species from the orders Rodentia and Lagomorpha both form monophyletic clades, which are separated from each other by a long branch. Further, a combined clade consisting of these two orders, representing the grandorder Glires, is also strongly supported (100% bootstrap score). The observed monophyly of the Rodentia and Lagomorpha and their sister relationship also concurs with earlier studies [1,8,20]. The tree also shows Glires in a weak sister relationship with the sole species (*Tupaia chinensis*) from the order Scandentia. A similar branching of Scandentia has also been observed in earlier studies [20,42]. However, the node supporting a sister group relationship between these two groups has a bootstrap score of only 35, indicating that this relationship is not reliable. 

The tree shown in Figure 1 also provides important insights into the interrelationships among different suborders of Rodentia. First, all four main suborders of Rodentia for which sequences information was available viz., Castorimorpha, Hystricomorpha, Myomorpha, and Sciuromorpha, formed strongly supported monophyletic clades in the tree. Of these suborders, Sciuromorpha was found to diverge early in comparison to the other suborders. A sister group relationship between the suborders Castorimorpha and Myomorpha, observed in earlier studies [9,12], is also strongly supported by the tree. Furthermore, although the tree shown in Figure 1, places the suborder Hystricomorpha, as the first divergence of the clade consisting of the suborders Castorimorpha and Myomorpha, the statistical support for this relationship is relatively weak (bootstrap score 55). However, several earlier studies also support a sister group relationship between Hystricomorpha and Myomorpha, with Sciuromorpha branching off first [1,8,9,11]. In addition to the clear distinction of different Rodentia suborders, the four main groups/families within the suborder Myomorpha viz., Cricetidae, Dipodoidea, Muridae, and Spalacidae, also form well-defined monophyletic lineages in the tree. Of these four groups, Dipodoidea and Spalacidae are each represented by a single species in this tree, and both branched deeply in comparison to the other Myomorpha families. 

### 3.2. Identification of Molecular Markers Specific for Different Main Groups within the Glires

Although the phylogenetic tree shown in Figure 1 provides important insights regarding the overall evolutionary relationships among the Glires, several branches in it particularly those showing an association of the Scandentia with Glires and a sister relationship of the Hystricomorpha to the clade containing Myomorpha and Castorimorpha were not resolved. These relationships were also not resolved by earlier phylogenetic studies [1,8,9,11]. However, phylogenetic trees are dynamic constructs and branching of species in them is affected by large numbers of variables including but not limited to, the species that are present in the dataset, sequence alignment of the genes/proteins, difference in evolutionary rates among species, evolutionary model used for tree construction, etc., [30,51]. Additionally, an important limitation of the phylogenetic trees is that they do not provide any information regarding what biochemical, molecular, or any other characteristic is commonly shared by the species from different observed clades, and the means to identify them [29,30,49].Thus, it is important to use other approaches which can confirm the inferences from phylogenetic trees and simultaneously afford novel molecular characteristics that are specific for different groups of species [8,25,34,52]. As noted in the introduction, the CSIs in protein sequences that are uniquely shared by a given group of organisms provide an important class of molecular markers that have been proven very useful for evolutionary/taxonomic studies [23,24,26,27,34,35,52]. Due to the rare and discrete nature of genetic changes giving rise to the CSIs, the presence or absence of CSIs in different lineages (or proteins) is generally not affected by the factors that can confound the inferences from phylogenetic trees [24,27,28,29,30]. Hence, the CSIs provide powerful means for demarcating different groups of organisms in molecular terms and for understanding their evolutionary relationships [22,24,25,26,34,52]. Therefore, a major focus of this study was on conducting comprehensive analysis of protein sequences from Glires and related species to identify CSIs which are specific for different groups/clades within this grandorder. The results from these studies presented here, have identified 41 novel CSIs that are uniquely found in either all Glires species or are specific for its different orders, suborders, and families. Additionally, some of the identified CSIs also provide information regarding the interrelationships among different suborders/families of Rodentia. A brief description of the specificities and other characteristics of the identified CSIs is provided below.

### 3.3. Molecular Signatures Specific for the Glires, Rodentia and Lagomorpha

**Figure 2:**
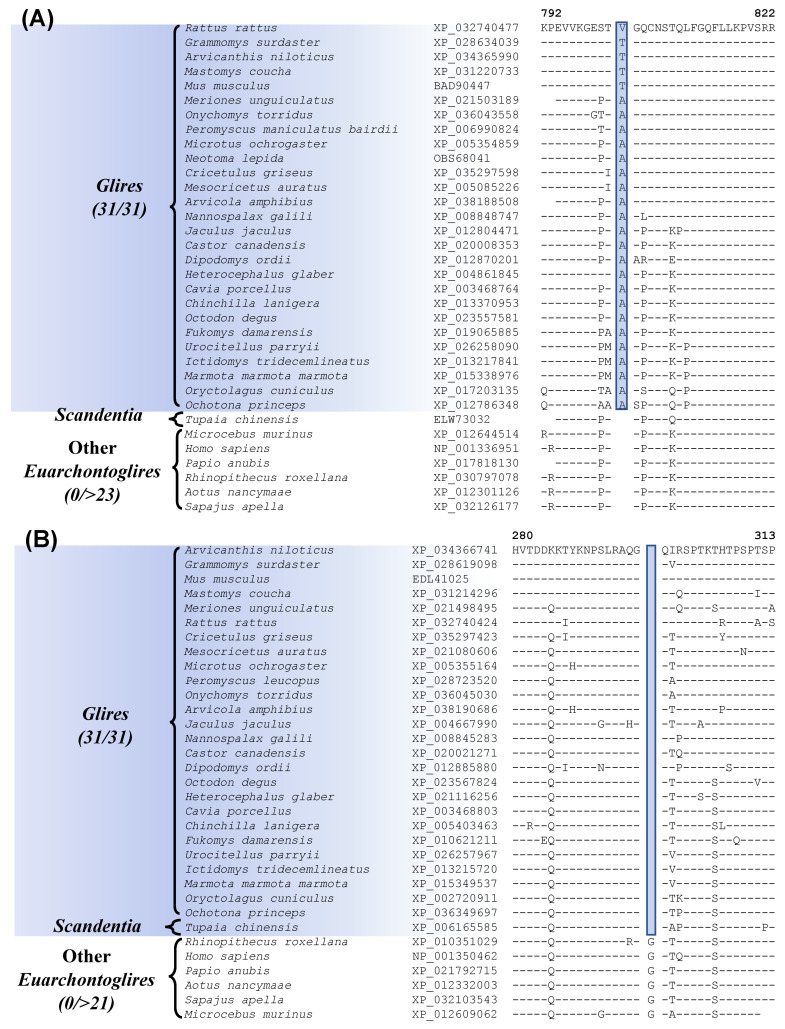
Partial sequence alignments of (**A**) junctional protein associated with coronary artery disease, containing a 1 aa insertion that is specific for the Glires; and (**B**) adenylyl cyclase-associated protein 2 containing a 1 amino acid deletion that is uniquely shared by the Glires and *Tupaia chinensis* (Scandentia). The accession numbers of the sequences are shown in the second column and the dashes (–) in the alignments indicate sequence identity with the amino acid present in the top row.

The grandorder Glires encompasses both Rodentia and Lagomorpha orders [8,13,16,20]. Our work has identified one CSI which is uniquely present in all sequenced Glires species (Figure 2A, Table 1). In Figure 2A, we show partial sequence information for the protein “junctional protein associated with coronary artery disease”, where a 1 aa insertion (highlighted) within a conserved region, is uniquely found in all Glires species but not in any other Euarchontoglires or other mammalian species examined. The dashes (–) in the alignment denote identity with the amino acid found on the top line. This CSI is present in a highly conserved region of the protein and constitutes a reliable molecular marker specific for the Glires. The protein in which this CSI is found colocalizes with the adhesion molecule VE-cadherin and is a component of endothelial cell–cell junctions. The protein containing this CSI shows an association with coronary artery disease, as implied by its name [53]. Kriegs et al. [20] have also previously identified several retroposon insertions that are specific for the Glires clade. 

In addition to this CSI specific for the Glires, we have also discovered one CSI, which is commonly shared by the Glires and *Tupaia chinensis*, a tree shrew species belonging to the order Scandentia. The sequence information for this CSI is shown in Figure 2B. In this case, a 1 aa deletion in a conserved region of the protein “adenylyl cyclase-associated protein 2” (CAP2) is uniquely shared by different Glires species and *Tupaia chinensis* (tree shrew) but not by any other Euarchontoglires or other mammalian/vertebrate species examined. The shared presence of this CSI by these two groups of species provides suggestive evidence that they may be specifically related to each other. However, as the clade consisting of these species is poorly supported in our phylogenetic tree, further evidence is needed to resolve this relationship. 

**Figure 3:**
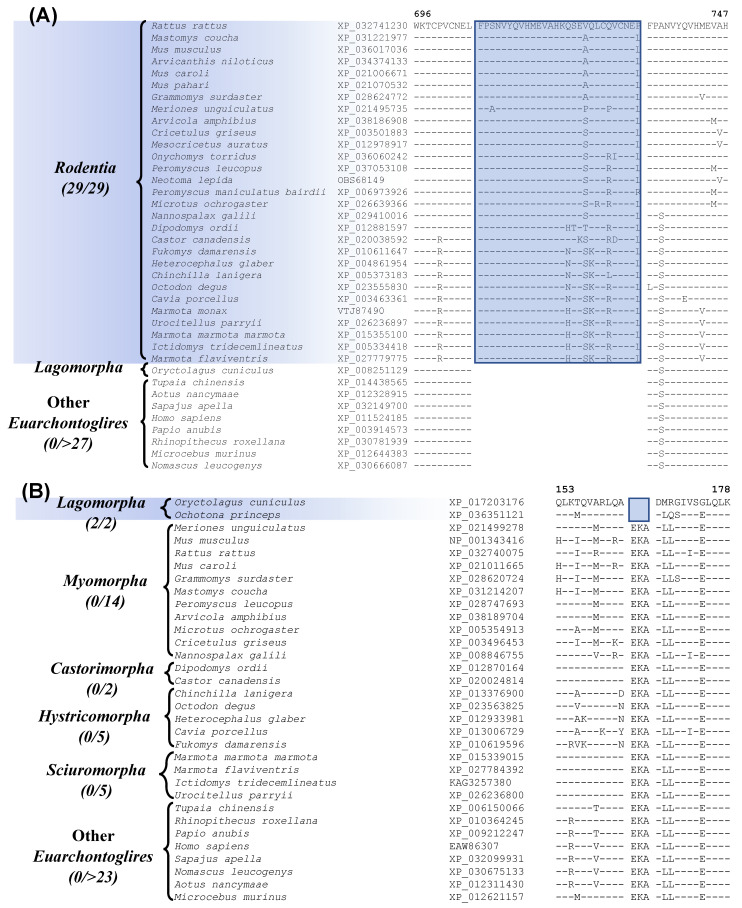
Partial sequence alignment of (**A**) activity-dependent neuroprotector homeobox protein 2 highlighting a 29 aa insertion that is specific for the species from the order Rodentia, and (**B**) partial sequence alignment of the protein optineurin, containing a 3 amino acid deletion, which is specific for the order Lagomorpha. Other details are the same as in Figure 1. Sequence information is shown here for only a limited number of outgroup species, but more detailed information is presented in the supplemental figures. Sequence information for three other CSIs specific for *Lagomorpha* is presented in Figures S5–S7 and summarized in Table 1.

Our analysis has also identified one prominent CSI that is Rodentia-specific. Sequence alignment containing this CSI is shown in Figure 3A. In this instance, a 29 aa insertion is present within a conserved region of the protein “activity-dependent neuroprotector homeobox protein 2”, that is specifically found in all sequenced Rodentia species, but it is not present in any other Euarchontoglires, or other mammalian/vertebrate species examined. It should be noted that most rodents and other species contain two homologs of this protein, and this CSI is found only in the homebox protein 2. This large indel provides a reliable molecular synapomorphy, indicating the monophyly of the order Rodentia and distinguishing it from other Euarchontoglires. The monophyly of Rodentia is also supported by 7 retroposon insertions identified in an earlier study [8]. Furthermore, four additional CSIs identified by our work are specific for the order Lagomorpha and sequence alignment for one of these CSIs is presented in Figure 3B. In the example shown, a 3 aa deletion in a conserved region of the protein Optineurin [54], is only present in the two species from the order Lagomorpha but is not found in any Rodentia or other Euarchontoglires species.

Besides this CSI, three other CSIs which are specific for Lagomorpha are found in the proteins U3 small nucleolar RNA-associated protein 6 homolog, ankyrin repeat and KH domain-containing protein 1, and prickle-like protein 1. Sequence information for these three CSIs, as well as more detailed sequence information for the CSI shown in Figure 3B, is presented in Figures S4–S7 and some of their characteristics are summarized in Table 1. These four CSIs provide reliable molecular markers distinguishing the order Lagomorpha from other Euarchontoglires. Recently, Sparwell et al. have also identified 4 transposon insertions that are specific for Lagomorpha [55].

### 3.4. Molecular Signatures Specific for the Rodentia Suborders 

**Figure 4:**
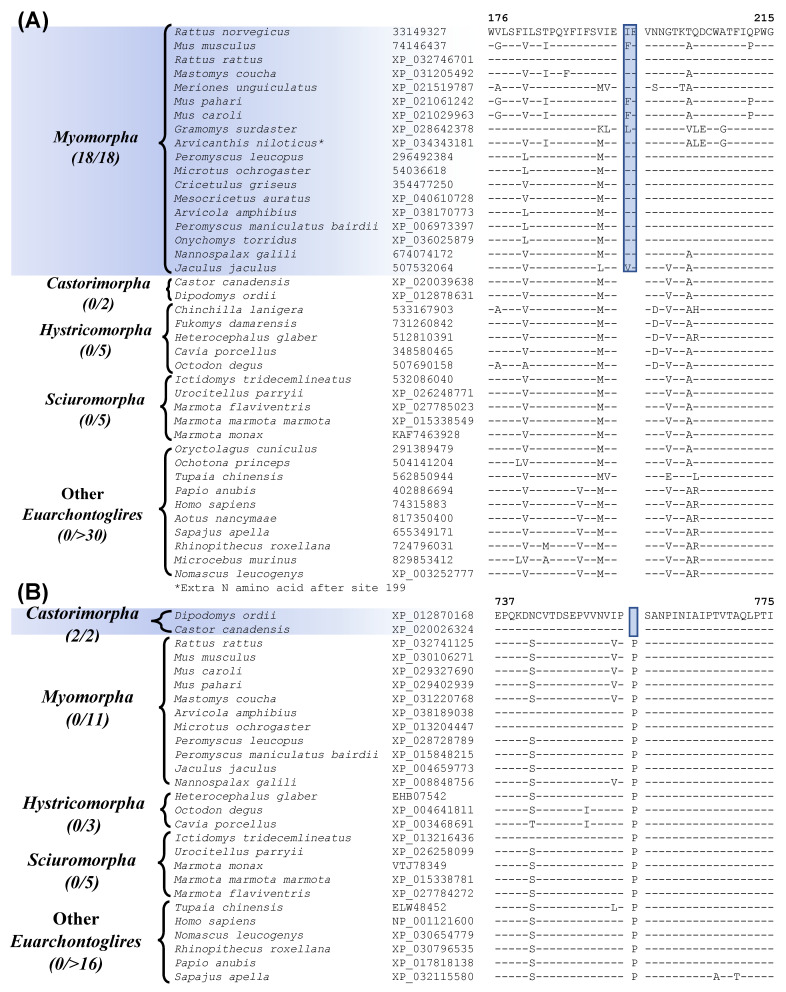
Partial sequence alignment of (**A**) the protein vasopressin V1a receptor, containing a 2 aa insertion that is specific for the suborder Myomorpha, and (**B**) the protein zinc finger E-box-binding homeobox 1, containing a 1 aa deletion that is specific for the suborder Castorimorpha. Other details are same as in Figure 1. Sequence information for additional CSIs specific for these two suborders are provided in Figures S8–S13 and summarized in Table 2.

**Figure 5:**
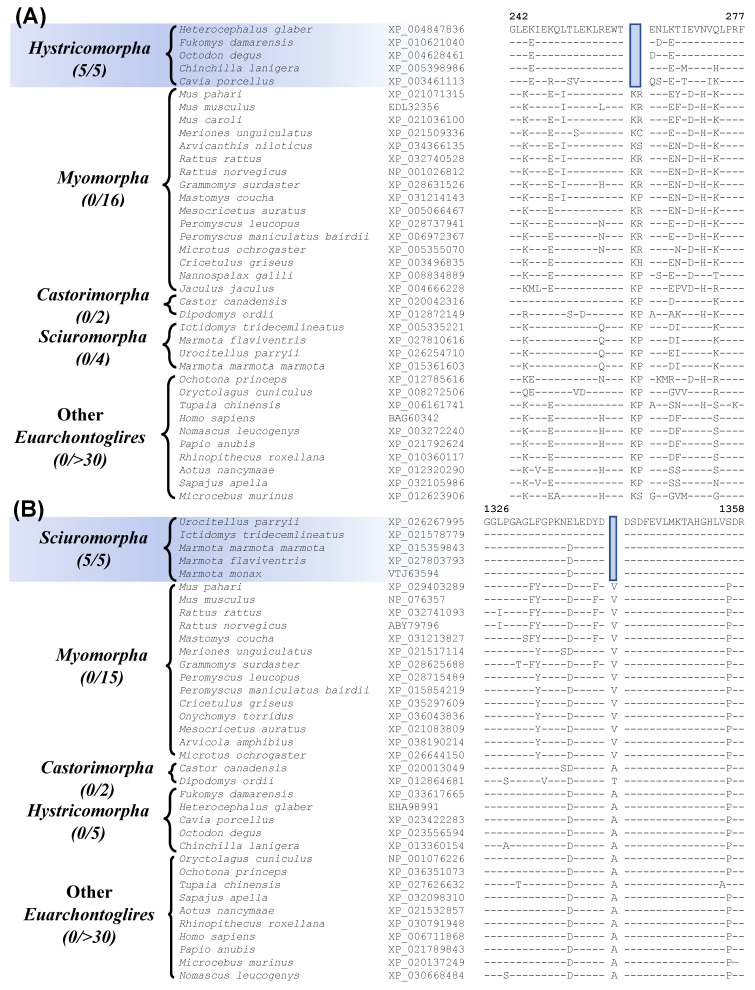
Partial sequence alignment of (**A**) the protein leukocyte elastase inhibitor A containing a 2 aa deletion that is specific for members of the suborder Hystricomorpha, and (**B**) excerpts from the sequence alignment of the protein ryanodine receptor 2 containing a 1 aa deletion that is specific for the suborder Sciuromorpha. Other details are the same as described in legend of Figure 1. Sequence information for other CSIs specific for the suborders Hystricomorpha and Sciuromorpha are provided in supplemental Figures S15–S29 and some characteristics are summarized in Table 2.

Our analyses have also uncovered many CSIs that are specific for different suborders of Rodentia (Figure 4 and Figure 5 and Table 2) and clarifying their evolutionary relationships. The suborder Myomorpha constitutes the largest group within the order Rodentia. Our work has identified 4 CSIs that are specific for Myomorpha demarcating this clade in molecular terms. One example of a CSI specific for the suborder Myomorpha is shown in Figure 4A (and Figure S8). In the example shown above, a 2 aa insertion (highlighted) is present in the vasopressin V1a receptor protein, that is only found in different Myomorpha species but is not found in any other Rodentia species or in other Euarchontoglires. The other proteins containing the CSIs specific for Myomorpha are, nck-associated protein 5-like isoform X1, ATP-dependent DNA helicase DDX11 isoform 1, and F-actin-uncapping protein. Sequence information for these other CSIs is presented in Figures S9–S11 and some of their characteristics are summarized in Table 2. Two other CSIs identified by our studies are specific for the suborder Castorimorpha. Sequence information for one of these CSIs, consisting of a 1 aa deletion in the protein “zinc finger E-box-binding homeobox 1” [56] is presented in Figure 4B (and Figure S12)**.** The highlighted CSI is only present in members of Castorimorpha and is not found in any other members of the Euarchontoglires. The other Castorimorpha-specific CSI is found in the protein “cAMP-responsive element modulator” and sequence information for it is presented in Figure S13 and summarized in Table 2. These CSIs clearly distinguish the suborders Castorimorpha from Myomorpha, which was not resolved in an earlier study based on retroposons [8,12].

Our work has also identified 7 CSIs that are specific for the suborder Hystricomorpha for which genome sequence are available from 5 species. Figure 5A shows sequence information for one of these CSIs, where a 2 aa deletion is present in the protein “leukocyte elastase inhibitor A”. This protein plays a role in cell migration and implicated in inflammatory lung and bowel diseases [57]. The CSI shown in Figure 5A is present in all five Hystricomorpha species but absent in all other Rodentia and Euarchontoglires. More detailed sequence information for this CSI and the other CSIs specific for the suborder Hystricomorpha is presented in Figures S14–S20 and some of their characteristics are summarized in Table 2. 

Another 9 CSIs identified by our work, are specific for the suborder Sciuromorpha. Sequence information for one of these CSIs consisting of a 1 aa deletion in the protein “ryanodine receptor 2” is shown in Figure 5B. The described CSI is only present in members of the suborder Sciuromorpha and not found in any other Euarchontoglires species. The protein “ryanodine receptor 2”, in which this CSI is found, is a calcium release channel that is present in the heart and brain and mutations in this gene have been linked with leaky channels that can lead to sudden cardiac arrest and seizures [58]. More detailed sequence information for this CSI and the other CSIs that are specific for the suborder Sciuromorpha is provided in supplemental Figures S21–S29 and some of their characteristics are summarized in Table 2.

**Table 2:** Characteristics of the CSIs specific for different suborders of Rodentia.

Protein Name	Accession No.	Figure Number	Indel Size	Indel Location	Specificity
vasopressin V1a receptor	74146437	Figure 4A Figure S8	2 aa Ins	176–215	Myomorpha
nck-associated protein 5-like isoform X1	XP_006521185	Figure S9	1 aa Del	584–619	
ATP-dependent DNA helicase DDX11 isoform 1	NP_001335221	Figure S10	3 aa Ins	481–514	
F-actin-uncapping protein LRRC16A	BAC31591	Figure S11	1 aa Del	1150–1174	
zinc finger E-box-binding homeobox 1	XP_030106271	Figure 4B Figure S12	1 aa Del	737–775	Castorimorpha
cAMP-responsive element modulator	XP_030106165	Figure S13	1 aa Del	150–186	
leukocyte elastase inhibitor A	EDL32356	Figure 5A Figure S14	2 aa Del	242–277	Hystricomorpha
sterol regulatory element-binding protein cleavage-activating protein	AAH70437	Figure S15	1 aa Del	1040–1077	
early endosome antigen 1 isoform X1	XP_006513587	Figure S16	1 aa Del	58–91	
tudor domain-containing protein 1	NP_001002238	Figure S17	1 aa Del	42–74	
tudor domain-containing protein 1	AAI29955	Figure S18	6 aa Del	669–703	
autophagy-related protein 9A isoform a	XP_011236992	Figure S19	2 aa Ins	659–687	
probable small intestine urate exporter	XP_006516763	Figure S20	2 aa Ins	429–469	
ryanodine receptor 2	NP_076357	Figure 5B Figure S21	1 aa Del	1326–28	Sciuromorpha
A disintegrin and metallo-proteinase with thrombospondin motifs 13 isoform 1 preproprotein	NP_001001322	Figure S22	2 aa Del	1072–1109	
telomerase-binding protein EST1A	EDL12790	Figure S23	1 aa Del	472–503	
oxysterol-binding protein-related protein 8 isoform b	XP_006513700	Figure S24	1 aa Ins	816–844	
rab-3A-interacting protein isoform 2	NP_001003950	Figure S25	1 aa Del	36–67	
dual specificity protein phosphatase CDC14B	XP_036013890	Figure S26	2 aa Ins	335–370	
zinc finger protein 385A	NP_038894	Figure S27	1 aa Del	7–44	
rho family-interacting cell polarization regulator 2	BAE37527	Figure S28	1 aa Ins	336–371	
rho family-interacting cell polarization regulator 2	XP_006516650	Figure S29	5 aa Del	587–625	
ATP-dependent DNA helicase DDX11 isoform 1	XP_006524473	Figure 6 Figure S30	1 aa Del	262–289	Myomorpha and Castorimorpha
voltage-dependent L-type calcium channel subunit beta-2	XP_006497377	Figure S31	1 aa Ins	375–401	

**Figure 6:**
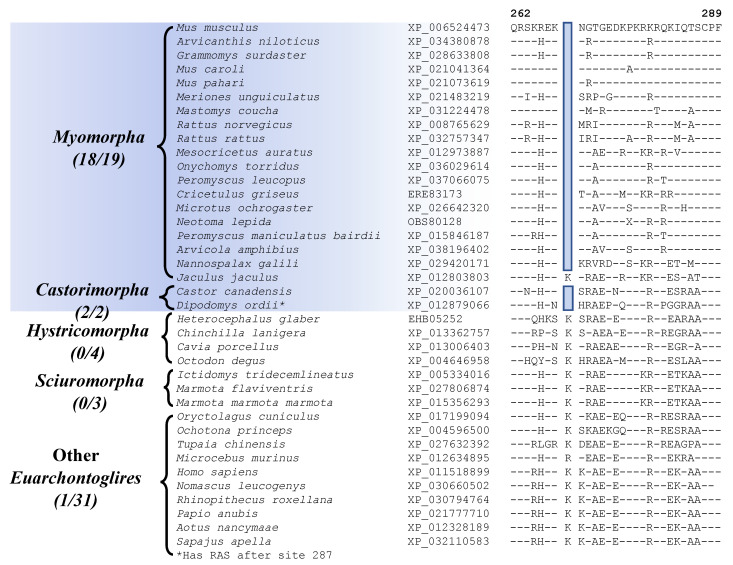
Partial sequence alignment for the protein ATP-dependent DNA helicase DDX11 isoform 1, showing a 1 amino acid deletion that is commonly shared by all Myomorpha and Castorimorpha species except the deep-branching *Jaculus jaculus*. Sequence information for another CSI that is commonly shared by species from these two suborders is provided in Figure S31.

Two other CSIs identified by our analyses are commonly shared by most of the species from the suborders Myomorpha and Castorimorpha, supporting a sister relationship between these suborders that is observed in Figure 1. Sequence information for one of these CSIs consisting of a 1 aa deletion in the protein “ATP-dependent DNA helicase DDX11 isoform 1” is shown in Figure 6 (and Figure S30). Interestingly, while this CSI is present in all other Myomorpha and Castorimorpha species, it is lacking in *Jaculus jaculus*, which constitutes the deepest branching lineage (family Dipodoidea) within the suborder Myomorpha. Another CSI that is commonly shared by 18 of the 19 Myomorpha species and one of the two Castorimorpha species (*Dipodomys ordii*) is found in the protein “voltage-dependent L-type calcium channel subunit beta-2” (Sup. Figure S31). Despite isolated exceptions, whose evolutionary significances are at present unclear, these two CSIs provide evidence suggesting that the species from these two suborders are more closely related to each other than to the other suborders of Rodentia. A close relationship of the Myomorpha and Castorimorpha is also supported by a retroposon insertion identified in earlier work [8].

### 3.5. Molecular Signatures Specific for the Family Level Clades in Myomorpha

**Figure 7:**
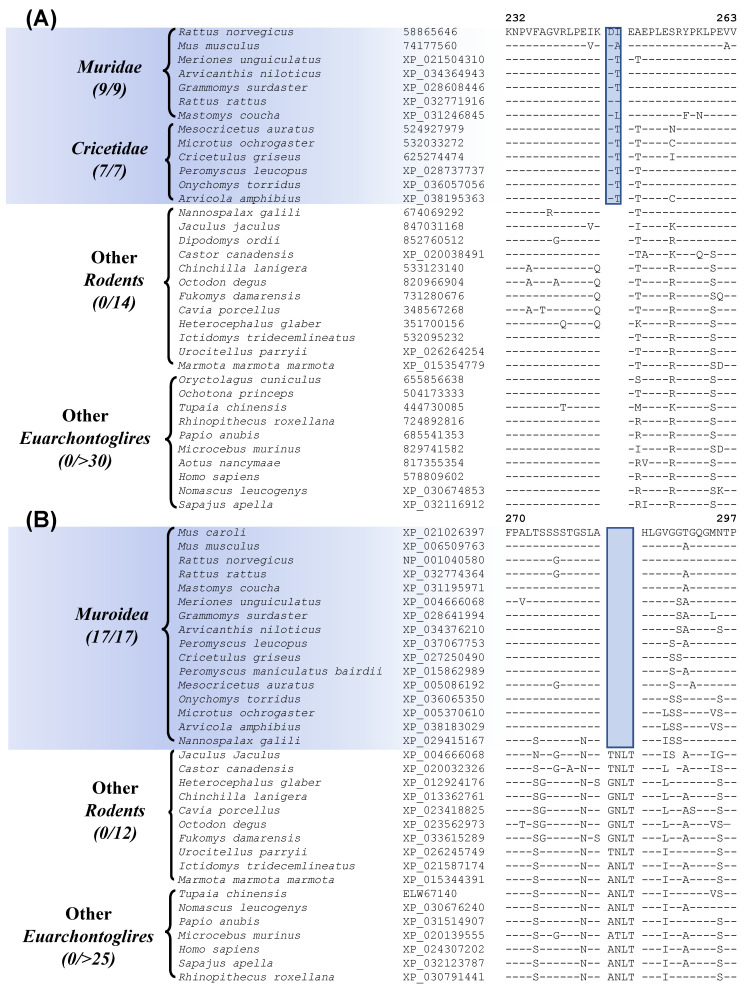
(**A**) Partial sequence alignment of the protein cyclin-dependent kinase-like 2, containing a 2 amino acid insertion that is commonly shared by the families Muridae and Cricetidae. (**B**) Excerpts from the sequence alignment of the protein CREB-regulated transcription coactivator 1 highlighting a 4 amino acid deletion that is specific for the superfamily Muroidea. Sequence information for the additional CSIs showing similar specificities is presented in Figures S32–S41 and some of their characteristics are summarized in Table 3.

Myomorpha is the largest suborder within Rodentia, and more than half of the available sequences are for Myomorpha species. This suborder is comprised of four families Muridae, Cricetidae, Spalacidae, and Dipodoidea. Our analyses have identified six CSIs that are uniquely shared by members of the families Muridae and Cricetidae, which show a close relationship to each other in our phylogenetic tree (Figure 1). Sequence information for one of these CSIs specific for these two families is shown in Figure 7A. In this instance, a 2 aa insertion in the protein “cyclin-dependent kinase-like 2”, which is an important cell growth regulator [59], is commonly shared by all 16 species from the families Muridae and Cricetidae but not found in any other Rodentia as well as other Euarchontoglires species. Sequence information for the other CSIs that are specific for these two families is provided in supplemental Figures S32–S37 and some of their characteristics are summarized in Table 3.

Within Myomorpha, the families Muridae, Cricetidae, and Spalacidae are known to show a closer relationship and they form the superfamily Muroidea. The members of these three families also exhibit a close relationship in the phylogenetic tree constructed in this work (Figure 1). A specific relationship between these families is also supported by 4 CSIs identified in this study. Sequence information for one of these CSIs, consisting of a 4 aa deletion in the protein “CREB-regulated transcription coactivator 1” that is uniquely present in the members of these three families is presented in Figure 7B (and Figure S38). Sequence information for the other three CSIs that are specific for these two families is provided in supplemental Figures S39–S41 and some of their characteristics are summarized in Table 3.

**Table 3:** Characteristics of the CSIs that are Specific for the Myomorpha families.

Protein Name	Accession No.	Figure Number	Indel Size	Indel Location	Specificity
cyclin-dependent kinase-like 2	74177560	Figure 7A Figure S32	2 aa Ins	232–263	Muridae and Cricetidae
nck-associated protein 5-like	NP_001001884	Figure S33	1 aa Del	782–817	
lysosomal acid glucosylceramidase	568921788	Figure S34	1 aa Del	276–308	
cAMP-responsive element modulator	NP_001104322	Figure S35	1 aa Del	61–91	
cyclin-dependent kinase 13	XP_006516830	Figure S36	1 aa Del	549–582	
voltage-dependent L-type calcium channel subunit beta-2	XP_036013681	Figure S37	1 aa Del	444–474	
CREB-regulated transcription coactivator 1	XP_006509763	Figure 7B Figure S38	4 aa Del	270–297	Muroidea
striatin-interacting proteins 2	148681817	Figure S39	1 aa Ins	86–119	
disco-interacting protein 2 homolog C	BAC29340	Figure S40	4 aa Ins	953–988	
zinc finger protein 40	XP_006516902	Figure S41	1 aa Del	2511–2535	

## 4. Discussion

The order Rodentia contains approximately 40% of the extant mammalian species, which are highly abundant and native to every continent except Antarctica [2]. Rodent species are closely associated with humans in daily lives and due to their close genetic and metabolic similarity to humans, they are widely used as animal models for genetic and biochemical studies related to humans [4,5]. Although some rodent species have the reputation for carrying diseases that can be passed on to humans [60], because of their ease of reproduction and small sizes, rodent species are indispensable as animal models for testing the toxicity as well as therapeutic effectiveness of different drugs and chemicals prior to human usage [4,5]. Thus, it is imperative to have a clear understanding of the interrelationships of different species comprising this important clade of animals. The emergence of the genomic era has brought much clarity to the classification of Rodentia over the earlier classification schemes based on morphological characteristics [2]. However, certain aspects of rodent phylogeny, including the branching order of the four main suborders/clades within the Rodentia are still not clearly resolved [1,9]. 

In the present work, we have conducted both phylogenetic and molecular markers-based analyses on the genome sequences from Glires species, to further understand the evolutionary relationships among these species. In a phylogenetic tree constructed based on concatenated sequences of 25 single copy conserved proteins, members of the orders Rodentia and Lagomorpha both formed strongly supported monophyletic clades, showing sister group relationship to each other. These results are in accordance with the earlier studies [1,9,16,20]. In our phylogenetic tree, species from the four Rodentia suborders, viz., Castorimorpha, Hystricomorpha, Myomorpha, and Sciuromorpha, also formed strongly supported monophyletic clades, like those seen in some earlier studies [1,8,9,10,11,12,13,14]. Within the order Rodentia, the suborder Sciuromorpha branched deeply in comparison to the other suborders, however, the statistical support for this branching was low. Additionally, the tree also showed a sister relationship between the suborders Castorimorpha and Myomorpha, which has also been observed in earlier studies [9,12]. 

**Figure 8:**
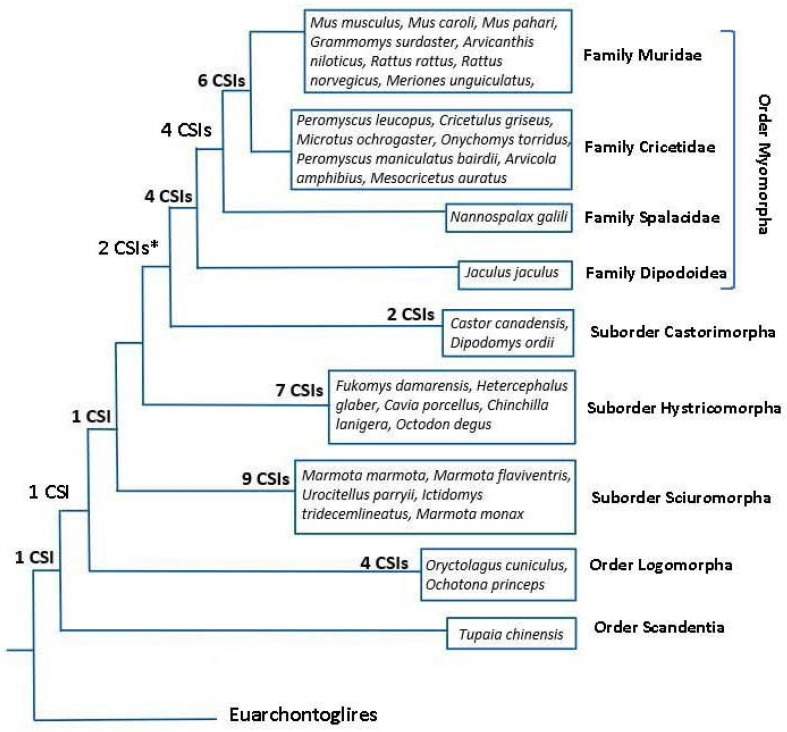
A conceptual diagram summarizing the overall evolutionary relationships among the Rodent/Glires species based on their branching in a phylogenetic tree constructed from sequences of 25 conserved proteins, and the clade specificities of the CSIs identified in this work. The numbers of CSIs, which are specific for different clades are indicated on the nodes. The asterisk (*) indicates that one of these CSIs was lacking in the species *Jaculus jaculus*.

However, the main focus of this work was on identifying rare genetic changes consisting of CSIs in conserved proteins that are uniquely shared by different groups/clades of rodents. As noted in Introduction, rare genetic changes (RGCs) in genes/proteins such as CSIs or retroposons provide important tools for phylogenetic studies. Unlike the phylogenetic trees, where the interrelationship among species is dependent upon large number of variables, the inferences based upon the shared presence/absence of RGCs are less prone to the influences of variables that can confound the reliability of inferences from phylogenetic trees [23,30,50,52]. Hence, the CSIs in protein sequences have proven useful in resolving several important evolutionary relationships, which had proven difficult to resolve by phylogenetic means [23,24,29,33,35]. In the present study, we have identified 41 novel CSIs in different proteins that except for an isolated exception are exclusively shared by different species from specific clades of rodents/glires. In Figure 8, we present an overall summary of the clade or group specificities of the identified CSIs. In this Figure, the CSIs, which are specific for different groups/clades are laid upon the phylogenetic tree for Glires species constructed in this work. As seen from Figure 8, a large proportion of the discovered CSIs are specific for different suborders or families of rodents. In contrast to these CSIs, which are specific for different observed clades, except for an isolated exception noted here, we have not come across other CSIs that supported alternate relationships among these species. Thus, based on these CSIs and the constructed phylogenetic tree, all main suborders of Rodentia (viz. Myomorpha, Castorimorpha, Sciuromorpha, Hystricomorpha), as well as some family level clades of Myomorpha (viz. (Muridae *+* Cricetidae) and Muroidea) can now be reliably distinguished/demarcated from each other in molecular term based on multiple highly specific markers. Our work has also identified 1 CSI each that are specific for the Glires and Rodentia clade and 4 CSIs, which are exclusive for the order Lagomorpha. Additionally, some identified CSIs were useful in indicating the relationships among different clades. Of these CSIs, two supported a sister relationship of the suborders Myomorpha and Castorimorpha, which branched together in our phylogenetic tree [12].

Our work has also identified one CSI that is specifically shared by all Glires species and *Tupaia chinensis*, a species belonging to the order Scandentia, which is a part of the grandorder Euarchonta suggesting a closer relationship between these two groups However, as different studies on the phylogenetic placement of order Scandentia have yielded conflicting results, possibly due to incomplete lineage sorting [18,20,42], further work is needed to resolve this relationship.

Several earlier studies have used the presence or absence of retroposons to infer the relationships among rodents and other related species [8,12,20,42,52]. Our work on CSIs that are specific for different clades of rodents is complementary to this earlier work. For example, in the study by Churakov et al. [8] based on the identification of retroposons for Rodentia clades, the suborders Myomorpha and Castorimorpha were not distinguished. However, the work presented here as well as another recent study [12] now provide evidence that these two suborders are distinct and support a sister group relationship between them. Another aspect of rodent phylogeny not reliably resolved by means phylogenetic trees concerned the placement of the suborders Hystricomorpha and Sciuromorpha. However, Churakov et al. [8] identified 8 retroposons that were commonly shared by the suborders Myomorpha, Castorimorpha, and Hystricomorpha, thus placing the suborder Sciuromorpha in the basal position, which is also seen in our phylogenetic tree. Together, these results support the inference that the order Hystricomorpha lies in between the clade consisting of the suborders (Myomorpha-Castorimorpha) and Sciuromorpha. Thus, the work presented here in conjunction with the earlier studies ([8,12,55] consolidates and advances our understanding of the overall evolutionary relationships among the Rodentia/Glires species.

Lastly, it is important to point out an important aspect of the molecular markers specific for different groups of rodents that have been identified in this work. Unlike the retroposons, these molecular markers are present in highly conserved regions of various proteins that carry out important cellular functions. Extensive earlier work on CSIs has shown that these conserved molecular characteristics play important and often essential functions in the organisms where they are found [35,37,38,40,61]. Most of the studied CSIs in protein sequences are localized in the surface loops of proteins, which play important roles in mediating novel protein–protein or protein–ligand interactions that are essential or important for the CSI-containing organisms [38,39,41,61]. As briefly noted during the description of various identified CSIs, many of the proteins harboring the CSIs that have been identified in this work carry out important function related to various diseases. Thus, it should be of much interest to investigate the functional significance of these CSIs in the functioning of these proteins. Such studies could reveal interesting differences in the functioning of these proteins between rodents and humans. Lastly, extensive work on CSIs indicate that they possess high degree of predictive ability to be found in other members of the clades for which they are specific [25,30,34]. As the identified CSIs are all present in highly conserved regions, the presence/absence of these CSIs in other rodent species can be readily examined by means of different commonly used experimental techniques viz., PCR-based, q-PCR-based, as well as by in silico BLAST searches examining the presence of these CSIs in genomic sequence data. The CSIs-based approaches have been used previously for developing novel diagnostic tests for several important bacterial pathogens [61,62].

## Figures and Tables

**Table 1:** Characteristics of the CSIs specific for the Glires, Rodentia, and Lagomorpha Clades.

Protein Name	Accession No.	Figure Number	Indel Size	Indel Location	Specificity
junctional protein associated with coronary artery disease	BAD90447	Figure 2A Figure S1	1aa Ins	792–822	Glires
adenylyl cyclase-associated protein 2	EDL41025	Figure 2B Figure S2	1aa Del	280–313	Glires and Scandentia
activity-dependent neuroprotector homeobox protein 2	XP_036017036	Figure 3A Figure S3	28aa Ins	696–747	Rodentia
optineurin	NP_001343416	Figure 3B Figure S4	3aa Del	153–178	Lagomorpha
U3 small nucleolar RNA-associated protein 6 homolog	74146777	Figure S5	1aa Ins	192–227	
ankyrin repeat and KH domain-containing protein 1	NP_780584	Figure S6	1aa Ins	1893–1927	
prickle-like protein 1	NP_001028389	Figure S7	3aa Del	553–586	

## Data Availability

The data presented in this study are available in publicly accessible repository (https://www.ncbi.nlm.nih.gov/genome/, accessed on 1 April 2021) and Supplementary Material here.

## Supplementary Materials

The following are available online at https://www.mdpi.com/article/10.3390/genes13020288/s1. Table S1. Name and accession numbers of proteins used for phylogenetic analysis. Figure S1. Partial sequence alignments of the protein “junctional protein associated with coronary artery disease”, showing a 1 amino acid insertion that is specific for the Glires. Figure S2. Partial sequence alignments of a conserved region of the protein adenylyl cyclase-associated protein 2, showing a 1 amino acid deletion that is commonly shared by the Glires and Scandentia. Figure S3. Partial sequence alignments of a conserved segment of the protein activity-dependent neuroprotector homeobox protein 2, showing a 28 amino acid insertion that is specific for the order Rodentia. Figure S4. Partial sequence alignments of a conserved segment of the protein optineurin, showing a 3 amino acid deletion that is present in the order Lagomorpha. Figure S5. Partial sequence alignments of a conserved region of the protein U3 small nucleolar RNA-associated protein 6 homolog, showing a 1 amino acid insertion that is specific for the order Lagomorpha. Figure S6. Partial sequence alignments of a conserved region of the protein ankyrin repeat and KH domain-containing protein 1, showing a 1 amino acid insertion that is that is specific for the order Lagomorpha. Figure S7. Partial sequence alignments of a conserved region of the protein prickle-like protein 1, showing a 3 amino acid deletion that is specific for the order Lagomorpha. Figure S8. Partial sequence alignments of a conserved region of the protein vasopressin V1a receptor, showing a 2 amino acid insertion that is specific for the suborder Myomorpha. Figure S9. Partial sequence alignments of a conserved region of the protein nck-associated protein 5-like isoform X1 showing a 1 amino acid deletion that is specific for the suborder Myomorpha. Figure S10. Partial sequence alignments of a conserved region of the protein ATP-dependent DNA helicase DDX11 isoform 1, showing a 3 amino acid insertion that is specific for the suborder Myomorpha. Figure S11. Partial sequence alignments of a conserved region of the protein F-actin-uncapping protein LRRC16A, showing a 1 amino acid deletion that is specific for the suborder Myomorpha. Figure S12. Partial sequence alignments of a conserved region of the protein zinc finger E-box-binding homeobox 1, showing a 1 amino acid deletion that is specific for the suborder Castorimorpha. Figure S13. Partial sequence alignments of a conserved region of the protein cAMP-responsive element modulator, showing a 1 amino acid deletion that is specific for the suborder Castorimorpha. Figure S14. Partial sequence alignments of a conserved region of the protein leukocyte elastase inhibitor A, showing a 2 amino acid deletion that is specific for the suborder Hystricomorpha. Figure S15. Partial sequence alignments of a conserved region of the protein sterol regulatory element-binding protein cleavage-activating protein, showing a 1 amino acid deletion that is specific for the suborder Hystricomorpha. Figure S16. Partial sequence alignments of a conserved region of the protein early endosome antigen 1 isoform X1, showing a 1 amino acid deletion that is specific for the suborder Hystricomorpha. Figure S17. Partial sequence alignments of a conserved region of the protein tudor domain-containing protein 1, showing a 1 amino acid deletion that is specific for the suborder Hystricomorpha. Figure S18. Partial sequence alignments of a conserved region of the protein tudor domain-containing protein 1, showing a 6 amino acid deletion that is specific for the suborder Hystricomorpha. Figure S19. Partial sequence alignments of a conserved region of the protein autophagy-related protein 9A isoform a, showing a 2 amino acid insertion that is specific for the suborder Hystricomorpha. Figure S20. Partial sequence alignments of a conserved region of the protein probable small intestine urate exporter, showing a 2 amino acid insertion that is specific for the suborder Hystricomorpha. Figure S21. Partial sequence alignments of a conserved region of the protein ryanodine receptor 2, showing a 1 amino acid deletion that is specific for the suborder Sciuromorpha. Figure S22. Partial sequence alignments of a conserved region of the protein A disintegrin and metalloproteinase with thrombospondin motifs 13 isoform 1 preproprotein, showing a 2 amino acid deletion that is specific for the suborder Sciuromorpha. Figure S23. Partial sequence alignments of a conserved region of the protein telomerase-binding protein EST1A, showing a 1 amino acid deletion that is specific for the suborder Sciuromorpha. Figure S24. Partial sequence alignments of a conserved region of the protein oxysterol-binding protein-related protein 8 isoform b, showing a 1 amino acid insertion that is specific for the suborder Sciuromorpha. Figure S25. Partial sequence alignments of a conserved region of the protein rab-3A-interacting protein isoform 2, showing a 1 amino acid deletion that is specific for the suborder Sciuromorpha. Figure S26. Partial sequence alignments of a conserved region of the protein dual specificity protein phosphatase CDC14B, showing a 2 amino acid insertion that is specific for the suborder Sciuromorpha. Figure S27. Partial sequence alignments of a conserved region of the protein zinc finger protein 385A, showing a 1 amino acid deletion that is specific for the suborder Sciuromorpha. Figure S28. Partial sequence alignments of a conserved region of the protein rho family-interacting cell polarization regulator 2, showing a 1 amino acid insertion that is specific for the suborder Sciuromorpha. Figure S29. Partial sequence alignments of a conserved region of the protein rho family-interacting cell polarization regulator 2, showing a 5 amino acid deletion that is specific for the suborder Sciuromorpha. Figure S30. Partial sequence alignments of a conserved region of the protein ATP-dependent DNA helicase DDX11 isoform 1, showing a 1 amino acid deletion that is commonly shared by species from the suborders Myomorpha and Castorimorpha. Figure S31. Partial sequence alignments of a conserved region of the protein voltage-dependent L-type calcium channel subunit beta-2, showing a 1 amino acid insertion that is commonly shared by species from the suborders Myomorpha and Castorimorpha. Figure S32. Partial sequence alignments of a conserved region of the protein cyclin-dependent kinase-like 2, showing a 2 amino acid insertion that is commonly shared by species from the families Muridae and Cricetidae. Figure S33. Partial sequence alignments of a conserved region of the protein nck-associated protein 5-like, showing a 1 amino acid deletion that is commonly shared by species from the families Muridae and Cricetidae. Figure S34. Partial sequence alignments of a conserved region of the protein lysosomal acid glucosylceramidase, showing a 1 amino acid deletion that is commonly shared by species from the families Muridae and Cricetidae. Figure S35. Partial sequence alignments of a conserved region of the protein cAMP-responsive element modulator, showing a 1 amino acid deletion that is commonly shared by species from the families Muridae and Cricetidae. Figure S36. Partial sequence alignments of a conserved region of the protein cyclin-dependent kinase 13, showing a 1 amino acid deletion that is commonly shared by species from the families Muridae and Cricetidae. Figure S37. Partial sequence alignments of a conserved region of the protein voltage-dependent L-type calcium channel subunit beta-2, showing a 1 amino acid deletion that is commonly shared by species from the families Muridae and Cricetidae. Figure S38. Partial sequence alignments of a conserved region of the protein CREB-regulated transcription coactivator 1, showing a 4 amino acid deletion that is commonly shared by species from the superfamily Muroidea. Figure S39. Partial sequence alignments of a conserved region of the protein striatin-interacting proteins 2, showing a 1 amino acid insertion that is commonly shared by species from the superfamily Muroidea. Figure S40. Partial sequence alignments of a conserved region of the protein disco-interacting protein 2 homolog C, showing a 4 amino acid insertion that is commonly shared by species from the superfamily Muroidea. Figure S41. Partial sequence alignments of a conserved region of the protein zinc finger protein 40, showing a 1 amino acid deletion that is commonly shared by species from the superfamily Muroidea.
