# Supplementary material for: Conserved Signatures in Protein Sequences Reliably Demarcate Different Clades of Rodents/Glires Species and Consolidate Their Evolutionary Relationships
Source: Genes (Basel). 2022 Feb 1;13(2):288. doi: 10.3390/genes13020288 (PMC8871558; doi:10.3390/genes13020288)
Supplement: Supplementary file 1 [file genes-13-00288-s001.zip › genes-1561850-supplementary.pdf]

Table S1: Information for the proteins, whose concatenated sequences were used for phylogenetic studies. Each of these proteins was assessed to be present in a single copy in Glires species based on BlastP searches. The accession number are from *Mus musculus*

Protein Name	Accession no:	Protein Length (AAs)
60 kDa heat shock protein, mitochondrial	NP_001343441	573
78 kDa glucose-regulated protein	BAA11462	655
aminopeptidase O	NP_001276853	812
angiopoietin-related protein 7	NP_001034643	337
cyclin-O	NP_001074531	352
dendrin	NP_001013763	710
desmoplakin	NP_076331	2883
GAS2-like protein 2	NP_001013781	812
heat shock factor protein 5	NP_001038992	624
heat shock protein HSP 90-alpha	NP_034610	733
hippocampus abundant transcript-like protein 1	NP_001077370	507
isoleucine--tRNA ligase, cytoplasmic	NP_742012	1262
leucine-rich repeat-containing protein 52 precursor	NP_001013400	314
low-density lipoprotein receptor-related protein 2	NP_001074557	4660
lysine-specific demethylase PHF2	NP_035208	1096
lysophospholipid acyltransferase 1	NP_705774	492
PDZ domain-containing protein 8	NP_001028394	1147
platelet-activating factor receptor	NP_001074680	341
potassium voltage-gated channel subfamily A member 10	NP_001074609	511
probable G-protein coupled receptor 158	NP_001004761	1200
rho guanine nucleotide exchange factor 17	NP_001074585	2057
RNA-binding protein 15	NP_001039272	962
sodium/hydrogen exchanger 2	NP_001028461	814
transmembrane protein 102	NP_001028605	509
zinc finger and BTB domain-containing protein 9	NP_001005916	459

Group	Species	Accession	792	CSI	822
Glires (31/31)	*Rattus rattus*	XP_032740477	KPEVVKGEST	V	GQCNSTQLFGQFLLKPVSRR
	Grammomys surdaster	XP_028634039	----------	T	--------------------
	Rattus norvegicus	XP_008770007	----------	-	--------------------
	Arvicanthis niloticus	XP_034365990	----------	T	--------------------
	Mastomys coucha	XP_031220733	----------	T	--------------------
	Mus musculus	BAD90447	----------	T	--------------------
	Mus pahari	XP_021070444	---------I	T	-------H------------
	Meriones unguiculatus	XP_021503189	------P-	A	--------------------
	Onychomys torridus	XP_036043558	-------GT-	A	--------------------
	Peromyscus maniculatus bairdii	XP_006990824	--------T-	A	--------------------
	Microtus ochrogaster	XP_005354859	--------P-	A	--------------------
	Neotoma lepida	OBS68041	--------P-	A	--------------------
	Cricetulus griseus	XP_035297598	---------I	A	--------------------
	Mesocricetus auratus	XP_005085226	---------I	A	--------------------
	Arvicola amphibius	XP_038188508	------P-	A	--------------------
	Nannospalax galili	XP_008848747	--------P-	A	-L------------------
	Jaculus jaculus	XP_012804471	--------P-	A	-P---KP-------------
	Castor canadensis	XP_020008353	--------P-	A	-P---K--------------
	Dipodomys ordii	XP_012870201	--------P-	A	AR---E--------------
	Heterocephalus glaber	XP_004861845	----------	A	-P---K--------------
	Cavia porcellus	XP_003468764	--------P-	A	-P---K--------------
	Chinchilla lanigera	XP_013370953	--------P-	A	-P---K--------------
	Octodon degus	XP_023557581	--------P-	A	-P---K--------------
	Fukomys damarensis	XP_019065885	--------PA	A	-P---K--------------
	Marmota flaviventris	XP_027784256	--------PM	A	-P---K-P------------
	Urocitellus parryii	XP_026258090	--------PM	A	-P---K-P------------
	Ictidomys tridecemlineatus	XP_013217841	--------PM	A	-P---K-P------------
	Marmota marmota marmota	XP_015338976	--------PM	A	-P---K-P------------
	Marmota monax	KAF7473951	--------PM	A	-P---K-P------------
	Oryctolagus cuniculus	XP_017203135	Q-------TA	A	-S---Q-P------------
	Ochotona princeps	XP_012786348	Q-------AA	A	SP---Q-P------------
Scandentia	*Tupaia chinensis*	ELW73032	------P-		-P---Q--------------
Other *Euarchontoglires*	*Carlito syrichta*	XP_008069436	--------P-		-P---K--------------
	Microcebus murinus	XP_012644514	R-------P-		-P---K--------------
	Galeopterus variegatus	XP_008583742	--------T-		-P---Q-P------------
	Homo sapiens	NP_001336951	-R------P-		-P---K--------------
	Macaca mulatta	XP_001082542	-R------P-		-P---K--------------
	Papio anubis	XP_017818130	------P-		-P---K--------------
	Rhinopithecus roxellana	XP_030797078	-R------P-		-P---K--------------
	Rhinopithecus bieti	XP_017725204	-R------P-		-P---K--------------
	Macaca nemestrina	XP_011736632	-R------P-		-P---K--------------
	Macaca fascicularis	XP_005564954	-R------P-		-P---K--------------
	Chlorocebus sabaeus	XP_008000865	-R------P-		-P---K--------------
	Pan paniscus	XP_034786108	------P-		-P---K--------------
	Pan troglodytes	XP_009456473	-R------P-		-P---K--------------
	Cercocebus atys	XP_011939989	------P-		-P---K--------------
	Gorilla gorilla gorilla	XP_018890981	-R------P-		-P---K--------------
	Pongo abelii	XP_024109622	-R------P-		-P---K--------------
	Propithecus coquereli	XP_012510288	------P-		-P---K--------------
	Aotus nancymaae	XP_012301126	-R------P-		-P---K--------------
	Sapajus apella	XP_032126177	-R------P-		-P---K--------------
	Theropithecus gelada	XP_025252974	-R------P-		-P---K--------------
	Callithrix jacchus	XP_008998363	-R------P-		-P---K--------------
	Trachypithecus francoisi	XP_033064624	-R------P-		-P---K--------------
	Hylobates moloch	XP_032000247	-R------P-		-P---K--------------
	Otolemur garnettii	XP_003786908	LC------P-		-P---K--------------
Other Mammalian Species	Canis lupus dingo	XP_025319873	------T-		-P---Q--------------
	Sus scrofa	XP_003130754	------T-		-P---K--------------
	Ursus maritimus	XP_040501222	------T-		-P---R--------------
	Camelus ferus	XP_032329899	------T-		-P---Q--------------
	Bos taurus	XP_024856648	--I---T-		-P---R-------------
	Equus caballus	XP_023488037	------T-		-P---Q--------------
	Vulpes vulpes	XP_025856657	------T-		-P---Q--------------
	Bison bison bison	XP_010858326	--I---T-		-P---R--------------

Supplementary figure S1: Multiple sequence alignment of a conserved segment of the protein junctional protein associated with coronary artery disease, showing a 1 amino acid insertion that is present in the grandorder Glires. This CSI was detected in all species within the Glires , and not detected in any other Euarchontoglires species. The dashes (-) within the alignment indicate the same amino acid as the top line.

			280		313
	Arvicanthis niloticus	XP_034366741	HVTDDKKTYKNPSLRAQG		QIRSPTKTHTPSPTSP
	Grammomys surdaster	XP_028619098	------------------		-V--------------
	Mus musculus	EDL41025	------------------		----------------
	Mus pahari	XP_021071103	-----E------------		-----I--------A-
	Mastomys coucha	XP_031214296	------------------		--Q----------I--
	Meriones unguiculatus	XP_021498495	-----Q------------		--Q----S-------A
	Rattus norvegicus	NP_446326	-------I----------		--------R------S
	Rattus rattus	XP_032740424	-------I----------		--------R----A-S
	Cricetulus griseus	XP_035297423	-----Q-I----------		-T------Y-------
	Mesocricetus auratus	XP_021080606	-----Q------------		-T---------N----
	Microtus ochrogaster	XP_005355164	-----Q--H---------		-T--------------
	Peromyscus leucopus	XP_028723520	-----Q------------		-A--------------
	Peromyscus maniculatus bairdii	XP_006972904	-----Q------------		-A--------------
	Onychomys torridus	XP_036045030	-----Q------------		-A--------------
Glires* *(31/31)	*Arvicola amphibius*	XP_038190686	-----Q--H---------		-T------P-------
	Jaculus jaculus	XP_004667990	-----Q------G---H-		-T---A----------
	Nannospalax galili	XP_008845283	-----Q------------		--P-------------
	Castor canadensis	XP_020021271	-----Q------------		-TQ-------------
	Dipodomys ordii	XP_012885880	-----Q-I----N-----		--P------S------
	Octodon degus	XP_023567824	-----Q------------		-T-----S-----V--
	Heterocephalus glaber	XP_021116256	-----Q------------		-T---S-S--------
	Cavia porcellus	XP_003468803	-----Q------------		-T-----S--------
	Chinchilla lanigera	XP_005403463	--R--Q------------		-T-----SL-------
	Fukomys damarensis	XP_010621211	----EQ------------		-T-----S--Q-----
	Marmota flaviventris	XP_027804010	-----Q------------		-V-----S--------
	Urocitellus parryii	XP_026257967	-----Q------------		-V-----S--------
	Marmota monax	KAF7465671	-----Q------------		-V-----S--------
	Ictidomys tridecemlineatus	XP_013215720	-----Q------------		-V-----S--------
	Marmota marmota marmota	XP_015349537	-----Q------------		-V-----S--------
	Oryctolagus cuniculus	XP_002720911	-----Q------------		-TK----S--------
	Ochotona princeps	XP_036349697	-----Q------------		-TP----S--------
Scandentia	*Tupaia chinensis*	XP_006165585	-----Q------------		-AP----S------P-
	Rhinopithecus bieti	XP_017729738	-----Q----------R-	G	-T-----S--------
	Rhinopithecus roxellana	XP_010351029	-----Q----------R-	G	-T-----S--------
	Gorilla gorilla gorilla	XP_004043357	-----Q------N-----	G	-TQ----S--------
	Pan paniscus	XP_034817398	-----Q------------	G	-TQ----S--------
	Homo sapiens	NP_001350462	-----Q------------	G	-TQ----S--------
	Saimiri boliviensis boliviensi	XP_039318624	-----Q------------	G	-T-----S--------
	Pan troglodytes	PNI62741	-----Q------------	G	-TQ----S--------
	Cercocebus atys	XP_011886788	-----Q------------	G	-T-----S--------
	Pongo abelii	Q5R5X8	-----Q------------	G	-T-----S--------
**Other *Euarchontoglires***	*Macaca fascicularis*	XP_007971801	-----Q------------	G	-T-----S--------
	Chlorocebus sabaeus	XP_007971800	-----Q------------	G	-T-----S--------
	Macaca mulatta	XP_028702567	-----Q------------	G	-T-----S--------
	Papio anubis	XP_021792715	-----Q------------	G	-T-----S--------
	Colobus angolensis palliatus	XP_011818136	-----Q------------	G	-T-----S--------
	Aotus nancymaae	XP_012332003	-----Q------------	G	-T-----S--------
	Callithrix jacchus	XP_035150720	-----Q------------	G	-T-----S--------
	Carlito syrichta	XP_008056861	-----Q------------	G	-TQ----S--------
	Theropithecus gelada	XP_025238895	-----Q------------	G	-T-----S--------
	Macaca nemestrina	XP_011740926	-----Q------------	G	-T-----S--------
	Sapajus apella	XP_032103543	-----Q------------	G	-T-----S--------
	Propithecus coquereli	XP_012498719	-----Q------------	G	-T-----SQ-------
	Microcebus murinus	XP_012609062	-----Q------G-----	G	-A-----S--------
	Camelus ferus	XP_032318433	-----Q---------T--	G	-TP----S-S---K--
	Gallus gallus	XP_040539641	--S--Q--H---------	P	PA-----S--------
	Loxodonta africana	XP_003416506	-----QM-H---------	G	-T-----SL-------
	Chaetura pelagica	XP_009999246	--S--Q--H---------	P	SVH----S-----N--
	Orcinus orca	XP_033290748	-----Q----------N-	G	-TP----S---G-K--
	Columba livia	XP_005512594	--S--Q--H--------C	P	PV-----S--------
	Dasypus novemcinctus	XP_004484841	--R--Q------------	G	-A-----S-S-G----
	Rousettus aegyptiacus	KAF6504196	-----Q------N-----	G	-TL-----Y--G-K--

Supplementary figure S2: Multiple sequence alignment of a conserved segment of the protein adenylyl cyclase-associated protein 2, showing a 1 amino acid deletion that is specifically shared by members of the orders Rodentia, Lagomorpha and Scandentia. Homologs of this protein are not found in most mammalian species outside of Euarchontoglires.

Group	Species	Accession	696		747
**Rodentia (29/29)**	*Rattus rattus*	XP_032741230	WKTCPVCNEL	FPSNVYQVHMEVAHKQSEVQLCQVCNEP	FPANVYQVHMEVAH
	Mastomys coucha	XP_031221977	----------	------------------A--------L	--------------
	Mus musculus	XP_036017036	----------	------------------A--------L	--------------
	Arvicanthis niloticus	XP_034374133	----------	------------------A--------L	--------------
	Mus caroli	XP_021006671	----------	------------------A--------L	--------------
	Mus pahari	XP_021070532	----------	------------------A--------L	--------------
	Grammomys surdaster	XP_028624772	----------	------------------A--------L	---------V----
	Meriones unguiculatus	XP_021495735	----------	--A---------------P---P----L	--------------
	Arvicola amphibius	XP_038186908	----------	------------------S--------L	-----------M--
	Cricetulus griseus	XP_003501883	----------	------------------S--------L	------------V-
	Mesocricetus auratus	XP_012978917	----------	------------------S--------L	------------V-
	Onychomys torridus	XP_036060242	----------	------------------S---RI---L	--------------
	Peromyscus leucopus	XP_037053108	----------	------------------S---R----L	-----------M--
	Neotoma lepida	OBS68149	----------	------------------S---R----L	------------V-
	Peromyscus maniculatus bairdii	XP_006973926	----------	------------------S---R----R	-----------M--
	Microtus ochrogaster	XP_026639366	----------	------------------S-R-R----L	-----------M--
	Nannospalax galili	XP_029410016	----------	------------------S--------L	--S-----------
	Dipodomys ordii	XP_012881597	----------	---------------HT-T---R----L	--S-----------
	Castor canadensis	XP_020038592	----R-----	-----------------KS---RD---L	--S-----------
	Fukomys damarensis	XP_010611647	----R-----	---------------N--SK--R----L	--S-----------
	Heterocephalus glaber	XP_004861954	----R-----	---------------N--SK--R----L	--S-----------
	Chinchilla lanigera	XP_005373183	----R-----	---------------N--SK--L----L	--S-----------
	Octodon degus	XP_023555830	----R-----	---------------N--SK--R----L	L-S-----------
	Cavia porcellus	XP_003463361	----R-----	---------------N--SK--R----L	--S---E-------
	Marmota monax	VTJ87490	----R-----	---------------H--SK--R----L	--S------V----
	Urocitellus parryii	XP_026236897	----R-----	---------------H--SK--R----L	--S------V----
	Marmota marmota marmota	XP_015355100	----R-----	---------------H--SK--R----L	--S------V----
	Ictidomys tridecemlineatus	XP_005334418	----R-----	---------------H--SK--R----L	--S------V----
	Marmota flaviventris	XP_027779775	----R-----	---------------H--SK--R----L	--S------V----
**Lagomorpha**	*Oryctolagus cuniculus*	XP_008251129	----------		--S-----------
**Other Euarchontoglires**	*Tupaia chinensis*	XP_014438565	----------		--S-----------
	Gorilla gorilla gorilla	XP_030859624	----------		--S-----------
	Pan troglodytes	XP_016789465	----------		--S-----------
	Trachypithecus francoisi	XP_033034340	----------		--S-----------
	Aotus nancymaae	XP_012328915	----------		--S-----------
	Sapajus apella	XP_032149700	----------		--S-----------
	Homo sapiens	XP_011524185	----------		--S-----------
	Pongo abelii	XP_002828389	----------		--S-----------
	Papio anubis	XP_003914573	----------		--S-----------
	Cercocebus atys	XP_011938981	----------		--S-----------
	Macaca mulatta	XP_014977735	----------		--S-----------
	Pan paniscus	XP_034799320	----------		--S-----------
	Rhinopithecus bieti	XP_017718937	----------		--S-----------
	Saimiri boliviensis boliviensi	XP_010335014	----------		--S-----------
	Macaca fascicularis	XP_005586412	----------		--S-----------
	Macaca nemestrina	XP_011752270	----------		--S-----------
	Rhinopithecus roxellana	XP_030781939	----------		--S-----------
	Carlito syrichta	XP_021571104	----------		--S-------Q---
	Otolemur garnettii	XP_012659774	----------		--S-----------
	Callithrix jacchus	XP_035127320	----------		--S-----------
	Microcebus murinus	XP_012644383	----------		--S-----------
	Propithecus coquereli	XP_012513269	----------		--S-----------
	Piliocolobus tephrosceles	XP_023080573	----------		--S-----------
	Nomascus leucogenys	XP_030666087	----------		--S-----------
	Theropithecus gelada	XP_025221396	----------		--S-----------
	Chlorocebus sabaeus	XP_008011881	----------		--S-----------
	Hylobates moloch	XP_032007895	----------		--S-----------
	Colobus angolensis palliatus	XP_011789764	----------		--S-----------
**Other Mammalian species**	Camelus ferus	XP_032326567	----------		--S-----------
	Equus caballus	XP_023503813	----------		--S-----------
	Felis catus	XP_019671048	----------		--S-----------
	Sus scrofa	XP_013849425	----------		--S-----------
	Equus caballus	XP_005613058	----------		--S----------
	Bison bison bison	XP_010853387	----------		--S-----------
	Ovis aries	KAG5195518	----------		--S-----------
	Ursus arctos horribilis	XP_026352335	----------		--S-----------
	Capra hircus	XP_017895200	----------		--S-----------
	Molossus molossus	XP_036101415	----------		--S-----------
	Dasypus novemcinctus	XP_023446870	----------		--S-----------

Supplementary figure S3: Multiple sequence alignment of a conserved segment of the protein activity-dependent neuroprotector homeobox protein 2, showing a 28 amino acid insertion that is present in the order Rodentia. There are two homologs of this protein in Rodentia species and this insert is only found in protein 2..

Group	Species	Accession	153		178
***Lagomorpha* (2/2)**	*Oryctolagus cuniculus*	XP_017203176	QLKTQVARLQA		DMRGIVSGLQLK
	Ochotona princeps	XP_036351121	---M-------		-LQS---E----
***Myomorpha* (0/14)**	*Meriones unguiculatus*	XP_021499278	------M----	EKA	-LL----E----
	Mus musculus	NP_001343416	H--I--M--R-	EKA	-LL----E----
	*Rattus norvegicus**	XP_017455943	---R--R----	EKA	-LL----E----
	Rattus rattus	XP_032740075	---I--R----	EKA	-LL--I-E----
	Mus caroli	XP_021011665	H--I--M--R-	EKA	-LL----E----
	Grammomys surdaster	XP_028620724	H--I--M----	EKA	-LLS---E----
	Mastomys coucha	XP_031214207	H--I--M----	EKA	-LL----E----
	Mus pahari	XP_021071594	H--I--M----	EKA	-LL----E----
	Peromyscus maniculatus bairdii	XP_006974721	------M----	EKA	-LL----E----
	Peromyscus leucopus	XP_028747693	------M----	EKA	-LL----E----
	Arvicola amphibius	XP_038189704	------M----	EKA	-LL----E----
	Microtus ochrogaster	XP_005354913	---A--M----	EKA	-LL----E----
	Cricetulus griseus	XP_003496453	---I--M--K-	EKA	-LL----E----
	Nannospalax galili	XP_008846755	------V--R-	EKA	-LL--I-E----
***Castorimorpha* (0/2)**	*Dipodomys ordii*	XP_012870164	-----------	EKA	-LL----E----
	Castor canadensis	XP_020024814	-----------	EKA	-LL----E----
***Hystricomorpha* (0/5)**	*Chinchilla lanigera*	XP_013376900	---A------D	EKA	-LL----E----
	Octodon degus	XP_023563825	---V------N	EKA	-LL----E----
	Heterocephalus glaber	XP_012933981	---AK-----N	EKA	-LL----E----
	Cavia porcellus	XP_013006729	---A---K--Y	EKA	-LL--I-E----
	Fukomys damarensis	XP_010619596	--RVK-----N	EKA	-LL----E----
***Sciuromorpha* (0/5)**	*Marmota monax*	VTJ91467	-----------	EKA	-LL----E----
	Marmota marmota marmota	XP_015339015	-----------	EKA	-LL----E----
	Marmota flaviventris	XP_027784392	-----------	EKA	-LL----E----
	Ictidomys tridecemlineatus	KAG3257380	-----------	EKA	-LL----E----
	Urocitellus parryii	XP_026236800	-----------	EKA	-LL----E----
**Other *Euarchontoglires***	*Tupaia chinensis*	XP_006150066	------T----	EKA	-LL----E----
	Pan troglodytes	PNI40151	--R---V----	EKA	-LL----E----
	Colobus angolensis palliatus	XP_011789621	--R--------	EKA	-LL----E----
	Cercocebus atys	XP_011889414	--R--------	EKA	-LL----E----
	Chlorocebus sabaeus	XP_008000470	--R--------	EKA	-LL----E----
	Rhinopithecus bieti	XP_017730173	--R--------	EKA	-LL----E----
	Rhinopithecus roxellana	XP_010364245	--R--------	EKA	-LL----E----
	Otolemur garnettii	XP_012658796	-----------	EKA	-LL----E----
	Macaca mulatta	EHH18890	--R---T----	EKA	-LL----E----
	Macaca fascicularis	EHH64565	--R---T----	EKA	-LL----E----
	Papio anubis	XP_009212247	--R---T----	EKA	-LL----E----
	Macaca nemestrina	XP_011754721	--R---T----	EKA	-LL----E----
	Pongo abelii	XP_024108832	--R---V----	EKA	-LL----E----
	Pan paniscus	XP_034785847	--R---V----	EKA	-LL----E----
	Homo sapiens	EAW86307	--R---V----	EKA	-LL----E----
	Gorilla gorilla gorilla	XP_018890639	--R---V----	EKA	-LL----E----
	Saimiri boliviensis boliviensi	XP_039334802	--R---V----	EKA	-LL----E----
	Sapajus apella	XP_032099931	--R---V----	EKA	-LL----E----
	Nomascus leucogenys	XP_030675133	--R---V----	EKA	-LL----E----
	Callithrix jacchus	XP_002750089	--R---V----	EKA	-LL----E----
	Carlito syrichta	XP_021574360	--R--------	EKA	-LL----E----
	Aotus nancymaae	XP_012311430	--R---V----	EKA	-LL----E----
	Propithecus coquereli	XP_012516390	---M-------	EKA	-LL----E----
	Microcebus murinus	XP_012621157	---M-------	EKA	-LL----E----

*Has EQEVEHLKNQV after position 158

Supplementary figure S4: Multiple sequence alignment of a conserved segment of the protein optineurin, showing a 3 amino acid deletion that is present in the order Lagomorpha. This CSI was detected in all species within Lagomorpha, and not detected in any other members of the Euarchontoglires in the top 250 BLASTp returns. The dashes (-) within the alignment indicate the same amino acid as the top line. The second column indicates accession numbers for each of the sequences and the position of the partial sequence is shown by the numbers at the top.

Group	Species	Accession	192		227
**_Lagomorpha (2/2)_**	*Ochotona princeps*	504168620	EKLRKEKEELEKADMD	D	LRHFDYPQEILTGELARIIY
	Oryctolagus cuniculus	655869950	-------Q--------	-	--NL-------K--------
**Other _Euarchontoglires_**	*Mus musculus*	74146777	-------Q-F---A--		MGD--H-E---K--------
	Rattus norvegicus	109491454	-------Q-F---A--		MGD--H-E---K------V-
	Grammomys surdaster	XP_028615051	-------Q-F---A--		VGN--H-E---K--------
	Meriones unguiculatus	XP_021486645	-------Q-F---A--		MGN--H-E-V-K--------
	Mastomys coucha	XP_031208880	-------Q-F---A--		MGS--H-E---K--------
	Arvicanthis niloticus	XP_034362918	-------Q-F---A--		MGD--H-E---K--------
	Mus caroli	XP_021031689	-------Q-F---A--		MGD--H-E---K--------
	Mus pahari	XP_021068357	-------Q-F---A--		MGD--H-E---K--------
	Rattus rattus	XP_032768742	-------Q-F---A--		MGD--H-E---K------V-
	Cricetulus griseus	537140452	-------Q-F---A--		MGN--H-E---K--------
	Mesocricetus auratus	524954120	-------Q-F---A--		MGN--H-E---K--------
	Microtus ochrogaster	532011371	---------F---A-E		MGS--H-E---E--------
	Peromyscus maniculatus bairdii	XP_006977563	-------Q-FD--A--		MGN--H-E---K--------
	Peromyscus leucopus	XP_028722775	-------Q-FD--A--		MGN--H-E---K--------
	Jaculus jaculus	847030551	-------Q-F------		MGN--HSE---N--F-----
	Nannospalax galili	674093216	-------Q-F---S--		MGN--H-E---K--------
	Castor canadensis	XP_020030064	-------K-F---A--		VGE--H-E---M--------
	Dipodomys ordii	XP_012871742	-------Q-F---A--		MGD--Q-E---R--------
	Heterocephalus glaber	351699226	-------Q-F------		MGD----E---K-Q------
	Chinchilla lanigera	533179278	---------F------		MGD--H-E---K--------
	Octodon degus	820973315	-------Q-F------		MGD--H-E---K--------
	Fukomys damarensis	731256080	-------Q-F------		MGD--H-E---K--------
	Cavia porcellus	348567777	-------Q-F------		MGD-EH-E---K------V-
	Ictidomys tridecemlineatus	532084246	-------Q-F------		MGS--HSE---N--------
	Marmota monax	KAF7475083	-------Q-F------		MGS--HSE---N--------
	Marmota marmota marmota	XP_015356648	-------Q-F------		MGS--HSE---N--------
	Tupaia chinensis	562851389	-------Q-F-R---E		METS---E---K--------
	Colobus angolensis palliatus	795164392	---------F---S--		VE-P--SE---K--------
	Mandrillus leucophaeus	795203827	---------F---S--		VE-P--SE---K--------
	Chlorocebus sabaeus	635089038	---------F---S--		VE-P--SE---K--------
	Macaca fascicularis	544498167	---------F---S--		VE-P--SE---K--------
	Cercocebus atys	795290957	---------F---S--		VE-P--SE---K--------
	Macaca mulatta	387763411	---------F---S--		VE-P--SE---K--------
	Rhinopithecus roxellana	724872308	---------F---S--		VE-P--SE---K------V-
	Galeopterus variegatus	667310708	-------Q-F-----G		VGNL---E-L-K--------
	Microcebus murinus	829721352	-------Q-F---N--		VENL--SE---Q------V-
	Propithecus coquereli	826305185	-------Q-F---N--		VENL--SE---Q------V-
	Callithrix jacchus	675659650	---------F---S--		MENP--SE---K--------
	Aotus nancymaae	817269299	---------F---S--		MENP--SE---K--------
	Nomascus leucogenys	332262415	---------F---S--		VENP--SE---K--------
	Homo sapiens	767995265	---------F---S--		VENP--SE---K----W---
	Gorilla gorilla gorilla	426348800	---------F---S--		VENP--SE---K----W---
	Pan troglodytes	694967702	---------F---S--		VENP--SE---K----W---
	Pan paniscus	397481591	---------F---S--		VENP--SE---K----W---
	Macaca nemestrina	XP_011745724	---------F---S--		VE-P--SE---K--------
	Papio anubis	XP_031513315	---------F---S--		VE-P--SE---K--------
	Rhinopithecus bieti	XP_017746424	---------F---S--		VE-P--SE---K------V-
	Carlito syrichta	XP_008046719	-------Q-F---S--		VENL--SE---K--------
	Pongo abelii	XP_024091317	---------F---S--		VENP--SE---K--------
	Saimiri boliviensis boliviensis	XP_039332329	--M------F---S--		MENP--SE---K--------

Supplementary figure S5: Multiple sequence alignment of a conserved segment of the protein U3 small nucleolar RNA-associated protein 6 homolog, showing a 1 amino acid insertion that is present in the order Lagomorpha. This CSI was detected in all species within Lagomorpha, and not detected in any other members of the Euarchontoglires in the top 250 BLASTp returns. The dashes (-) within the alignment indicate the same amino acid as the top line. The second column indicates accession numbers for each of the sequences and the position of the partial sequence is shown by the numbers at the top.

Group	Species	Accession	1893		1927
**Lagomorpha (2/2)**	*Oryctolagus cuniculus*	XP_017196585	VRPVNPSNTNSSPKHNNTS	N	RLPNQNGTVLPSESPGL
	Ochotona princeps	XP_004586700	------G--------TP--	-	-----------T---V-
**Other Euarchontoglires (1/51)**	*Meriones unguiculatus*	XP_021507359	------G-A------SSNT	A	-------P---------
	Arvicanthis niloticus	XP_034374441	------G-----------A		-------P-----A---
	Rattus norvegicus	XP_038952457	------G--S--------A		-------P---------
	Mus caroli	XP_021006711	------G--S--------A		-------P---------
	Mus pahari	XP_029403080	------G--S--------A		-------P---------
	Mus musculus	NP_780584	------G--S--------A		-------P---------
	Rattus rattus	XP_032741365	------G--S--------A		-------P---------
	Grammomys surdaster	XP_028618666	----T-G--S-------SA		-------P---------
	Peromyscus leucopus	XP_028742570	------G------------		-------P---------
	Peromyscus maniculatus bairdii	XP_015864764	------G------------		-------P---------
	Microtus ochrogaster	XP_013204969	------G-S---------A		-------P---------
	Cricetulus griseus	ERE85571	------G-A---------A		-------P---------
	Jaculus jaculus	XP_004652481	------G-A---------T		-----------------
	Nannospalax galili	XP_029420581	------G-A--------AT		-----------------
	Castor canadensis	XP_020012707	------G-----------T		---S-----IQ---S--
	Dipodomys ordii	XP_012875758	------G--SN-----T-T		----------S------
	Heterocephalus glaber	XP_004841932	------G-----------T		-----------------
	Fukomys damarensis	XP_033623060	------G-----------T		-----------------
	Cavia porcellus	XP_023416275	------G-----------T		----------S------
	Octodon degus	XP_023559156	------G---N-------T		-P---------------
	Marmota flaviventris	XP_027783755	------G-----------T		-----------------
	Marmota monax	KAF7461645	------G-----------T		-----------------
	Marmota marmota marmota	XP_015352376	------G-----------T		-----------------
	Urocitellus parryii	XP_026240272	------G-----------T		-----------------
	Ictidomys tridecemlineatus	XP_040129665	------G-----------T		-----------------
	Tupaia chinensis	XP_014446274	------G--S---------		----------S---A--
	Sapajus apella	XP_032106327	------G------------		--------------A--
	Papio anubis	XP_021795437	------G------------		--------------A--
	Macaca mulatta	XP_028705586	------G------------		--------------A--
	Saimiri boliviensis boliviensi	XP_039318281	------G------------		--------------A--
	Rhinopithecus roxellana	XP_030783560	------G------------		--------------A--
	Gorilla gorilla gorilla	XP_018882500	------G------------		--------------A--
	Microcebus murinus	XP_012604333	------G------------		--------------A--
	Pan troglodytes	XP_016809433	------G------------		--------------A--
	Hylobates moloch	XP_032013979	------G------------		--------------A--
	Pan paniscus	XP_008952804	------G------------		--------------A--
	Chlorocebus sabaeus	XP_008012870	------G------------		--------------A--
	Cercocebus atys	XP_011945890	------G------------		--------------A--
	Macaca fascicularis	XP_005558031	------G------------		--------------A--
	Macaca nemestrina	XP_011714638	------G------------		--------------A--
	Aotus nancymaae	XP_012325257	------G------------		--------------A--
	Trachypithecus francoisi	XP_033036849	------G------------		--------------A--
	Homo sapiens	EAW62054	------G------------		--------------A--
	Pongo abelii	XP_024102848	------G------------		--------------T--
	Propithecus coquereli	XP_012516813	------G------------		--------------A--
	Carlito syrichta	XP_008054321	------G------------		---S----------A--
	Rhinpithecus bieti	XP_017729371	------G------------		--------------A--
	Pan troglodytes	XP_016809426	------G------------		--------------A--
	Callithrix jacchus	XP_008984084	------G--------- --		--------------A--
	Otolemur garnettii	XP_023371638	------G--SN-------G		--------------A--
	Galeopterus variegatus	XP_008572546	------G------------		----------S---AAS

Supplementary figure S6: Multiple sequence alignment of a conserved segment of the protein ankyrin repeat and KH domain-containing protein 1, showing a 1 amino acid insertion that is present in the order Lagomorpha. This CSI was detected in all species within Lagomorpha, and only 1 was detected in the other members of the Euarchontoglires in the top 250 BLASTp returns. The dashes (-) within the alignment indicate the same amino acid as the top line. The second column indicates accession numbers for each of the sequences and the position of the partial sequence is shown by the numbers at the top.

	Species	Accession	553		586
***Lagomorpha* (2/2)**	*Oryctolagus cuniculus*	XP_008257603	DGESKPRPALYSLQD		LEAEDCEKMSNMGTLN
	Ochotona princeps	XP_004597923	-A-G-A--P------		--V-------------
**Other *Euarchontoglires***	*Mus caroli*	XP_021038983	--------S-----N	FEE	I---------------
	Rattus norvegicus	AAQ03034	--------S-----N	FEE	I---------------
	Mus musculus	NP_001028389	--------S-----N	FEE	I---------------
	Grammomys surdaster	XP_028607991	--------S-----N	FEE	I---------------
	Meriones unguiculatus	XP_021520448	--------S-----N	FEE	I---------------
	Mus pahari	XP_029403716	--------S-----N	FEE	I---------------
	Arvicanthis niloticus	XP_034372453	---T----S-----N	FEE	I---------------
	Rattus rattus	XP_032741287	--------S-----N	FEE	I-T-------------
	Mastomys coucha	XP_031209373	--------S-----N	FEE	I-P-------------
	Microtus ochrogaster	XP_005354359	--------S-----N	FEE	V---------------
	Cricetulus griseus	EGW07824	--------S-----N	FEE	M--D------------
	Peromyscus maniculatus bairdii	XP_006974380	--------S-----N	FEE	M--D------------
	Peromyscus leucopus	XP_028725118	--------S-----N	FEE	M--D------------
	Jaculus jaculus	XP_004662228	--------S-----N	FEE	I--DE-----------
	Nannospalax galili	XP_008843081	-A-N-------P--T	FEE	M---G---T-------
	Dipodomys ordii	XP_012874191	---G----S-----N	FEE	M--DE-----------
	Castor canadensis	XP_020009489	---G----S-----N	FEE	M--DE-----------
	Octodon degus	XP_004638637	--------S-----N	FEE	M---------------
	Cavia porcellus	XP_003475903	--------S-----N	FEE	I---------------
	Fukomys damarensis	XP_010639225	--------S-----N	FEE	M---------------
	Chinchilla lanigera	XP_005406635	--------S-----N	FEE	M-T-------------
	Heterocephalus glaber	XP_004863228	-A------S-----N	FEE	I---------------
	Ictidomys tridecemlineatus	XP_005324903	--------S-----N	FEE	I---------------
	Marmota monax	KAF7475655	--------S-----N	FEE	M---------------
	Marmota marmota marmota	XP_015348722	--------S-----N	FEE	M---------------
	Urocitellus parryii	XP_026257229	--------S-----N	FEE	M---------------
	Tupaia chinensis	ELV10691	--------S-----N	FEE	M-T-------------
	Propithecus coquereli	XP_012493913	--------S-----N	FEE	M---------------
	Otolemur garnettii	XP_003793593	--------S-----N	FEE	--T-------------
	Carlito syrichta	XP_008062048	--------S-----N	FEE	M-T-------------
	Microcebus murinus	XP_012599325	--------S-----N	FEE	M-T-------------
	Homo sapiens	BAB71116	---N----S-----N	FEE	M-T-------------
	Rhinopithecus bieti	XP_017710784	--------S-----N	FEE	M-T-------------
	Gorilla gorilla gorilla	XP_004053022	--------S-----N	FEE	M-T-------------
	Chlorocebus sabaeus	XP_037853171	--------S-----N	FEE	M-T-------------
	Rhinopithecus roxellana	XP_010380681	--------S-----N	FEE	M-T-------------
	Aotus nancymaae	XP_012321061	--------S-----N	FEE	M-T-------------
	Pongo abelii	XP_002823161	--------S-----N	FEE	M-T-------------
	Callithrix jacchus	XP_035111657	--------S-----N	FEE	M-T-------------
	Saimiri boliviensis boliviensi	XP_003926709	--------S-----N	FEE	M-T-------------
	Macaca mulatta	XP_015006974	---T----S-----N	FEE	M-T-------------
	Papio anubis	XP_009178798	---T----S-----N	FEE	M-T-------------
	Macaca nemestrina	XP_011720199	---T----S-----N	FEE	M-T-------------
	Macaca fascicularis	XP_005570656	---T----S-----N	FEE	M-T-------------
	Cercocebus atys	XP_011884793	---T----S-----N	FEE	M-T-------------
	Pan troglodytes	NP_001267464	---N----S-----N	FEE	M-T-------------
	Galeopterus variegatus	XP_008568749	---N----S-----N	FEE	M---------------
	Colobus angolensis palliatus	XP_011791479	--------S-----N	FEE	M-T-------------
	Sapajus apella	XP_032104370	--------S-----N	FEE	M-T-------------
	Pan paniscus	XP_008949308	---N----S-----N	FEE	M-T-------------

Supplementary figure S7: Multiple sequence alignment of a conserved segment of the protein prickle-like protein 1, showing a 3 amino acid deletion that is present in the order Lagomorpha. This CSI was detected in all species within Lagomorpha, and not detected in any other members of the Euarchontoglires in the top 250 BLASTp returns. The dashes (-) within the alignment indicate the same amino acid as the top line. The second column indicates accession numbers for each of the sequences and the position of the partial sequence is shown by the numbers at the top.

Group	Species	Accession	176		215
**Myomorpha (18/18)**	*Rattus norvegicus*	33149327	WVLSFILSTPQYFIFSVIE	IE	VNNGTKTQDCWATFIQPWG
	Mus musculus	74146437	-G---V--I----------	F-	------A--------P---
	Rattus rattus	XP_032746701	-------------------	--	-------------------
	Mastomys coucha	XP_031205492	-----V--I--F-------	--	------A------------
	Meriones unguiculatus	XP_021519787	-A---V----------MV-	--	-S---TA------------
	Mus pahari	XP_021061242	-G---V--I----------	F-	------A--------P---
	Mus caroli	XP_021029963	-G---V--I----------	F-	------A--------P---
	Gramomys surdaster	XP_028642378	----------------KL-	L-	------VLE--G-------
	*Arvicanthis niloticus**	XP_034343181	-----V--I-------M--	--	------ALE--G-------
	Peromyscus leucopus	296492384	-----L----------M--	--	-------------------
	Microtus ochrogaster	54036618	-----L----------M--	--	-------------------
	Cricetulus griseus	354477250	-----V----------M--	--	-------------------
	Mesocricetus auratus	XP_040610728	-----V----------M--	--	-------------------
	Arvicola amphibius	XP_038170773	-----L----------M--	--	-------------------
	Peromyscus maniculatus bairdii	XP_006973397	-----L----------M--	--	-------------------
	Onychomys torridus	XP_036025879	-----L----------M--	--	-------------------
	Nannospalax galili	674074172	-----V----------M--	--	------A------------
	Jaculus jaculus	507532064	-----V----------L--	V-	---V--A------------
**Castorimorpha (0/2)**	*Castor canadensis*	XP_020039638	-----V----------M--		---V--A------------
	Dipodomys ordii	XP_012878631	-----V----------M--		---V--A------------
**Hystricomorpha (0/5)**	*Chinchilla lanigera*	533167903	-A---V----------M--		-D-V--AH-----------
	Fukomys damarensis	731260842	-----V----------M--		-D-V--A------------
	Heterocephalus glaber	512810391	-----V----------M--		-D-V--AR-----------
	Cavia porcellus	348580465	-----V----------M--		-D-V--A------------
	Octodon degus	507690158	-A---A----------M--		-D-V--A------------
**Sciuromorpha (0/5)**	*Ictidomys tridecemlineatus*	532086040	-----V----------M--		---V--A------------
	Urocitellus parryii	XP_026248771	-----V----------M--		---V--A------------
	Marmota flaviventris	XP_027785023	-----V----------M--		---V--A------------
	Marmota marmota marmota	XP_015338549	-----V----------M--		---V--A------------
	Marmota monax	KAF7463928	-----V----------M--		---V--A------------
**Other Euarchontoglires**	*Oryctolagus cuniculus*	291389479	-----V----------M--		---V--A------------
	Ochotona princeps	504141204	----LV----------M--		---V--A------------
	Tupaia chinensis	562850944	-----V----------MV-		---E---L-----------
	Gorilla gorilla gorilla	426373296	-----V-------V--M--		---V--AR-----------
	Saimiri boliviensis boliviensi	403269041	-----V-------V-----		---V--AR-----------
	Papio anubis	402886694	-----V-------V--M--		---V--AR-----------
	Cercocebus atys	795422264	-----V-------V--M--		---V--AR-----------
	Macaca fascicularis	309401194	-----V-------V--M--		---V--AR-----------
	Macaca nemestrina	795599770	-----V-------V--M--		---V--AR-----------
	Chlorocebus sabaeus	635066559	-----V-------V--M--		---V--AR-----------
	Pan paniscus	397508863	-----V-------V--M--		---V--AR-----------
	Pan troglodytes	410046406	-----V-------V--M--		---V--AR-----------
	Pongo abelii	297692335	-----V-------V--M--		---V--AR-----------
	Macaca mulatta	297262857	-----V-------V--M--		---V--AR-----------
	Homo sapiens	74315883	-----V-------V--M--		---V--AR-----------
	Aotus nancymaae	817350400	-----V-------V--M--		---V--AR-----------
	Colobus angolensis palliatus	795334470	-----V-------V--M--		---V--AR-----------
	Callithrix jacchus	675680846	-----L-------V--M--		---V--AR-----------
	Sapajus apella	655349171	-----V-------V--M--		---V--AR-----------
	Rhinopithecus roxellana	724796031	-----V--M----V--M--		---V--AR-----------
	Otolemur garnettii	395852123	-----L-------V--I--		-D-V--AL-----------
	Microcebus murinus	829853412	----LV--A----V--M--		---V--AR-----------
	Galeopterus variegatus	667289048	-----L-------V--M--		-S-V--AR-------E---
	Theropithecus gelada	XP_025258628	-----V-------V--M--		---V--AR-----------
	Hylobates moloch	XP_032028020	-----V-------V--M--		---V--AR-----------
	Nomascus leucogenys	XP_003252777	-----V-------V--M--		---V--AR-----------
	Propithecus coquereli	XP_012517030	----LV-------V--M--		---V--AR-----------
	Piliocolobus tephrosceles	XP_023080883	-----V--M----V--M--		---V--AR-----------
	Trachypithecus francoisi	XP_033077308	-----V--M----V--M--		---V--AR-----------
	Rhinopithecus bieti	XP_017741186	-----V--M----V--M--		---V--AR-----------
	Carlito syrichta	XP_008072317	-L--LV--A---LV--MV-		---V--AR------V----

*Extra N amino acid after site 199

Supplementary figure S8: Multiple sequence alignment of a conserved segment of the protein vasopressin V1a receptor, showing a 2 amino acid insertion that is present in the suborder Myomorpha. This CSI was detected in all species within Myomorpha, and not detected in any other members of the Euarchontoglires in the top 250 BLASTp returns. The dashes (-) within the alignment indicate the same amino acid as the top line. The second column indicates accession numbers for each of the sequences and the position of the partial sequence is shown by the numbers at the top.

			584		619
***Myomorpha* (19/19)**	*Arvicanthis niloticus*	XP_034373275	LTLEVPQTPEVLRSPGA		PSPGLPESCPYSSPQEKSM
	Mastomys coucha	XP_031211290	-----------------		------------------T
	Mus caroli	XP_021039134	-----------------		-------------------
	Mus musculus	XP_006521185	-----------------		------------G------
	Grammomys surdaster	XP_028644121	-----------------		-------------------
	Mus pahari	XP_021072728	---------------R-		------------G------
	Meriones unguiculatus	XP_021490628	-------P---------		-----------G-----GL
	Rattus norvegicus	NP_001258298	--------H--------		------GP----------V
	Rattus rattus	XP_032742247	--------H--------		------GP----------V
	Mesocricetus auratus	XP_005067280	-------A---------		-----S-----G---D--L
	Microtus ochrogaster	XP_026638406	---------G-------		-----S-P---G-----GL
	Peromyscus maniculatus bairdii	XP_015848635	-------A---------		-----S-----G------L
	Peromyscus leucopus	XP_028730441	-------A---------		-----S-----G------L
	Neotoma lepida	OBS79536	-------A--I------		-----S-----G------L
	Onychomys torridus	XP_036063521	-------A---------		---D-S-----G---D--L
	Cricetulus griseus	ERE81205	-------A---------		S----S-----G---D--L
	Arvicola amphibius	XP_038198837	-------A---------		-----S-P---G-----GL
	Nannospalax galili	XP_008846975	-A-----G---------		-G-C-------G------L
	Jaculus jaculus	XP_004649871	--------S--------		---C-------GGA----L
**Other *Euarchontoglires* (1/43)**	*Dipodomys ordii*	XP_012874148	-------A--------V		S--C-----S-G-----NL
	Castor canadensis	XP_020025053	-------AS-------V	L	---C-----S-G------L
	Cavia porcellus	XP_005006536	-A-----AS-G----R-	P	---C-S-----GT-----S
	Fukomys damarensis	XP_033619435	---A--------V	P	---C-S----CG------S
	Octodon degus	XP_023578490	-A-----A--G----RV	S	---C-S--F-FG------S
	Heterocephalus glaber	XP_004863069	-S-----A-------RV	P	-T-C-S-----GA----GS
	Chinchilla lanigera	XP_005411906	-A-----A--G----RV	S	---C-S-----G------S
	Urocitellus parryii	XP_026262816	-------A--------V	P	---C-------G------L
	Marmota marmota marmota	XP_015348814	-------A--------V	P	---C-------G------L
	Marmota flaviventris	XP_027805547	-------A--------V	P	---C-------G------L
	Marmota monax	VTJ72549	-------A--G-----V	P	---C-------G------L
	Ictidomys tridecemlineatus	XP_005324984	-------A--------V	P	---C-------G------L
	Ochotona princeps	XP_004599040	-------AS-G-----V	P	S------P---G------L
	Oryctolagus cuniculus	XP_017197329	-------A--G-----G	P	---C-------G-T---NL
	Tupaia chinensis	ELV10863	-------A--G-----V	P	---C-------GI-----S
	Piliocolobus tephrosceles	XP_023066951	-------A--------V	P	---C----Y--G------L
	Trachypithecus francoisi	XP_033078036	-------A--------V	P	---C----Y--G------L
	Colobus angolensis palliatus	XP_011791548	-------A--------V	P	---C----Y--G------L
	Homo sapiens	EAW58094	-------A--------V	P	---C----Y--G------L
	Gorilla gorilla gorilla	XP_018893738	-------A--------V	P	---C----Y--G------L
	Pan paniscus	XP_008949438	-------A--------V	P	---C----Y--G------L
	Pan troglodytes	PNI33351	-------A--------V	P	---C----Y--G------L
	Cercocebus atys	XP_011903746	-------A--------V	P	---C----Y--G------L
	Macaca fascicularis	XP_005570844	-------A--------V	P	---C----Y--G------L
	Macaca mulatta	XP_015007167	-------A--------V	P	---C----Y--G------L
	Macaca nemestrina	XP_011758467	--V----A--------V	P	---C----Y--G------L
	Hylobates moloch	XP_031999793	-------A--------V	P	---CF---Y--G------L
	Chlorocebus sabaeus	XP_008001367	-------A--------V	P	---C-A--Y--G------L
	Papio anubis	XP_003906395	-------A--------V	P	---C----YR-G------L
	Sapajus apella	XP_032150015	-------A--I-----V	P	S--C----Y--G------L
	Saimiri boliviensis boliviensis	XP_003939192	-------A--I-----V	P	---C----Y--G------L
	Carlito syrichta	XP_008060457	------PA--L-----V	P	---C-------G------L
	Callithrix jacchus	XP_035112747	-------A--I-----V	P	---C----Y--G------L
	Aotus nancymaae	XP_012296837	-------A-KI-----V	P	---C----Y--G------L
	Pongo abelii	PNJ25779	-------A--------V	P	---CPA--Y--G------L
	Nomascus leucogenys	XP_030672466	-------A--------V	P	---CVL--Y--GN-----L
	Microcebus murinus	XP_012617217	-------A--A-----V	P	---C---- --Y-----L
	Rhinopithecus bieti	XP_017750218	-------A--------V	P	---C----Y--G------L
	Rhinopithecus roxellana	XP_010383678	-------A--------V	P	---C----Y--G------L
	Theropithecus gelada	XP_025258989	-------A--------V	P	---C----Y--G------L
	Otolemur garnettii	XP_012662949	-------A--------V	P	---C----Y--G-----GL
	Galeopterus variegatus	XP_008581132	-------A--------V	P	---C-----S-V------L
	Propithecus coquereli	XP_012507809	---V---P--A-----V	P	---C---- --Y-----L

Supplementary figure S9: Multiple sequence alignment of a conserved segment of the protein nck-associated protein 5-like isoform X1 showing a 1 amino acid deletion that is present in the suborder Myomorpha. This CSI was detected in all species within Myomorpha, and only detected in 1 other member of the Euarchontoglires in the top 250 BLASTp returns. The dashes (-) within the alignment indicate the same amino acid as the top line. The second column indicates accession numbers for each of the sequences and the position of the partial sequence is shown by the numbers at the top.

			481		514
***Myomorpha* (19/19)**	*Mus caroli*	XP_021041364	GFTECFGAVLPS	PSD	SQENRGLAGFQQFLKSLQ
	Meriones unguiculatus	XP_021486030	----R--V----	LPA	-- --N-D---H-M----
	Arvicanthis niloticus	XP_034380878	-------V----	---	-------G----------
	Mastomys coucha	XP_031224479	----R--V----	L--	----Q--G----------
	Mus pahari	XP_021073619	-------V----	L--	-------G----------
	Mus musculus	NP_001335221	-------V----	L--	------------------
	Grammomys surdaster	XP_028633808	-------V----	L--	-------G----------
	Rattus rattus	XP_032757347	-------V----	V--	-E-----G----------
	Rattus norvegicus	XP_017443925	-------V----	L--	-E-----G----------
	Cricetulus griseus	XP_007606236	----R--V----	--T	----CN-D---H------
	Mesocricetus auratus	XP_012973887	----R--V----	--A	-----N-D---H------
	Neotoma lepida	OBS80128	----R-------	--A	-----N-D---H------
	Peromyscus leucopus	XP_028729804	----R--V----	--A	-----H-D---H------
	Peromyscus maniculatus bairdii	XP_015846187	----R--V----	--A	-----H-D---H------
	Onychomys torridus	XP_036029593	----R--V----	--A	----HS-D---H------
	Microtus ochrogaster	XP_026642320	----R--V----	V-A	-----S-D---H------
	Arvicola amphibius	XP_038196402	----R--V----	V-A	-----S-D---H------
	Nannospalax galili	XP_029420171	----RY------	FPV	-R--HN-D---H------
	Jaculus jaculus	XP_012803803	----RY-V-V-A	FPS	PK-HSR-S---H--Q---
**Other *Euarchontoglires***	*Castor canadensis*	XP_020036107	----RY-----A		---QSR-S---H--Q---
	Dipodomys ordii	XP_012892446	----RY-----A		-KQESK-S---R--QH-
	Heterocephalus glaber	EHB05252	----RY-----P		P-QQSR---L-H--Q---
	Chinchilla lanigera	XP_013362757	----RY-----P		PR-PS----L-R--Q-V-
	Cavia porcellus	XP_023420239	----RY--I--P		-R-QSR---L----Q---
	Octodon degus	XP_023562908	----RY-----P		PR-QSR-S-L-H--Q---
	Fukomys damarensis	XP_033615327	----RY-----P		PP-QPR---L-H--Q---
	Marmota flaviventris	XP_027806874	----RY------		--LQPK-T---H------
	Ictidomys tridecemlineatus	XP_005334016	----RY------		--FQPK-T---H------
	Marmota monax	VTJ60368	----RY------		-RLQPK-T---H------
	Marmota marmota marmota	XP_015356293	----RY------		-RLQPK-T---H------
	Ochotona princeps	XP_004596500	----RY---S--		-R-Q-R-----S--Q---
	Microcebus murinus	XP_012634894	----RY-S--S-		-R-RPR--------Q---
	Otolemur garnettii	XP_023369833	----RY----S-		--KPR-T---E--QR--
	Macaca nemestrina	XP_024642873	----RY----S-		R-QPK--------Q---
	Hylobates moloch	XP_032015472	----RY----S-		R-QPK--------Q---
	Pongo abelii	PNJ30636	----RY----S-		R-QPK--------Q---
	Macaca fascicularis	XP_015285832	----RY----S-		R-QPK--------Q---
	Theropithecus gelada	XP_025258353	----RY----S-		R-QPK--------Q---
	Trachypithecus francoisi	XP_033079292	----RY----S-		R-QPK--------Q---
	Papio anubis	XP_021777710	----RY----S-		R-QPK--------Q---
	Chlorocebus sabaeus	XP_007965744	----RY----S-		R-QPK--------Q---
	Cercocebus atys	XP_011920118	----RY----S-		R-QPK--------Q---
	Rhinopithecus bieti	XP_017749759	----RY----S-		R-QPK--------Q---
	Nomascus leucogenys	XP_030660502	----RY----S-		R-QPK--------Q---
	Macaca mulatta	XP_028685079	----RY----S-		R-QPR--------Q---
	Rhinopithecus roxellana	XP_030794765	----RY----S-		R-QPK--------Q---
	Sapajus apella	XP_032110588	----RY----S-		R-QPR--------Q---
	Callithrix jacchus	XP_035111494	----RY----S-		R-QPK--------Q---
	Homo sapiens	XP_016874424	----RY---FS-		R-QPK--------Q---
	Pan paniscus	XP_034791982	----RY---FS-		R-QPK--------Q---
	Pan troglodytes	XP_016778417	----RY---FS-		R-QPK--------Q---
	Aotus nancymaae	XP_012328189	----RY----S-		R-QPK-------VQ---
	Carlito syrichta	XP_021565713	----RY---IS-		R-QPK--------Q---
	Gorilla gorilla gorilla	XP_030856791	----RY---FS-		R-QPK--------Q---

Supplementary figure S10: Multiple sequence alignment of a conserved segment of the protein ATP-dependent DNA helicase DDX11 isoform 1, showing a 3 amino acid insertion that is present in the suborder Myomorpha. This CSI was detected in all species within Myomorpha, and not detected in any other members of the Euarchontoglires in the top 250 BLASTp returns. The dashes (-) within the alignment indicate the same amino acid as the top line. The second column indicates accession numbers for each of the sequences and the position of the partial sequence is shown by the numbers at the top.

Group	Species	Accession	1150		1174
***Myomorpha* (19/19)**	*Rattus norvegicus*	EDL86529	SPQGGRRY		VQVMGSGLLAEMKAKQE
	Mus musculus	BAC31591	--------		-----------------
	Arvicanthis niloticus	XP_034366760	--------		-----------------
	Mus pahari	XP_029403371	--------		-----------------
	Meriones unguiculatus	XP_021498526	--------		-----------------
	Mastomys coucha	XP_031214755	--------		-----------------
	Mus caroli	XP_029324213	--------		-----------------
	Grammomys surdaster	XP_028632171	--------		-----------------
	Rattus rattus	XP_032740092	--------		-----------------
	Neotoma lepida	OBS77042	--------		-----------------
	Peromyscus leucopus	XP_028736411	--------		-----------------
	Onychomys torridus	XP_036044973	--------		-----------------
	Cricetulus griseus	ERE77586	---S----		-----------------
	Peromyscus maniculatus bairdii	XP_015845733	--------		-----------------
	Microtus ochrogaster	XP_026638705	---A----		--M--------------
	Mesocricetus auratus	XP_021080710	---S----		--M--------------
	Arvicola amphibius	XP_038189211	--------		--M--------------
	Jaculus jaculus	XP_012806998	--------		M-M---------R----
	Nannospalax galili	XP_008830122	---A----		-----------------
**Other *Euarchontoglires***	*Castor canadensis*	XP_020044037	--------	G	-----------------
	Fukomys damarensis	XP_033617673	--------	G	-----------------
	Heterocephalus glaber	XP_021116201	--------	G	-----------------
	Octodon degus	XP_004640197	--------	G	------S----------
	Cavia porcellus	XP_023420497	---A----	G	------S----------
	Marmota marmota marmota	XP_015349672	--------	G	-----------------
	Ictidomys tridecemlineatus	XP_040138011	--------	G	-----------------
	Marmota flaviventris	XP_027804129	--------	G	-----------------
	Urocitellus parryii	XP_026263478	--------	G	--------M--------
	Ochotona princeps	XP_004593141	--------	G	-----------------
	Oryctolagus cuniculus	XP_017200440	----S---	G	-----------------
	Tupaia chinensis	XP_014448817	--P-----	G	-----G------R---G
	Sapajus apella	XP_032124538	--------	G	-----------------
	Nomascus leucogenys	XP_030673253	--------	G	-----------------
	Macaca nemestrina	XP_024648805	--------	G	-----------------
	Papio anubis	XP_009202789	--------	G	-----------------
	Macaca mulatta	XP_014991178	--------	G	-----------------
	Microcebus murinus	XP_012596751	--------	G	-----------------
	Hylobates moloch	XP_031997093	--------	G	-----------------
	Saimiri boliviensis boliviensi	XP_010336303	--------	G	-----------------
	Callithrix jacchus	XP_035153224	--------	G	-----------------
	Rhinopithecus bieti	XP_017733988	--------	G	-----------------
	Microcebus murinus	XP_012596748	--------	G	-----------------
	Trachypithecus francoisi	XP_033072298	--------	G	-----------------
	Hylobates moloch	XP_031997090	--------	G	-----------------
	Macaca fascicularis	XP_005553936	--------	G	-----------------
	Chlorocebus sabaeus	XP_037861697	--------	G	-----------------
	Aotus nancymaae	XP_021523326	--------	G	-----------------
	Carlito syrichta	XP_021566595	--------	G	-----------------
	Rhinopithecus roxellana	XP_030785349	--------	G	-----------------
	Theropithecus gelada	XP_025237664	--------	G	-----------------
	Piliocolobus tephrosceles	XP_023065492	--------	G	-----------------
	Otolemur garnettii	XP_003788390	--------	G	-------M---------
	Pan paniscus	XP_034817451	---A----	G	-----------------
	Pan troglodytes	XP_009448885	---A----	G	-----------------
	Homo sapiens	XP_016866497	---A----	G	-----------------
	Gorilla gorilla gorilla	XP_030867899	---A----	G	-----------------
	Pongo abelii	NP_001361060	---A----	G	-----------------

Supplementary figure S11: Multiple sequence alignment of a conserved segment of the protein F-actin-uncapping protein LRRC16A, showing a 1 amino acid deletion that is present in the suborder Myomorpha. This CSI was detected in all species within Myomorpha, and not detected in any other members of the Euarchontoglires in the top 250 BLASTp returns. The dashes (-) within the alignment indicate the same amino acid as the top line. The second column indicates accession numbers for each of the sequences and the position of the partial sequence is shown by the numbers at the top.

Group	Species	Accession	737		775
**Castorimorpha (2/2)**	*Dipodomys ordii*	XP_012870168	EPQKDNCVTDSEPVVNVIP		SANPINIAIPTVTAQLPTI
	Castor canadensis	XP_020026324	-------------------		-------------------
**Myomorpha (0/11)**	*Rattus rattus*	XP_032741125	-----S-----------V-	P	-------------------
	Mus musculus	XP_030106271	-----S-----------V-	P	-------------------
	Mus caroli	XP_029327690	-----S-----------V-	P	-------------------
	Mus pahari	XP_029402939	-----S-----------V-	P	-------------------
	Mastomys coucha	XP_031220768	-----S-----------V-	P	-------------------
	Arvicola amphibius	XP_038189038	-------------------	P	-------------------
	Microtus ochrogaster	XP_013204447	-------------------	P	-------------------
	Peromyscus leucopus	XP_028728789	-----S-------------	P	-------------------
	Peromyscus maniculatus bairdii	XP_015848215	-----S-------------	P	-------------------
	Jaculus jaculus	XP_004659773	-----S-------------	P	-------------------
	Nannospalax galili	XP_008848756	-----S-----------V-	P	-------------------
**Hystricomorpha (0/3)**	*Heterocephalus glaber*	EHB07542	-----S-------------	P	-------------------
	Octodon degus	XP_004641811	-----S-------I-----	P	-------------------
	Cavia porcellus	XP_003468691	-----T-------I-----	P	-------------------
**Sciuromorpha (0/5)**	*Ictidomys tridecemlineatus*	XP_013216436	-------------------	P	-------------------
	Urocitellus parryii	XP_026258099	-----S-------------	P	-------------------
	Marmota monax	VTJ78349	-----S-------------	P	-------------------
	Marmota marmota marmota	XP_015338781	-----S-------------	P	-------------------
	Marmota flaviventris	XP_027784272	-----S-------------	P	-------------------
**Other *Euarchontoglires***	*Tupaia chinensis*	ELW48452	-----S-----------L-	P	-------------------
	Pan troglodytes	XP_016818393	-----S-------------	P	-------------------
	Homo sapiens	NP_001121600	-----S-------------	P	-------------------
	Hylobates moloch	XP_031999983	-----S-------------	P	-------------------
	Nomascus leucogenys	XP_030654779	-----S-------------	P	-------------------
	Macaca nemestrina	XP_011736587	-----S-------------	P	-------------------
	Macaca fascicularis	XP_015311217	-----S-------------	P	-------------------
	Macaca mulatta	XP_015002241	-----S-------------	P	-------------------
	Theropithecus gelada	XP_025251934	-----S-------------	P	-------------------
	Chlorocebus sabaeus	XP_008000894	-----S-------------	P	-------------------
	Pan paniscus	XP_014201891	-----S-------------	P	-------------------
	Galeopterus variegatus	XP_008566268	-----S-------------	P	-------------------
	Colobus angolensis palliatus	XP_011808331	-----S-------------	P	-------------------
	Rhinopithecus roxellana	XP_030796535	-----S-------------	P	-------------------
	Papio anubis	XP_017818138	-----S-------------	P	-------------------
	Pongo abelii	XP_024108689	-----S-------------	P	-------------------
	Sapajus apella	XP_032115580	-----S-------------	P	----------A--T-----

Supplementary figure S12: Multiple sequence alignment of a conserved segment of the protein zinc finger E-box-binding homeobox 1, showing a 1 amino acid deletion that is present in the suborder Castorimorpha. This CSI was detected in all species within Castorimorpha, and not detected in any other members of the Euarchontoglires in the top 250 BLASTp returns. The dashes (-) within the alignment indicate the same amino acid as the top line. The second column indicates accession numbers for each of the sequences and the position of the partial sequence is shown by the numbers at the top.

Group	Species	Accession	150		186
**_Castorimorpha (2/2)_**	*Dipodomys ordii*	XP_012864838	PKIEEEKSEEEGTPPNIA		MAVPTSIYQTSTGQYIAI
	Castor Canadensis	XP_020041970	------------PA--L-		------------------
**Other _Euarchontoglires_**	*Rattus norvegicus*	XP_006254125	------------------	T	------------------
	Mus musculus	XP_030106165	------------------	T	------------------
	Mus caroli	XP_007645318	------------------	T	------------------
	Mus pahari	XP_029402715	------------------	T	------------------
	Grammomys surdaster	XP_028638490	------------------	T	------------------
	Meriones unguiculatus	XP_021508799	------------------	T	------------------
	Peromyscus maniculatus bairdii	XP_015841252	------------------	T	------------------
	Cricetulus griseus	XP_007645319	------------------	T	------------------
	Mesocricetus auratus	XP_012979994	------------------	T	------------------
	Onychomys torridus	XP_036043611	------------------	T	------------------
	Arvicola amphibius	XP_038188531	------------------	T	------------------
	Microtus ochrogaster	XP_026638904	------------------	T	------------------
	Nannospalax galili	XP_017653867	------------------	T	------------------
	Jaculus jaculus	XP_004668221	---------------S--	T	------------------
	Chinchilla lanigera	XP_005398175	----------G-R-S--T	T	------------------
	Cavia porcellus	XP_023420430	----------G-R-S--T	T	------------------
	Marmota marmota marmota	XP_015338915	------------------	T	------------------
	Marmota flaviventris	XP_027784312	------------------	T	------------------
	Marmota monax	VTJ89939	------------------	T	------------------
	Urocitellus parryii	XP_026264879	------------------	T	------------------
	Ictidomys tridecemlineatus	XP_021585893	------------------	T	------------------
	Papio anubis	XP_009212522	------------------	T	------------------
	Macaca nemestrina	XP_011764208	------------------	T	------------------
	Rhinopithecus bieti	XP_017703905	------------------	T	------------------
	Rhinopithecus roxellana	XP_010387691	------------------	T	------------------
	Cercocebus atys	XP_011940110	------------------	T	------------------
	Callithrix jacchus	XP_008998412	------------------	T	------------------
	Chlorocebus sabaeus	XP_008000988	------------------	T	------------------
	Macaca mulatta	XP_028682020	------------------	T	------------------
	Otolemur garnettii	XP_023367177	------------------	T	------------------
	Trachypithecus francoisi	XP_033040135	------------------	T	------------------

Supplementary figure S 13: Multiple sequence alignment of a conserved segment of the protein cAMP-responsive element modulator, showing a 1 amino acid deletion that is present in the suborder Castorimorpha. This CSI was detected in all species within Castorimorpha, and not detected in any other members of the Euarchontoglires in the top 250 BLASTp returns. The dashes (-) within the alignment indicate the same amino acid as the top line. The second column indicates accession numbers for each of the sequences and the position of the partial sequence is shown by the numbers at the top.

Group	Species	Accession	242		277
Hystricomorpha (5/5)	*Heterocephalus glaber*	XP_004847836	GLEKIEKQLTLEKLREWT		ENLKTIEVNVQLPRF
	Fukomys damarensis	XP_010621040	---E--------------		-D-E-----------
	Octodon degus	XP_004628461	---E--------------		D--E-----------
	Chinchilla lanigera	XP_005398986	---E--------------		---E-M----H----
	Cavia porcellus	XP_003461113	---E--R--SV-------		QS-E-T---IK----
Myomorpha (0/16)	*Mus pahari*	XP_021071315	--K---E-I---------	KR	---EY-D-H-K----
	Mus musculus	EDL32356	--K-----I-----L---	KR	---EF-D-H-K----
	Mus caroli	XP_021036100	--K---E-I---------	KR	---EF-D-H-K----
	Meriones unguiculatus	XP_021509336	--K---E---S-------	KC	---E--D-H-K----
	Arvicanthis niloticus	XP_034366135	--K---E-I---------	KS	---EN-D-H-K----
	Rattus rattus	XP_032740528	--K---E-I---------	KR	---EN-D-H-K----
	Rattus norvegicus	NP_001026812	--K---E-I---------	KR	---EN-D-H-K----
	Grammomys surdaster	XP_028631526	--K---E-I-----H---	KR	---EN-D-H-K----
	Mastomys coucha	XP_031214143	--K---E-I---------	KP	---EN-D-H-K----
	Mesocricetus auratus	XP_005066467	--K---E-----------	KR	---EN-D-H-K----
	Peromyscus leucopus	XP_028737941	--K---E-------N---	KR	---E--D-H-K----
	Peromyscus maniculatus bairdii	XP_006972367	--K---E-------N---	KR	---E--D-H-K----
	Microtus ochrogaster	XP_005355070	--K---E-------N---	KR	----N-D-H-K----
	Cricetulus griseus	XP_003496835	--K---E-----------	KH	---EN-D-H-K----
	Nannospalax galili	XP_008834889	--K---E-----------	KP	-S-E--D---T----
	Jaculus jaculus	XP_004666228	--KML-E-----------	KP	---EPVD-H-R----
Castorimorpha (0/2)	*Castor canadensis*	XP_020042316	------------------	KP	---D------K----
	Dipodomys ordii	XP_012872149	--R------S-D------	KP	A--AK---H-K----
Sciuromorpha (0/4)	*Ictidomys tridecemlineatus*	XP_005335221	--K-----------Q---	KP	---DI-----K----
	Marmota flaviventris	XP_027810616	--K-----------Q---	KP	---DI-----K----
	Urocitellus parryii	XP_026254710	--K-----------Q---	KP	---EI-----K----
	Marmota marmota marmota	XP_015361603	--K-----------Q---	KP	---DI-----K----
Other Euarchontoglires	*Ochotona princeps*	XP_012785616	--KE----------N---	KP	-KMR--D-H-R----
	Oryctolagus cuniculus	XP_008272506	--QE------VD------	KP	---GVV----R----
	Tupaia chinensis	XP_006161741	--K---E-----------	KP	A--SN---H-S--K-
	Homo sapiens	BAG60342	--K---E-------H---	KP	---DF-----S----
	Gorilla gorilla gorilla	XP_018885042	--K---E-------H---	KP	---DF-----S----
	Nomascus leucogenys	XP_003272240	--K---E-------H---	KP	---DF-----S----
	Papio anubis	XP_021792624	--K---E-------H---	KP	---DF-----S----
	Macaca nemestrina	XP_011749058	--K---E-------H---	KP	---DF-----S----
	Chlorocebus sabaeus	XP_007972044	--K---E-------H---	KP	---DF-----S----
	Macaca mulatta	NP_001248512	--K---E-------H---	KP	---DF-----S----
	Rhinopithecus roxellana	XP_010360117	--K---E-----------	KP	---DF-----S----
	Cercocebus atys	XP_011938813	--KR--E-------H---	KP	---DF-----S----
	Pan troglodytes	XP_003311063	--K---E-------H---	KP	---DFV----S----
	Carlito syrichta	XP_008067564	--K---E-------H---	KP	---DIM----S----
	Cricetulus griseus	XP_003496835	--K---E-----------	KH	---EN-D-H-K----
	Saimiri boliviensis boliviensi	XP_003927448	--K-V-E-----------	KP	---SS-----S----
	Aotus nancymaae	XP_012320290	--K-V-E-------H---	KP	---SS-----S----
	Callithrix jacchus	XP_002746321	--K-V-E-----------	KP	---SS-----S----
	Pongo abelii	XP_002816400	--K---G-------H---	KP	---DF-----S----
	Sapajus apella	XP_032105986	--K-V-E-----------	KP	---SS-----N----
	Rhinopithecus bieti	XP_017705849	--K---E-------H---	KP	---DF-----S----
	Propithecus coquereli	XP_012506836	--K---E---F---H---	KP	G--G------G----
	Hylobates moloch	XP_031996583	--K---E-------H---	KP	---DF-----S----
	Macaca fascicularis	XP_005554224	--K---E-------H---	KP	---DF-----S----
	Colobus angolensis palliatus	XP_011790857	--K---E-------H---	KP	---DF-----S----
	Mandrillus leucophaeus	XP_011839928	--K---E-------H---	KP	---DF-----S----
	Theropithecus gelada	XP_025238173	--K---E-------H---	KP	---DF-----S----
	Trachypithecus francoisi	XP_033071942	--K---E-------H---	KP	---DF-----S----
	Pan paniscus	XP_003805611	--K---E-------H---	KP	---DFV----S----
	Pongo abelii	XP_002816400	--K---G-------H---	KP	---DF-----S----
	Microcebus murinus	XP_012623906	--K---EA------H---	KS	G--GVM----G----

Supplementary figure S14: Multiple sequence alignment of a conserved segment of the protein leukocyte elastase inhibitor A, showing a 2 amino acid deletion that is present in the suborder Hystricomorpha. This CSI was detected in all species within Hystricomorpha, and not detected in any other members of the Euarchontoglires in the top 250 BLASTp returns. The dashes (-) within the alignment indicate the same amino acid as the top line. The second column indicates accession numbers for each of the sequences and the position of the partial sequence is shown by the numbers at the top.

Group	Species	Accession	1040		1077
Hystricomorpha (5/5)	*Heterocephalus glaber*	XP_021112474	LQFRGTPGRGSSPPSLQ		SGSDKVLCHLTHTVPCAHQ
	Cavia porcellus	XP_003477089	------S-----LSA--		----V-A-R----------
	Chinchilla lanigera	XP_013368844	-------------S---		----T-A-R----------
	Fukomys damarensis	KFO33231	----------------L		----P-V-R----------
	Octodon degus	XP_004640750	------------LS---		----M-A-R----------
**Other *Euarchontoglires* *(2/52)***	*Mesocricetus auratus*	XP_005075038	---T---------S-SV		YS-NT-A------------
	Nannospalax galili	XP_029418136	-----AQ------STPV		YS-NT-T------------
	Rattus norvegicus	XP_017451086	-------------S-PV	Y	-S-NT-A------------
	Rattus rattus	XP_032766651	-------------S-PV	Y	-S-NT-A------------
	Mus musculus	AAH70437	-------------S-SV	Y	-S-NT-T--R---------
	Mus caroli	XP_021026992	-------------S-SV	Y	-S-NT-T--R---------
	Mus pahari	XP_021061734	-------------S-SV	Y	-S-NT-A--R---------
	Mastomys coucha	XP_031202268	-------------S-SV	Y	-S-NS-A--R---------
	Meriones unguiculatus	XP_021503585	-------A-----S-PM	Y	-S-NT-A------------
	Microtus ochrogaster	XP_005348048	-------------S-SV	Y	-S-NA-A------------
	Onychomys torridus	XP_036049085	---------S-P-S-SV	Y	-S-NT-A------------
	Cricetulus griseus	NP_001230965	-------------S-SV	Y	-S-NT-A------------
	Peromyscus maniculatus bairdii	XP_006985921	-------------S-SV	Y	-S-NT-A------------
	*Peromyscus leucopus**	XP_028742707	-------------S-SV	Y	-S-ST-A------------
	Castor canadensis	XP_020018977	------S---G--S-PV	Y	-S--A-V------------
	Dipodomys ordii	XP_012883522	-----------C-S-PV	Y	-S--A--------------
	Ictidomys tridecemlineatus	XP_021587131	----------N-SS-PV	Y	-S--T-AFR----------
	Marmota monax	VTJ54601	------------SS-PV	Y	-S--T-AFR----------
	Marmota flaviventris	XP_027788097	------------SS-PV	Y	-S--T-AFR----------
	Marmota marmota marmota	XP_015344826	------------SS-PV	Y	-S--T-AFR----------
	Urocitellus parryii	XP_026245739	------------SS-PV	Y	-S--S-AFR----------
	Oryctolagus cuniculus	XP_017199271	-------------S-PM	Y	-S--T-A------------
	Ochotona princeps	XP_004581482	-------------S-PM	Y	-S--T-T------------
	Tupaia chinensis	XP_027625098	-----------A-A-PV	Y	-T--T-A-RQ---------
	Homo sapiens	BAC11673	-------------A-PV	Y	-S--T-A------------
	Galeopterus variegatus	XP_008570367	-------------T-PM	Y	-S--T-A------------
	Pan troglodytes	PNI80934	-------------A-PV	Y	-S--T-A------------
	Pan paniscus	XP_034812213	-------------A-PV	Y	-S--T-A------------
	Gorilla gorilla gorilla	XP_030864879	-------------A-PV	Y	-S--T-A------------
	Microcebus murinus	XP_012626560	-------------A-PV	Y	-ST-T-A------------
	Carlito syrichta	XP_021574853	-------------V-PM	Y	-S--T-A-R----------
	Callithrix jacchus	XP_035131104	------S------A-AM	Y	-S--T-A------------
	Aotus nancymaae	XP_021531826	------S------A-PM	Y	-S--T-T------------
	Sapajus apella	XP_032153849	------S------A-PM	Y	-S--T-A------------
	Saimiri boliviensis boliviensis	XP_010335324	------S------A-PM	Y	-S--T-A------------
	Macaca fascicularis	EHH62517	-------------A-PV	Y	-S--T-A-R----------
	Piliocolobus tephrosceles	XP_023087434	-------------A-PV	Y	-S--T-A-R----------
	Colobus angolensis palliatus	XP_011794601	-------------A-PV	Y	-S--T-A-R----------
	Pongo abelii	XP_024100037	-------------A-PV	Y	-S--T-A-R----------
	Chlorocebus sabaeus	XP_007982245	-------------A-PV	Y	-S--T-A-R----------
	Trachypithecus francoisi	XP_033061462	-------------A-PV	Y	-S--T-A-R----------
	Macaca nemestrina	XP_011737431	-------------A-PV	Y	-S--T-A-R----------
	Cercocebus atys	XP_011888839	-------------A-PV	Y	-S--T-A-R----------
	Hylobates moloch	XP_032017636	-------------A-PV	Y	-S--T-A-R----------
	Theropithecus gelada	XP_025230561	-------------A-PV	Y	-S--T-A-R----------
	Mandrillus leucophaeus	XP_011836427	-------------A-PV	Y	-S--T-A-R----------
	Papio anubis	XP_009199176	-------------A-PV	Y	-S--T-A-R----------
	Nomascus leucogenys	XP_030667774	-------------A-PV	Y	-S--T-A-R----------
	Propithecus coquereli	XP_012519530	------A------T-PV	Y	-S--M-A------------
	Macaca mulatta	XP_028699900	-------------A-PV	Y	GS--T-A-R----------
	Rhinopithecus bieti	XP_017711196	-------------A-PV	Y	-S--T-A-R----------
	Rhinopithecus roxellana	XP_030792296	-------------A-PV	Y	-S--T-A-R----------

*Contains 2 extra S amino acids after 1060

Supplementary figure S15: Multiple sequence alignment of a conserved segment of the protein sterol regulatory element-binding protein cleavage-activating protein, showing a 1 amino acid deletion that is present in the suborder Hystricomorpha. This CSI was detected in all species within Hystricomorpha, and only detected in 2 other members of the Euarchontoglires in the top 250 BLASTp returns. The dashes (-) within the alignment indicate the same amino acid as the top line. The second column indicates accession numbers for each of the sequences and the position of the partial sequence is shown by the numbers at the top.

Group	Species	Accession	58		91
***Hystricomorpha* (5/5)**	*Fukomys damarensis*	KFO31170	FKHYQAVHDAGNDSSHG		EAELALQRDDITLLRQE
	Heterocephalus glaber	XP_012927752	--------------K--		-----------------
	Chinchilla lanigera	XP_013373706	-----------------		------K----------
	Cavia porcellus	XP_023423402	-----------S-----		------K---V------
	Octodon degus	XP_012370495	-----------S-A---		------K----------
**Other *Euarchontoglires***	*Grammomys surdaster*	XP_028638932	--------------G--	G	--S---T----------
	Rattus rattus	XP_032767582	--------------G--	G	--S---T----------
	Mus caroli	XP_021029716	--------------G--	G	--S---T----------
	Mastomys coucha	XP_031206233	--------------G--	G	--T---T----------
	Mus musculus	XP_006513587	--------------G--	G	--G---T----------
	Mus pahari	XP_021060668	--------------G--	E	--S---T----------
	Rattus norvegicus	NP_001101556	--------------G--	G	--S---T----A-----
	Meriones unguiculatus	XP_021485991	--------------GL-	G	--N---T----------
	Arvicanthis niloticus	XP_034342928	--------------G--	G	--S---T----------
	Peromyscus leucopus	XP_028737036	--------------G--	G	--N---T----------
	Cricetulus griseus	XP_035314211	----E--------AG--	G	--N---T----------
	Microtus ochrogaster	XP_005358192	--------------GL-	G	--N---T----------
	Onychomys torridus	XP_036025498	--------------G--	G	--N---T----------
	Peromyscus manculatus bairdii	XP_015844842	--------------G--	G	--N---T----------
	Jaculus jaculus	XP_004650599	-----------T-----	G	--N-D-K----------
	Nannospalax galili	XP_008822059	-----------------	G	--N---T----------
	Dipodomys ordii	XP_012876735	---------TD------	G	--N---K----------
	Castor canadensis	XP_020038308	----------D--A---	G	--S---K----------
	Marmota flaviventris	XP_027810255	-E-----------A---	G	--N---K----------
	Marmota marmota marmota	XP_015338429	-E-----------A---	G	--N---K----------
	Urocitellus parryii	XP_026252553	-E-----------A---	G	--N---K----------
	Marmota monax	VTJ71054	-E-----------A---	G	--N---K----------
	Ictidomys tridecemlineatus	XP_021581489	-E-----------AG--	G	--N---K----------
	Ochotona princeps	XP_004583318	----EV-----------	G	--H-T-K----------
	Oryctolagus cuniculus	XP_017197503	----EV-----------	G	--S---K----------
	Tupaia chinensis	ELW61492	----E------------	G	--S---K---L------
	Homo sapiens	AAH17837	----E---------G--	G	-SN---K---V------
	Piliocolobus tephrosceles	XP_023079947	----E---------G--	G	-SD---K---V------
	Cercocebus atys	XP_011888299	----E---------G--	G	-SN---K---V------
	Callithrix jacchus	XP_035113063	----E---------G--	G	-SN---K---V------
	Trachypithecus francoisi	XP_033076976	----E---------G--	G	-SN---K---V------
	Sapajus apella	XP_032153476	----E---------G--	G	-SN---K---V------
	Saimiri boliviensis boliviensis	XP_003927952	----E---------G--	G	-SN---K---V------
	Colobus angolensis palliatus	XP_011789161	----E---------G--	G	-SN---K---V------
	Chlorocebus sabaeus	XP_008002427	----E---------G--	G	-SN---K---V------
	Macaca fascicularis	XP_005571910	----E---------G--	G	-SN---K---V------
	Pan troglodytes	XP_016779463	----E---------G--	G	-SN---K---V------
	Macaca mulatta	EHH21070	----E---------G--	G	-SN---K---V------
	Hylobates moloch	XP_032026785	----E---------G--	G	-SN---K---V------
	Nomascus leucogenys	XP_030675723	----E---------G--	G	-SN---K---V------
	Pan paniscus	XP_003823443	----E---------G--	G	-SN---K---V------
	Gorilla gorilla gorilla	XP_030872526	----E---------G--	G	-SN---K---V------
	Papio anubis	XP_021778447	----E---------G--	G	-SN---K---V------
	Macaca nemestrina	XP_011743010	----E---------G--	G	-SN---K---V------
	Carlito syrichta	XP_008046444	----E----T-----Q-	G	--H-T-K----------
	Pongo abelii	XP_024112984	----E---------G--	G	-SN---K---VA-----
	Otolemur garnettii	XP_003783403	----E---------GL-	G	--N---K---L------
	Aotus nancymaae	XP_021531299	----E---------GL-	G	-SN---K---V------
	Microcebus murinus	XP_012630706	----E------------	G	--NI--K----------
	Rhinopithecus roxellana	XP_030794673	----E---------G--	G	-SN---K---V------
	Theropithecus gelada	XP_025258513	----E---------G--	G	-SN---K---V------
	Rhinopithecus bieti	XP_017723831	----E---------G--	G	-SN---K---V------
	Mandrillus leucophaeus	XP_011848574	----E---------G--	G	-SN---K---V------
	Propithecus coquereli	XP_012519448	----E------------	G	--NI--K----------
	Galeopterus variegatus	XP_008571080	----E------------	G	--D---K----------

Supplementary figure S16: Multiple sequence alignment of a conserved segment of the protein early endosome antigen 1 isoform X1, showing a 1 amino acid deletion that is present in the suborder Hystricomorpha. This CSI was detected in all species within Hystricomorpha, and not detected in any other members of the Euarchontoglires in the top 250 BLASTp returns. The dashes (-) within the alignment indicate the same amino acid as the top line. The second column indicates accession numbers for each of the sequences and the position of the partial sequence is shown by the numbers at the top.

Group	Species	Accession	42		74
**Hystricomorpha (5/5)**	*Fukomys damarensis*	XP_010632895	NHPNFRLKSPENGNK		NNVLLCEQNRQYLPSQE
	Chinchilla lanigera	XP_013371302	--------------E		--F-----T----A---
	Heterocephalus glaber	EHB09744	--------------G		--F-----T----SR--
	Octodon degus	XP_004631604	--------------E		--F-----T-P--VG--
	Cavia porcellus	XP_013013608	--------N---E-E		--F----KT----A---
**Other Euarchontoglires**	*Rattus norvegicus*	NP_001099672	---------------	K	--F------K---A---
	Mus musculus	NP_001002238	---T----------T	K	--F------K---A---
	Arvicanthis niloticus	XP_034370793	---------------	K	--F------K---A---
	Mastomys coucha	XP_031246653	---------------	K	--F------K---A---
	Grammomys surdaster	XP_028631619	---------------	K	--F------K---A---
	Meriones unguiculatus	XP_021486660	---------------	K	--F------K---AG--
	Mus pahari	XP_029398241	---------------	K	--F------K---A---
	Mus caroli	XP_021007891	---S----------A	K	--F------K---A---
	Onychomys torridus	XP_036029447	---------------	K	-SF-----SKP--T---
	Peromyscus leucopus	XP_037061793	-----------S---	K	-SF-----SKP--A---
	Cricetulus griseus	XP_035293701	---------------	K	-SF-----SK---T---
	Microtus ochrogaster	XP_005352583	-----------S---	N	-SF-----SK---AR--
	Peromyscus maniculatus bairdii	XP_015852638	-----------S---	K	-SF-----SKP--T---
	Castor canadensis	XP_020033789	---------------	K	D-F-----TK---A---
	Dipodomys ordii	XP_012873156	---------------	K	D-F-----TK---S---
	Jaculus jaculus	XP_004659282	---------------	K	--F-----VK---N-R-
	Ictidomys tridecemlineatus	XP_005320748	---------------	K	--F-----TK---V---
	Marmota marmota marmota	XP_015350390	---------------	K	--F-----TK---V---
	Marmota flaviventris	XP_027786053	---------------	K	--F-F---TK---V---
	Urocitellus parryii	XP_026244920	---------------	K	--F-----TK---V---
	Tupaia chinensis	XP_006162823	---------------	K	--F-----TK---A-R-
	Microcebus murinus	XP_012627885	---------------	K	--F-----TK---A---
	Carlito syrichta	XP_021565441	-----------I---	K	Y-F-----TK---A---
	Trachypithecus francoisi	XP_033037929	---------S-----	K	--F-----TK---A---
	Rhinopithecus roxellana	XP_030796870	---------S-----	K	--F-----TK---A---
	Cercocebus atys	XP_011898381	---------S-----	K	--F-----TK---A---
	Macaca mulatta	XP_015003713	---------S-----	K	--F-----TK---A---
	Rhinopithecus bieti	XP_017747998	---------S-----	K	--F-----TK---A---
	Theropithecus gelada	XP_025252802	---------S-----	K	--F-----TK---A---
	Chlorocebus sabaeus	XP_007962323	---------S-----	K	--F-----TK---A---
	Papio anubis	XP_017799821	---------S-----	N	--F-----TK---A---
	Colobus angolensis palliatus	XP_011783234	---------S-----	K	--F-----TK---A---
	Mandrillus leucophaeus	XP_011822889	---------S-----	K	--F-----TK---A---
	Piliocolobus tephrosceles	XP_023061996	---------S-----	K	--F-----TK---A---
	Macaca fascicularis	EHH65036	---------S-----	K	--F-----TK---A---
	Pan troglodytes	XP_016774875	---------S-----	K	--F-----TK---A---
	Gorilla gorilla gorilla	XP_018889857	---------S-----	K	--F-----TK---A---
	Homo sapiens	XP_016871903	---------S-----	K	--F-----TK---A---
	Hylobates moloch	XP_032033205	---------S-----	K	--F-----TK---A---
	Pan paniscus	XP_034787421	---------S-----	K	--F-----TK---A---
	Nomascus leucogenys	XP_030662674	---------S-----	K	--F-----TK---A---
	Pongo abelii	XP_024109640	---------S-----	K	--F-----TK---A---
	Saimiri boliviensis boliviensi	XP_010331122	---------S--A--	K	--F-----TK---A---
	Aotus nancymaae	XP_012303512	-------I-S-----	K	--F-----TK---A---
	Callithrix jacchus	XP_035125043	-R-------S-----	K	--F-----TK---A---
	Sapajus apella	XP_032137592	-R-------S-----	K	--F-----TK---A---
	Otolemur garnettii	XP_003787197	---------------	K	K-F----PTK---AT--
	Propithecus coquereli	XP_012519341	---------------	K	--F-----TK---A---
	Macaca nemestrina	XP_011714681	---------S-----	K	--F-----TK---A---
	Galeopterus variegatus	XP_008585588	---------------	K	K-F-----TK---A---

Supplementary figure S17: Multiple sequence alignment of a conserved segment of the protein tudor domain-containing protein 1, showing a 1 amino acid deletion that is present in the suborder Hystricomorpha. This CSI was detected in all species within Hystricomorpha, and not detected in any other members of the Euarchontoglires in the top 250 BLASTp returns. The dashes (-) within the alignment indicate the same amino acid as the top line. The second column indicates accession numbers for each of the sequences and the position of the partial sequence is shown by the numbers at the top.

			669		703
***Hystricomorpha* (4/5)**	*Fukomys damarensis*	XP_010632895	AVDEKEIVTDKP		SVPLTAKGKVSPLEWTWV
	Heterocephalus glaber	XP_004839072	------T-----		----S-E---N-V-----
	Chinchilla lanigera	XP_005387872	-I-G--T-----		----GTE---N-------
	Cavia porcellus	XP_013013608	-IG---T-----		----C-E--AN-------
	Octodon degus	XP_004631604	-I----T--E--	SDVFIF	T-S-GTDE--N-------
**Other *Euarchontoglires***	*Grammomys surdaster*	XP_028631619	-IE--D---E-G	SSLHAA	----AIE-TAEA------
	Meriones unguiculatus	XP_021486660	--E---V-A--A	GSLHTD	----AVERAAE----A--
	Rattus norvegicus	XP_017444348	-IE--D--A--G	SSARAS	N---AVE-A-EV------
	Rattus rattus	XP_032751452	-IE--D--A--G	SSARAA	N---ALE-A-EV------
	Mastomys coucha	XP_031246653	-IE--DTGA--D	VSVHTA	---SATE-PAEA------
	Mus pahari	XP_029398241	-IE--D-MA--D	SSIHMA	-----IE-PAEA------
	Mus musculus	AAI29955	-IK--D -A--G	SSMHTA	----AIE-PAEA------
	Mus caroli	XP_021007900	-IE--DV-A--G	SSMHTA	----AIE-PAEA------
	Cricetulus griseus	XP_035298947	--E---TAA--N	NTVHTA	----AIEET-E-------
	Peromyscus leucopus	XP_037061796	--E-P-T-A-TS	GSVHTA	N---AIEER-E-------
	Peromyscus maniculatus bairdii	XP_006978503	--E-P-T-A-TS	DSVHTA	N---AIEER-E-------
	Neotoma lepida	OBS71111	--E-T--EA--S	SSVHTA	----AIEETTE-------
	Microtus ochrogaster	XP_005352583	--V-----A--T	NGVHTV	----ATEETAES------
	Jaculus jaculus	XP_004659282	-M ---V----T	SDMKKA	I --AIE-R-E-------
	Nannospalax galili	XP_008828441	--E--D--AE-A	SDMKKA	----AIE---E--Q----
	Castor canadensis	XP_020033789	-TE---M-KE-S	SDVEKA	---SSVEE--DV-A-M--
	Dipodomys ordii	XP_012873156	-MED--M-AE-T	SDTTKA	-A--RVEE--DV------
	Ictidomys tridecemlineatus	XP_005320748	--E---MA----	SGMKKA	----GIE E-N--K----
	Marmota marmota marmota	XP_015350390	--E---MA----	SGMKKA	----GIE E-N--K----
	Urocitellus parryii	XP_026244920	--E---MA----	SGVKKA	----GIE E-N--K----
	Marmota flaviventris	XP_027786054	--E---MA----	SGMKKA	----GIE E-N--K----
	Oryctolagus cuniculus	XP_008268757	-TE-CGVL----	GDGKEA	G---GVE----A-S----
	Tupaia chinensis	XP_006162823	-- --GL--E--	SNAKEA	-G--SVE---NL------
	Pan troglodytes	XP_009457552	--G-QSM-----	SDVKET	----GVE---N-------
	Hylobates moloch	XP_032033200	--G-QSM-----	SDVKET	----GVE---N-------
	Gorilla gorilla gorilla	XP_018889857	--V-QSM-----	SDVKET	----GVE---N-------
	Pan paniscus	XP_034787421	--G-QSM-----	SDVKET	----GVE---N-------
	Mandrillus leucophaeus	XP_011822890	--G-QRM-----	SDVKED	----GVE---D-------
	Theropithecus gelada	XP_025252804	--G-QRM-----	SDVKED	----GVE---D-------
	Trachypithecus francoisi	XP_033037929	--G-QRM-----	SDMKEA	T---GVE---D-------
	Macaca fascicularis	XP_015312201	--G-QRM-----	SDVKED	----GVE---D-------
	Macaca nemestrina	XP_011714681	--G-QRM-----	SDVKED	----GVE---D-------
	Cercocebus atys	XP_011898383	--G-QRM-----	SDVKED	----GVE---D-------
	Colobus angolensis palliatus	XP_011783233	--G-QRM-----	SDMKEA	I---GVE---D-------
	Macaca mulatta	EHH19388	--G-QRM-----	SDVKED	----GVE---D-------
	Nomascus leucogenys	XP_030662676	--G-QSM-----	SDVKET	----GVE---N-------
	Piliocolobus tephrosceles	XP_031792088	--G-QRM-----	SDMKEA	I---GVE---D-------
	Papio anubis	XP_021799391	--G-QRM-----	SDVKED	----GVE---D-------
	Homo sapiens	AAK31985	--G-QSM-----	SDVKET	----GVE---N-------
	Pongo abelii	XP_024109640	--G-QS------	SDVKET	----GVE---N-------
	Chlorocebus sabaeus	XP_007962329	--G-QRM-----	SDMKED	----GVE---D-------
	Rhinopithecus roxellana	XP_030796870	--G-QRM-----	SDMKEA	I---GVE---D-------
	Rhinopithecus bieti	XP_017747998	--G-QRM-----	SDMKEA	I---GVE---D-------
	Galeopterus variegatus	XP_008585588	--G--V------	SDMKET	----GVE---N-------
	Aotus nancymaae	XP_012303511	--G-QGM-A---	SDMKEA	----GVE---N-------
	Propithecus coquereli	XP_012519341	--GG-GR-----	SDVKEA	----DVE--AN-------
	Sapajus apella	XP_032137592	--G-QGM-V---	SDMKEA	--S-GVE--IN-------
	Callithrix jacchus	XP_035125043	--G-QGM-A---	SDMKEA	----GVER--N-------
	Carlito syrichta	XP_021565441	--EK-AT-A-S-	SDVKEA	I---NVE---N----S--
	Microcebus murinus	XP_012627885	--GG-GR---S-	SDAKEA	----DVE--TN-------
	Saimiri boliviensis boliviensi	XP_010331122	--G-QGM-A---	SDMKEA	----GVE---NS------
	Otolemur garnettii	XP_003787197	-LG--GRL----	SDVKEA	----GVE---N----MC-

Supplementary figure S18: Multiple sequence alignment of a conserved segment of the protein tudor domain-containing protein 1, showing a 6 amino acid deletion that is present in the suborder Hystricomorpha. This CSI was detected in all species within Hystricomorpha, except 1, and not detected in any other members of the Euarchontoglires in the top 250 BLASTp returns. The dashes (-) within the alignment indicate the same amino acid as the top line. The second column indicates accession numbers for each of the sequences and the position of the partial sequence is shown by the numbers at the top.

Group	Species	Accession	659		687
***Hystricomorpha* (3/5)**	*Cavia porcellus*	XP_003464075	LQPGQAPVGRAPSTMTGSG	DR	VDARTASSGS
	Chinchilla lanigera	XP_013375293	-------M-----------	--	----------
	Octodon degus	XP_023578858	-------M--V--------	N-	----------
	Fukomys damarensis	XP_010610154	-H-----T-----------		----------
	Heterocephalus glaber	XP_004864595	-------T--V--------		----------
***Muridae* (1/9)**	*Meriones unguiculatus*	XP_021520472	------LQ-----------	--	----------
	Mastomys coucha	XP_031223901	-------Q-----------		----------
	Grammomys surdaster	XP_028616456	-------Q--V--------		----------
	Arvicanthis niloticus	XP_034353756	-------Q--V--------		----------
	Rattus norvegicus	NP_001014240	-------Q--V--------		----------
	Mus pahari	XP_021053057	----T--Q-----------		----------
	Mus musculus	XP_011236992	----A--Q--V--------		----------
	Mus caroli	XP_021013058	----T--Q--V--------		----------
	Rattus rattus	XP_032757273	-------Q--V-N------		----------
***Cricetidae* (1/6)**	*Neotoma lepida*	OBS60593	-------Q-----------	--	----------
	Cricetulus griseus	XP_007646386	-------Q-----------		----------
	Peromyscus maniculatus bairdii	XP_006972223	-----L-Q-----------		----------
	Peromyscus leucopus	XP_028748940	-----P-Q-----------		----------
	Microtus ochrogaster	XP_005361654	-----G-Q--V--------		----------
	Onychomys Torridus	XP_036029397	-------Q--V--------		----------
**Other *Euarchontoglires***	*Nannospalax galili*	XP_008823974	-------P-----------		----------
	Jaculus jaculus	XP_004662969	----P--A-----------		----------
	Castor canadensis	XP_020026405	-------T-----------		----------
	Dipodomys ordii	XP_012885443	-----V-A--V--------		----------
	Ictidomys tridecemlineatus	XP_005330557	-------T-----------		----------
	Marmota marmota marmota	XP_015336897	-------T-----------		----------
	Urocitellus parryii	XP_026246098	-------T-----------		----------
	Marmota flaviventris	XP_027797861	-------T-----------		----------
	Ochotona princeps	XP_004576870	-----G-T---A-------		----------
	Oryctolagus cuniculus	XP_008257421	-----G-T-----------		----------
	Tupaia chinensis	XP_006168698	-------T-----------		----------
	Otolemur garnettii	XP_023366087	-------T-----------		----------
	Propithecus coquereli	XP_012497971	-------T-----------		----------
	Carlito syrichta	XP_008057064	-------T-----------		----------
	Microcebus murinus	XP_012595636	-------P-----------		----------
	Pan troglodytes	PNI70771	-------T---H-------		----------
	Pongo abelii	PNJ36637	-------T---H-------		----------
	Homo sapiens	CAD98061	-------T---H-------		----------
	Colobus angolensis palliatus	XP_011787610	-------T---H-------		----------
	Gorilla gorilla gorilla	XP_030864755	-------T---H-------		----------
	Piliocolobus tephrosceles	XP_023077228	-------T---H-------		----------
	Rattus rattus	XP_032757273	-------Q--V-N------		----------
	Sapajus apella	XP_032134000	--S----T-----------		----------
	Saimiri boliviensis boliviensi	XP_003925553	--S----T-----------		----------
	Pan paniscus	XP_034811078	-------T---H-------		----------
	Rhinopithecus roxellana	XP_010358586	-------T--VH-------		----------
	Nomascus leucogenys	XP_030658261	-----V-T---H-------		----------
	Aotus nancymaae	XP_012301331	--S--V-T-----------		----------
	Callithrix jacchus	XP_002749853	--S--V-T-----------		----------
	Papio anubis	XP_003908027	---V---T---H-------		----------
	Macaca mulatta	NP_001244824	---V---T---H-------		----------
	Macaca fascicularis	XP_005574421	---V---T---H-------		----------
	Galeopterus variegatus	XP_008585685	-------T-----------		----------
	Trachypithecus francoisi	XP_033086033	-------T---H-------		----------
	Hylobates moloch	XP_032610329	-----V-T---H-------		----------
	Rhinopithecus bieti	XP_017712228	-------T--VH-------		----------
	Aotus nancymaae	XP_012301331	--S--V-T-----------		----------
	Chlorocebus sabaeus	XP_007964549	---V---T---H-------		----------
	Mandrillus leucophaeus	XP_011820038	---V---T---H-------		----------
	Macaca nemestrina	XP_011739014	---V---T---H-------		----------

Supplementary figure S19: Multiple sequence alignment of a conserved segment of the protein autophagy-related protein 9A isoform a, showing a 2 amino acid insertion that is present in the suborder Hystricomorpha. This CSI was detected in all species within Hystricomorpha except for 2, and only detected in 2 other members of the Euarchontoglires in the top 250 BLASTp returns. The dashes (-) within the alignment indicate the same amino acid as the top line. The second column indicates accession numbers for each of the sequences and the position of the partial sequence is shown by the numbers at the top.

Group	Species	Accession	429		469
**Hystricomorpha (5/5)**	*Fukomys damarensis*	XP_010621282	QIFAHIAGAISPTIAGVFISQDS	NA	EFGWRNVFLVAAAIDIVG
	Heterocephalus glaber	XP_021116189	-V----S------V---------	--	------------------
	Cavia porcellus	XP_003468825	-V-----------VT------G-	DS	-S----------------
	Chinchilla lanigera	XP_005403399	-V-S---------VT--L---G-	DS	-A-------I--------
	Octodon degus	XP_023555781	-V----------SVT--LL--A-	SS	-L-------I---V-L--
**Other Euarchontoglires**	*Rattus rattus*	XP_032740413	-V-SYL--G-A--V--F-----A		---------L-----V--
	Mus musculus	XP_006516763	-V-SYL--G-A--V--F------		--------FL-----V--
	Arvicanthis niloticus	XP_034366605	-V-SYL--G-A--V--F------		---------L-----V--
	Mastomys coucha	XP_031214967	-V-SYL--G-A--V--F------		---------L---V-V--
	Rattus norvegicus	NP_001258143	-V-SYL--G-A--V--F------		---------L-----V--
	Grammomys surdaster	XP_028632155	-V-SYL--G-A--V--F------		--------IL-----V--
	Mus pahari	XP_021071518	-V-SYL--G-A--V--F------		--------FL-----V--
	Meriones unguiculatus	XP_021500563	-V-SYL--G-A--V--F-----T		-S-------L----NVA-
	Onychomys torridus	XP_036045116	-V-S----G-A--V--F-L---P		---------L-----V--
	Peromyscus maniculatus bairdii	XP_015842147	-V-S----G-A--V--F-L---P		---------L-----V--
	Peromyscus leucopus	XP_028736343	-V-S----G-A--V--F-L---P		---------L-----V--
	Mesocricetus auratus	XP_021080723	-V-S--S-G-A--V--F--G--P		---------L-----V--
	Cricetulus griseus	XP_035297405	-V-S--S-G-A--V--F--G--P		--------FL-----V--
	Microtus ochrogaster	XP_005355017	---SY----VA--A--F--G--P		-L-------L-----V--
	Jaculus jaculus	XP_004670048	-V----S---A--A--F------		--------FL--V---F-
	Nannospalax galili	XP_029418848	-V-S---AGVA--V--F------		-M-------L--G---G-
	Castor canadensis	XP_020043741	-V-----------AT-F------		-------------VN-S-
	Dipodomys ordii	XP_012890697	-T--Y-S------AT-F------		-W------I--T-VN-S-
	Marmota monax	VTJ51881	-------------V---L-N--P		-V-------L---VN-S-
	Ictidomys tridecemlineatus	XP_005337090	-------------V---L-N--P		-V----M--L---VN-S-
	Marmota flaviventris	XP_027803862	-------------V---L-N--P		-V----M--L---VN-S-
	Marmota marmota marmota	XP_015349573	-------------V---L-N--P		-V----M--L---VN-S-
	Urocitellus parryii	XP_026263508	-------------V---L-N--P		-V----M--L---VN-S-
	Oryctolagus cuniculus	XP_017200301	-V--IL-------V---LVG---		-L-------L------S-
	Ochotona princeps	XP_012786159	-L-VA-D-V-A--V--IL-N---		VL----I--I-S--N---
	Pongo abelii	XP_024104170	-V-----------A--F------		---------LS--VN-S-
	Pan paniscus	XP_034818193	-V-----------A--F------		---------LS--VN-S-
	Homo sapiens	XP_011512521	-V-----------A--F------		---------LS--VN-S-
	Papio anubis	XP_031522836	-V-----------A--F------		---------LS--VN-S-
	Macaca mulatta	XP_014991186	-V-----------A--F------		---------LS--VN-S-
	Macaca nemestrina	XP_024648826	-V-----------A--F------		---------LS--VN-S-
	Macaca fascicularis	XP_015305296	-V-----------A--F------		---------LS--VN-S-
	Galeopterus variegatus	XP_008570086	-V-S------------F------		---------LS--VN-L-
	Otolemur garnettii	XP_003788823	-V-----------V--F------		---------LS---N-P-
	Rhinopithecus roxellana	XP_030785196	-V-----------A--F------		---------LS--VN-S-
	Pan troglodytes	PNI24959	-V-----------A--F------		---------LS--VN-S-
	Rhinopithecus bieti	XP_017733980	-V-----------A--F------		---------LS--VN-S-
	Gorilla gorilla gorilla	XP_004043410	-V-----------A--F------		---------LS--VN-S-
	Aotus nancymaae	XP_012313961	-V-----------V--F--N---		---------LS--VN-S-
	Sapajus apella	XP_032124514	-V-----------V--F--N---		---------LS--VN-S-
	Callithrix jacchus	XP_002746207	-V-----------V--F--N---		---------LS--VN-S-
	Carlito syrichta	XP_008052342	-V-----------V--F-L----		D--------LS-GVN-L-
	Microcebus murinus	XP_012596812	-V---T------STV-F------		---------LS---N-L-
	Cercocebus atys	XP_011886906	-V-----------A--F------		D-F------LS---N-S-
	Propithecus coquereli	XP_012494145	-V--YT------ATV-F------		------A--LS---N-L-
	Colobus angolensis palliatus	XP_011818208	-V-----------A--F------		---------LS--VN-S-
	Piliocolobus tephrosceles	XP_023065423	-V-----------A--F------		---------LS--VN-S-
	Theropithecus gelada	XP_025239218	-V-----------A--F------		---------LS--VN-S-
	Hylobates moloch	XP_031996747	-V-----------A--F------		---------LS--VN-S-
	Nomascus leucogenys	XP_003263282	-V-----------A--F------		---------LS--VN-S-
	Mandrillus leucophaeus	XP_011821870	-V-----------A--F------		---------LS---N-S-
	Chlorocebus sabaeus	XP_037861704	-V-----------A--F------		---------LS--VN-S-
	Trachypithecus francoisi	XP_033072321	-V-------T---A--F------		---------LS--VN-S-
	Saimiri boliviensis boliviensi	XP_003927336	-V-----------V--F--N---		---------LS--VN-S-

Supplementary figure S20: Multiple sequence alignment of a conserved segment of the protein probable small intestine urate exporter, showing a 2 amino acid insertion that is present in the suborder Hystricomorpha. This CSI was detected in all species within Hystricomorpha, and not detected in any other members of the Euarchontoglires in the top 250 BLASTp returns. The dashes (-) within the alignment indicate the same amino acid as the top line. The second column indicates accession numbers for each of the sequences and the position of the partial sequence is shown by the numbers at the top.

Group	Species	Accession	1326		1358
***Sciuromorpha* (5/5)**	*Urocitellus parryii*	XP_026267995	GGLPGAGLFGPKNELEDYD		DSDFEVLMKTAHGHLVSDR
	Ictidomys tridecemlineatus	XP_021578779	-------------------		-------------------
	Marmota marmota marmota	XP_015359843	-------------D-----		-------------------
	Marmota flaviventris	XP_027803793	-------------D-----		-------------------
	Marmota monax	VTJ63594	-------------D-----		-------------------
***Myomorpha* (0/15)**	*Mus pahari*	XP_029403289	-------FY----D---F-	V	----------------P--
	Mus caroli	XP_029324152	-------FY----D---F-	V	----------------P--
	Mus musculus	NP_076357	-------FY----D---F-	V	----------------P--
	Rattus rattus	XP_032741093	--I----FY----D---F-	V	----------------P--
	Rattus norvegicus	ABY79796	--I----FY----D---F-	V	----------------P--
	Mastomys coucha	XP_031213827	------SFY----D---F-	V	----------------P--
	Meriones unguiculatus	XP_021517114	--------Y---SD-----	V	----------------P--
	Grammomys surdaster	XP_028625688	-----T-FY----D---F-	V	----------------P--
	Peromyscus leucopus	XP_028715489	--------Y----D-----	V	----------------P--
	Peromyscus maniculatus bairdii	XP_015854219	--------Y----D-----	V	----------------P--
	Cricetulus griseus	XP_035297609	--------Y----D-----	V	----------------P--
	Onychomys torridus	XP_036043836	--------Y----D-----	V	----------------P--
	Mesocricetus auratus	XP_021083809	--------Y----D-----	V	----------------P--
	Arvicola amphibius	XP_038190214	--------Y----D-----	V	----------------P--
	Microtus ochrogaster	XP_026644150	--------Y----D-----	V	----------------P--
***Castorimorpha* (0/2)**	*Castor canadensis*	XP_020013049	------------SD-----	A	----------------P--
	Dipodomys ordii	XP_012864681	---S-----V---D-----	T	----------------P--
***Hystricomorpha* (0/5)**	*Fukomys damarensis*	XP_033617665	-------------D-----	A	----------------P--
	Heterocephalus glaber	EHA98991	-------------D-----	A	----------------P--
	Cavia porcellus	XP_023422283	-------------D-----	A	----------------P--
	Octodon degus	XP_023556594	-------------D-----	A	----------------P--
	Chinchilla lanigera	XP_013360154	---A---------D-----	A	----------------P--
**Other *Euarchontoglires***	*Oryctolagus cuniculus*	NP_001076226	-------------D-----	A	----------------P--
	Ochotona princeps	XP_036351073	-------------D-----	A	----------------P--
	Tupaia chinensis	XP_027626632	-----T-------D-----	A	---------------A---
	Macaca fascicularis	EHH50900	-------------D-----	A	----------------P--
	Galeopterus variegatus	XP_008567456	-------------D-----	A	----------------P--
	Sapajus apella	XP_032098310	-------------D-----	A	----------------P--
	Saimiri boliviensis boliviensi	XP_010344223	-------------D-----	A	----------------P--
	Aotus nancymaae	XP_021532857	-------------D-----	A	----------------P--
	Pan paniscus	XP_014198912	-------------D-----	A	----------------P--
	Pan troglodytes	XP_016797023	-------------D-----	A	----------------P--
	Gorilla gorilla gorilla	XP_030857183	-------------D-----	A	----------------P--
	Pongo abelii	XP_024091969	-------------D-----	A	----------------P--
	Rhinopithecus roxellana	XP_030791948	-------------D-----	A	----------------P--
	Homo sapiens	XP_006711868	-------------D-----	A	----------------P--
	Papio anubis	XP_021789843	-------------D-----	A	----------------P--
	Macaca mulatta	XP_014982138	-------------D-----	A	----------------P--
	Chlorocebus sabaeus	XP_007988097	-------------D-----	A	----------------P--
	Theropithecus gelada	XP_025257659	-------------D-----	A	----------------P--
	Trachypithecus francoisi	XP_033056051	-------------D-----	A	----------------P--
	Macaca nemestrina	XP_011727740	-------------D-----	A	----------------P--
	Piliocolobus tephrosceles	XP_031790761	-------------D-----	A	----------------P--
	Rhinopithecus bieti	XP_017717734	-------------D-----	A	----------------P--
	Microcebus murinus	XP_020137249	-------------D-----	A	----------------P--
	Propithecus coquereli	XP_012515607	-------------D-----	A	----------------P--
	Carlito syrichta	XP_008057423	------S------D-----	A	----------------P--
	Colobus angolensis palliatus	XP_011791152	-------------D-----	A	---------------AP--
	Hylobates moloch	XP_031993535	---S---------D-----	A	----------------P--
	Nomascus leucogenys	XP_030668484	---S---------D-----	A	----------------P--
	Callithrix jacchus	XP_035136403	------------SD-----	A	----------------P--
	Cercocebus atys	XP_011891768	-------------D-----	A	----------------P--
	Otolemur garnettii	XP_023375014	-------------D-----	A	----------------PGV

Supplementary figure S21: Multiple sequence alignment of a conserved segment of the protein ryanodine receptor 2, showing a 1 amino acid deletion that is present in the suborder Sciuromorpha. This CSI was detected in all species within Sciuromorpha, and not detected in any other members of the Euarchontoglires in the top 250 BLASTp returns. The dashes (-) within the alignment indicate the same amino acid as the top line. The second column indicates accession numbers for each of the sequences and the position of the partial sequence is shown by the numbers at the top.

Group	Species	Accession	1072		1109
***Sciuromorpha* (5/5)**	*Ictidomys tridecemlineatus*	XP_005337060	QRRHATCHG		AQAPVPASFCQHSPKPATVRSCQAGPC
	Urocitellus parryii	XP_026251363	---------		---------------------------
	Marmota monax	VTJ54367	---------		---------------------------
	Marmota flaviventris	XP_027798063	---------		---------------------------
	Marmota marmota marmota	XP_015361201	---------		--------------R--A---------
**Other *Euarchontoglires* (2/54)**	*Dipodomys ordii*	XP_012884837	-Y-RD--LE		--S---------L---V---G-WVE--
	Castor canadensis	XP_020034606	-H-RD--L-	HQ	-H-----N--W-L-------G-W-R--
	Tupaia chinensis	XP_027622423	-L--D--L-	L	--E----V----L-------G-W----
	Rattus rattus	XP_032758886	----G--L-	PQ	--V----N----L---V---G-W----
	Rattus norvegicus	XP_006233941	----G--L-	PQ	--V----N----L---V---G-W----
	Mus pahari	XP_029392628	----D--L-	PQ	--V----N----L---V---G-W----
	Mus musculus	NP_001001322	----D--L-	PQ	--V----N----L---M---G-W----
	Mastomys coucha	XP_031229198	----D--I-	PQ	--V----N----L---V---G-WT---
	Mus caroli	XP_021041805	----D--L-	PQ	--V---DN----L---V---G-W----
	Grammomys surdaster	XP_028612965	----D--L-	PQ	--V----N----L---V---A-W----
	Meriones unguiculatus	XP_021507617	-H--D--L-	PQ	--V----N----L---V-----W----
	Cricetulus griseus	EGW06892	----D--L-	PQ	--V---------L---V---G-W----
	Mesocricetus auratus	XP_012980154	----D--L-	PQ	--V---------L---V---G-W----
	Onychomys torridus	XP_036039162	----D--L-	PQ	--V---------L---V---G-W----
	Neotoma lepida	OBS81646	----D--L-	PQ	--V---------L---V---G-W----
	Microtus ochrogaster	XP_005346281	----E--L-	PQ	--V---------L---V---G-W----
	Peromyscus leucopus	XP_028714575	----D--L-	PQ	--V---------L---V---G-W----
	Peromyscus maniculatus bairdii	XP_015842116	----D--L-	PQ	--V---------L---V---G-W----
	Nannospalax galili	XP_029424617	----D--L-	PQ	-HV-M-DN--R-L---V---G-W----
	Jaculus jaculus	XP_012803270	----N--L-	PQ	-W-----Y----L---V---N-W----
	Fukomys damarensis	KFO21511	---RDS-L-	PQ	G---L-------L---V---G-W----
	Heterocephalus glaber	XP_012929301	---RD--L-	PQ	G---L------YL---VM-QG-W----
	Chinchilla lanigera	XP_013362015	----G--L-	PQ	G---L-P-----L------QG-W----
	Cavia porcellus	XP_023422184	----D--L-	PQ	G---L-------L------QG-W----
	Octodon degus	XP_023556169	----D--L-	PQ	G---L-----Y-L------QG-W----
	Oryctolagus cuniculus	XP_017195933	----DA-L-	PQ	----M--D----L-------G-W----
	Colobus angolensis palliatus	XP_011786531	----D--L-	PQ	-------D----L---V---G-W----
	Microcebus murinus	XP_012637448	-S-QD--L-	PP	-G-----N----L---V-L-G-W----
	Chlorocebus sabaeus	XP_008003967	----D--L-	PQ	-------D----L---V---G-W----
	Homo sapiens	XP_016869721	---RD--L-	PQ	-------D----L---V---G-W----
	Hylobates moloch	XP_032021449	---RD--L-	PQ	-------D----L---V---G-W----
	Gorilla gorilla gorilla	XP_030870731	---RD--L-	PQ	-------D----L---V---G-W----
	Pongo abelii	XP_024108651	---RN--L-	LQ	-------D----L---V---G-W----
	Carlito syrichta	XP_021574610	---RDV-L-	LP	-------G----L---V---G-W----
	Pan paniscus	XP_034785301	---RD--L-	PQ	V------D----L---V---G-W----
	Pan troglodytes	PNI68700	---RD--L-	PQ	V------D----L---V---G-W----
	Cercocebus atys	XP_011904041	----D--L-	PQ	-------D----L---V---G-W----
	Callithrix jacchus	XP_035117987	---RD--L-	PQ	--E---TDL---L---V---G-W----
	Aotus nancymaae	XP_021527064	---RD--L-	PQ	------TDL---L---V---G-W----
	Macaca fascicularis	XP_005580570	---RD--L-	PQ	-----L-D----L---V---G-W----
	Sapajus apella	XP_032117882	---RDI-L-	PQ	------TA----L---V---G-W----
	Otolemur garnettii	XP_023371873	-H-QS--L-	PQ	-G-----N--E-R---V---G-W----
	Saimiri boliviensis boliviensis	XP_010350548	---RD--LV	PQ	------TA----L---V---G-W----
	Macaca nemestrina	XP_011722956	----D--L-	PQ	-------D----L---V---G-W----
	Macaca mulatta	EHH23703	----D--L-	PQ	-------D----L---V---G-W----
	Nomascus leucogenys	XP_030673974	----D--L-	PQ	-------D----L---V---G-W----
	Trachypithecus francoisi	XP_033094558	----D--L-	PQ	--V----D----L---V---G-W----
	Papio anubis	XP_031510637	----D--L-	PQ	-----L-D----L---V---G-W----
	Rhinopithecus bieti	XP_017704553	----D--L-	PQ	-------D----L---V---G-W----
	Theropithecus gelada	XP_025214375	----D--L-	PQ	-------D----L---V---G-W----
	Rhinopithecus roxellana	XP_030775336	----D--L-	PQ	-------D----L---V---G-W----
	Piliocolobus tephrosceles	XP_023044758	----D--L-	PQ	-------D----L---V---G-W----
	Galeopterus variegatus	XP_008591558	-H--D--L-	PQ	-KV--S-N----L-------G-W----
	Propithecus coquereli	XP_012520167	---QD--L-	PR	-G-----N----L---V-L-G-W----

Supplementary figure S22: Multiple sequence alignment of a conserved segment of the protein A disintegrin and metalloproteinase with thrombospondin motifs 13 isoform 1 preproprotein, showing a 2 amino acid deletion that is present in the suborder Sciuromorpha. This CSI was detected in all species within Sciuromorpha, and only detected in 2 other members of the Euarchontoglires in the top 250 BLASTp returns. The dashes (-) within the alignment indicate the same amino acid as the top line. The second column indicates accession numbers for each of the sequences and the position of the partial sequence is shown by the numbers at the top.

Group	Species	Accession	472		503
**Sciuromorpha (5/5)**	*Marmota marmota marmota*	XP_015356472	PQLHFLDTDDEVSPTSW		DSRQAQASYYKFQN
	Marmota flaviventris	XP_027780286	-----------------		--------------
	Urocitellus parryii	XP_026244551	-----------------		--------------
	Ictidomys tridecemlineatus	XP_005327967	-----------------		--------------
	Marmota monax	VTJ55200	-----------------		--------------
**Other *Euarchontoglires***	*Grammomys surdaster*	XP_028615001	-----------------	G	--------------
	Mus musculus	EDL12790	-----------I-----	G	--------------
	Mastomys coucha	XP_031210515	-----------I-----	G	--------------
	Meriones unguiculatus	XP_021497748	-----------I-----	G	--------------
	Mus caroli	XP_021031891	-----------I-----	G	--------------
	Mus pahari	XP_029401766	-----------I-----	G	--------------
	Rattus norvegicus	NP_001099278	-----------I-----	G	--------F-----
	Rattus rattus	XP_032770340	-----------I-----	G	--------F-----
	Cricetulus griseus	EGW14221	-----------I-----	G	--------------
	Peromyscus leucopus	XP_037057384	-----------I-----	G	--------------
	Peromyscus maniculatus bairdii	XP_015851651	-----------I-----	G	--------------
	Microtus ochrogaster	XP_005349619	-----------I-----	G	--------------
	Mesocricetus auratus	XP_021081269	-----------I-----	G	--------------
	Onychomys torridus	XP_036052736	-----------I-----	G	--------------
	Jaculus jaculus	XP_004667902	-----------------	S	--------------
	Nannospalax galili	XP_008827024	-----------------	G	----S---------
	Castor canadensis	XP_020010105	-----------------	G	--------------
	Fukomys damarensis	KFO25509	-----------------	G	--------------
	Chinchilla lanigera	XP_005402644	-----------------	G	--------------
	Cavia porcellus	XP_005003603	-----------------	S	--------------
	Heterocephalus glaber	EHB07104	-----------------	G	------T-------
	Ochotona princeps	XP_004594022	-----------I-----	T	--------------
	Oryctolagus cuniculus	XP_002718926	-----------------	S	--------------
	Tupaia chinensis	XP_006167628	-----------------	S	--------------
	Carlito syrichta	XP_008056401	-----------------	G	--------------
	Galeopterus variegatus	XP_008563871	-----------------	N	--------------
	Homo sapiens	EAW90558	-----------------	G	--------------
	Aotus nancymaae	XP_012303757	-----------------	G	--------------
	Nomascus leucogenys	XP_030656016	-----------------	G	--------------
	Hylobates moloch	XP_031994011	-----------------	G	--------------
	Pongo abelii	NP_001125708	-----------------	G	--------------
	Saimiri boliviensis boliviensis	XP_003933135	-----------------	G	--------------
	Pan paniscus	XP_008968213	-----------------	G	--------------
	Gorilla gorilla gorilla	XP_004058290	-----------------	G	--------------
	Sapajus apella	XP_032119304	-----------------	G	--------------
	Microcebus murinus	XP_020136451	-----------------	G	--------------
	Macaca nemestrina	XP_024645647	-----------------	G	--------------
	Rhinopithecus bieti	XP_017722420	-----------------	G	--------------
	Pan troglodytes	XP_016786654	-----------------	G	--------------
	Trachypithecus francoisi	XP_033041008	-----------------	G	--------------
	Chlorocebus sabaeus	XP_008008009	-----------------	G	--------------
	Macaca fascicularis	XP_005582525	-----------------	G	--------------
	Macaca mulatta	XP_014973691	-----------------	S	--------------
	Papio anubis	XP_017805727	-----------------	G	--------------
	Cercocebus atys	XP_011911824	-----------------	G	--------------
	Otolemur garnettii	XP_023374466	-----------------	G	--------------
	Rhinopithecus roxellana	XP_010386794	-----------------	G	--------------
	Propithecus coquereli	XP_012508495	-----------------	G	--------------
	Callithrix jacchus	XP_002747855	-----------------	G	--------------
	Piliocolobus tephrosceles	XP_026305383	-----------------	G	--------------
	Galeopterus variegatus	XP_008563871	-----------------	N	--------------
	Colobus angolensis palliatus	XP_011811457	-----------------	G	---P----------
	Theropithecus gelada	XP_025218636	-----------------	S	--------------

Supplementary figure S23: Multiple sequence alignment of a conserved segment of the protein telomerase-binding protein EST1A, showing a 1 amino acid deletion that is present in the suborder Sciuromorpha. This CSI was detected in all species within Sciuromorpha, and not detected in any other members of the Euarchontoglires in the top 250 BLASTp returns. The dashes (-) within the alignment indicate the same amino acid as the top line. The second column indicates accession numbers for each of the sequences and the position of the partial sequence is shown by the numbers at the top.

Group	Species	Accession	816		844
Sciuromorpha (5/5)	*Urocitellus parryii*	XP_026252783	SSTPAADYFLQQKEYF	I	IIFLLILLQVIIN
	Marmota flaviventris	XP_027776668	----------------	-	-------------
	Ictidomys tridecemlineatus	XP_005330494	----------------	-	-------------
	Marmota marmota marmota	XP_015338459	----------------	-	-------------
	Marmota monax	KAF7475359	----------------	-	-------------
**Other *Euarchontoglires***	*Rattus norvegicus*	AAI68899	-----T-------D--		V------------
	Meriones unguiculatus	XP_021518030	-----T-------D--		V------------
	Mastomys coucha	XP_031206236	-----T-------D--		-------------
	Mus pahari	XP_021061119	-----T-------D--		V------------
	Rattus rattus	XP_032768198	-----T-------D--		V------------
	Mus musculus	XP_006513700	-----T-------D--		V------------
	Grammomys surdaster	XP_028614865	-----T-------D--		V---------L--
	Mus caroli	XP_021029418	-----T-------D--		V---------V--
	Peromyscus leucopus	XP_028728884	-----T-------D--		V------------
	Peromyscus maniculatus bairdii	XP_015848754	-----T-------D--		V------------
	Mesocricetus auratus	XP_021081654	--A--T-------D--		V------------
	Cricetulus griseus	XP_007645280	--A--T-------D--		V------------
	Microtus ochrogaster	XP_013205806	--A--T-F-----D--		-------------
	Onychomys torridus	XP_036025466	-----T-------D--		V------------
	Jaculus jaculus	XP_004650183	-----T-------D--		-------------
	Nannospalax galili	XP_029411371	--R--T-------D--		V------------
	Castor canadensis	XP_020026533	-----T-------D--		-------------
	Dipodomys ordii	XP_012874201	P----T-------D--		-------------
	Fukomys damarensis	KFO26683	-------------D--		----------T--
	Chinchilla lanigera	XP_005374401	-----T-------D--		-------------
	Octodon degus	XP_023571844	-----T-------D--		----------T--
	Cavia porcellus	XP_003475959	-------------D--		----------T--
	Oryctolagus cuniculus	XP_008255048	-----T-------D--		-------------
	Ochotona princeps	XP_036348377	-----T-------D--		-------F-----
	Tupaia chinensis	ELW70516	-----T-------D--		-------------
	Homo sapiens	BAD18783	-----T-------D--		-------------
	Pongo abelii	XP_009246323	-------------D--		-------------
	Rhinopithecus roxellana	XP_030794638	-----T-------D--		-------------
	Chlorocebus sabaeus	XP_008002267	-----T-------D--		-------------
	Pan paniscus	XP_034792149	-----T-------D--		-------------
	Microcebus murinus	XP_012605820	-----T-------D--		-------------
	Rhinopithecus bieti	XP_017728005	-----T-------D--		-------------
	Macaca fascicularis	XP_005571650	-----T-------D--		-------------
	Macaca nemestrina	XP_011725394	-----T-------D--		-------------
	Trachypithecus francoisi	XP_033077099	-----T-------D--		-------------
	Saimiri boliviensis boliviensi	XP_003927867	-----T-------D--		-------------
	Gorilla gorilla gorilla	XP_018894631	-----T-------D--		-------------
	Macaca mulatta	NP_001247808	-----T-------D--		-------------
	Sapajus apella	XP_032152084	-----T-------D--		-------------
	Nomascus leucogenys	XP_030675796	-----T-------D--		-------------
	Hylobates moloch	XP_032026687	-----T-------D--		-------------
	Papio anubis	XP_017801508	-----T-------D--		-------------
	Aotus nancymaae	XP_012296796	-----T-------D--		-------------
	Callithrix jacchus	XP_009002509	-----T-------D--		-------------
	Pan troglodytes	XP_009424152	-----T-------D--		-------------
	Cercocebus atys	XP_011905310	-----T-------D--		-------------
	Otolemur garnettii	XP_023365011	-----T-------D--		----------V--
	Carlito syrichta	XP_021568913	-----T-------D--		-------------
	Theropithecus gelada	XP_025257180	-----T-------D--		-------------
	Colobus angolensis palliatus	XP_011784762	-----T-------D--		-------------
	Propithecus coquereli	XP_012503812	-----T-------D--		L------------

Supplementary figure S24: Multiple sequence alignment of a conserved segment of the protein oxysterol-binding protein-related protein 8 isoform b, showing a 1 amino acid insertion that is present in the suborder Sciuromorpha. This CSI was detected in all species within Sciuromorpha, and not detected in any other members of the Euarchontoglires in the top 250 BLASTp returns. The dashes (-) within the alignment indicate the same amino acid as the top line. The second column indicates accession numbers for each of the sequences and the position of the partial sequence is shown by the numbers at the top.

Group	Species	Accession	36 ... 67
***Sciuromorpha* (5/5)**	*Ictidomys tridecemlineatus*	XP_021584378	TSPSVIYRPHPSALCSS IQANALDVSDLPTQ
	Marmota marmota marmota	XP_015338506	----------------- --------------
	Urocitellus parryii	XP_026242468	----------------- --------------
	Marmota monax	VTJ67622	----------------- --------------
	Marmota flaviventris	XP_027784182	----------------- --------------
**Other *Euarchontoglires***	*Rattus norvegicus*	Q62739	------------T---A T ------NL------
	Rattus rattus	XP_032768608	------------T---A T ------NL------
	Mus pahari	XP_021060592	------------T--AA P L------L------
	Mus musculus	NP_001003950	------------T--AA P L------L------
	Meriones unguiculatus	XP_021499311	------------S--TA P V--------N----
	Mastomys coucha	XP_031205131	------------T--AA P -------L-A----
	Mus caroli	XP_021029547	------------T--AA P L------L------
	Grammomys surdaster	XP_028614908	-------------F-AA P V-----NL------
	Cricetulus griseus	XP_016834172	------------S---T P L------L------
	Microtus ochrogaster	XP_013205852	------------S---A P L------L------
	Peromyscus maniculatus bairdii	XP_015862875	------------S---A P L------L------
	Peromyscus leucopus	XP_028733772	------------S---A P L------L------
	Mesocricetus auratus	XP_012977003	------------S---T P L------L------
	Onychomys torridus	XP_036025904	------------S--PA P L------L------
	Nannospalax galili	XP_008821926	------------S---A P ---------H----
	Jaculus jaculus	XP_004650147	-------W----S---A P --------------
	Dipodomys ordii	XP_012878000	--- -------------T P --------------
	Castor canadensis	XP_020022889	----------------A P V-------------
	Chinchilla lanigera	XP_013372994	----------------V P V-------------
	Cavia porcellus	XP_023423416	----------------A P V------A------
	Octodon degus	XP_023572073	-----------A---PA P V-------------
	Heterocephalus glaber	XP_004845110	--A---F---------T P V-------------
	Fukomys damarensis	XP_019066193	--A---------N---T P V-------------
	Ochotona princeps	XP_004583079	---------Y------A P -------A------
	Oryctolagus cuniculus	XP_008255001	---G-------A----A P -------A------
	Tupaia chinensis	XP_006146235	----------------A H --------------
	Galeopterus variegatus	XP_008586792	---G------------A P --------------
	Sapajus apella	XP_032151389	----------------V P ---------E----
	Propithecus coquereli	XP_012497471	----------------T P ---------E----
	Callithrix jacchus	XP_009002452	----------------V P ---------E----
	Saimiri boliviensis boliviensi	XP_010336730	----------------V P ---------E----
	Homo sapiens	AAH59358	---------------S-V P ---------E----
	Gorilla gorilla gorilla	XP_004053606	---------------S-V P ---------E----
	Hylobates moloch	XP_032026612	---------------S-V P ---------E----
	Carlito syrichta	XP_008064807	----------------A P ---------E----
	Nomascus leucogenys	XP_030675859	---------------S-V P ---------E----
	Pan paniscus	XP_008959028	---------------S-V P ---------E----
	Aotus nancymaae	XP_012296773	----------------V P ---------E----
	Microcebus murinus	XP_012644186	----------------T P ---------E----
	Macaca fascicularis	XP_015286681	---------------S-V P ---------E----
	Papio anubis	XP_031506479	---------------S-V P ---------E----
	Chlorocebus sabaeus	XP_008002191	---------------S-V P ---------E----
	Cercocebus atys	XP_011905071	---------------S-V P ---------E----
	Pan troglodytes	XP_024203713	---------------S-V P ---------E----
	Macaca mulatta	XP_028685553	---------------S-V P ---------E----
	Pongo abelii	XP_009246287	---------------SFV P ---------E----
	Rhinopithecus roxellana	XP_030795465	---------------S-V P ---------E----
	Rhinopithecus bieti	XP_017749552	---------------S-V P ---------E----
	Trachypithecus francoisi	XP_033077181	---------------S-V P ---------E----
	Otolemur garnettii	XP_023373692	---------Q------T A L---T----E----
	Colobus angolensis palliatus	XP_011784705	---------------S-A P ---------E----
	Piliocolobus tephrosceles	XP_026304235	---------------S-V P ---------E----
	Macaca nemestrina	XP_011725483	---------------S-V P ---------E----

Supplementary figure S25: Multiple sequence alignment of a conserved segment of the protein rab-3A-interacting protein isoform 2, showing a 1 amino acid deletion that is present in the suborder Sciuromorpha. This CSI was detected in all species within Sciuromorpha, and not detected in any other members of the Euarchontoglires in the top 250 BLASTp returns. The dashes (-) within the alignment indicate the same amino acid as the top line. The second column indicates accession numbers for each of the sequences and the position of the partial sequence is shown by the numbers at the top.

Group	Species	Accession	335	Insert	370
Sciuromorpha (5/5)	*Ictidomys tridecemlineatus*	XP_021579115	GDYFRQKLRGQENGAHR	TA	AAFSRLLSGVDDISINGVEN
	Urocitellus parryii	XP_026236274	-----------------	--	--------------------
	Marmota marmota marmota	XP_015359450	-----------K-----	--	--------------------
	Marmota monax	VTJ74178	-----------K-----	--	--------------------
	Marmota flaviventris	XP_027811625	-----------K-----	--	--------------------
Other Euarchontoglires(1/49)	*Rattus norvegicus*	XP_038951756	---------------L-		E---KH--D--------L--
	Grammomys surdaster	XP_028637055	---------------PL-		E---KH--D---V----L--
	Rattus rattus	XP_032740107	---------------PL-		D---KH--D--------L--
	Mus caroli	XP_021035550	---------------PL-		E---KH--DA--L----L--
	Mus pahari	XP_021070852	---------------PL-		E---KH--DA--L----L--
	Mus musculus	XP_036013890	---------------PL-		E---KH--DA--L-L--L--
	Meriones unguiculatus	XP_021504751	---------RP---SLG	E	E---KH--D--------L--
	Onychomys torridus	XP_036043058	---------------PL-		----KQ--D------D-L--
	Microtus ochrogaster	XP_005371697	---Y-----------PL-		T----Q--DI-----S-L--
	Peromyscus leucopus	XP_037061526	-E-------------PL-		----KQ--D------D-L--
	Cricetulus griseus	XP_027265433	------R--------PLS		--L-KQ--DI-------L--
	Mesocricetus auratus	XP_040612081	---L-----------PL-		----KH--D------S-L--
	Peromyscus maniculatus bairdii	XP_015855768	-E-------------PL-		TT--KQ--D------D-L--
	Jaculus jaculus	XP_004656908	---------------P--		T---Q----------S----
	Castor canadensis	XP_020014490	-E-------------P--		T---K--L--------E---
	Dipodomys ordii	XP_012873909	-E--H----E-----P-G		----K----------S----
	Chinchilla lanigera	XP_013369536	---------------P--		T-------------------
	Fukomys damarensis	XP_010605924	---------------P--		----K---------------
	Heterocephalus glaber	EHB09648	---------------P--		T---K---------------
	Octodon degus	XP_004643859	--C------------P--		T---K---------------
	Oryctolagus cuniculus	XP_017197747	---------------Q--		TSL-----------------
	Tupaia chinensis	XP_027631646	---------V--S-Q--		----K--L----------K-
	Rhinopithecus roxellana	XP_030775731	--------K-----Q--		----K---------------
	Otolemur garnettii	XP_023363881	--------K-----Q--		--------A-----------
	Homo sapiens	EAW60986	--------K-----Q--		----K---------------
	Papio anubis	XP_021783438	--------K-----Q--		----K---------------
	Macaca mulatta	XP_014989052	--------K-----Q--		----K---------------
	Pan paniscus	XP_008961144	--------K-----Q--		----K---------------
	Pan troglodytes	XP_024213599	--------K-----Q--		----K---------------
	Macaca nemestrina	XP_011757685	--------K-----Q--		----K---------------
	Macaca fascicularis	XP_005549673	--------K-----Q--		----K---------------
	Chlorocebus sabaeus	XP_007979545	--------K-----Q--		----K---------------
	Propithecus coquereli	XP_012495507	--------------Q-G		------------------R-
	Cercocebus atys	XP_011901422	--------K-----Q--		----K---------------
	Rhinopithecus bieti	XP_017726981	--------K-----Q--		----K---------------
	Pongo abelii	XP_024108321	--------K-----Q--		----K---------------
	Callithrix jacchus	XP_035156291	--------------Q-G		------------------K-
	Sapajus apella	XP_032122274	--------K-----Q--		----K-------------K-
	Gorilla gorilla gorilla	XP_030870485	--------K-----Q--		----K-------V-------
	Trachypithecus francoisi	XP_033092547	--------K-----Q--		----K---------------
	Theropithecus gelada	XP_025216057	--------K-----Q--		----K---------------
	Piliocolobus tephrosceles	XP_026302611	--------K-----Q--		----K---------------
	Hylobates moloch	XP_032020501	--------K-----Q--		----K--------------H
	Nomascus leucogenys	XP_030654455	--------K-----Q--		----K--------------H
	Carlito syrichta	XP_008053951	--------------Q-G		----K----------H----
	Galeopterus variegatus	XP_008568027	--------------Q--		SS-----L----------K-
	Microcebus murinus	XP_012642689	----H---------Q-G		------------------R-
	Aotus nancymaae	XP_021532788	---------V----Q-G		----K-------------K-
	Saimiri boliviensis boliiensis	XP_039330959	---------RP---Q-G		----K-------------K-

Supplementary figure S26: Multiple sequence alignment of a conserved segment of the protein dual specificity protein phosphatase CDC14B, showing a 2 amino acid insertion that is present in the suborder Sciuromorpha. This CSI was detected in all species within Sciuromorpha, and only detected in 1 other member of the Euarchontoglires in the top 250 BLASTp returns. The dashes (-) within the alignment indicate the same amino acid as the top line. The second column indicates accession numbers for each of the sequences and the position of the partial sequence is shown by the numbers at the top.

			7		44
**Sciuromorpha (4/4)**	*Ictidomys tridecemlineatus*	XP_013213978	LKQILPFPLEPAPTLGLF		NYSTMDPVQKAVLSHTFG
	Marmota marmota marmota	XP_015349059	------------------		------------------
	Urocitellus parryii	XP_026254561	------------------		------------------
	Marmota flaviventris	XP_027805816	------------------		------------------
**Other *Euarchontoglires***	*Mastomys coucha*	XP_031213348	------------------	S	------------------
	Meriones unguiculatus	XP_021507767	------------------	S	------------------
	Arvicanthis niloticus	XP_034372501	------------------	S	------------------
	Rattus norvegicus	NP_001102940	------------------	S	------------------
	Grammomys surdaster	XP_028636960	------------------	S	------------------
	Mus musculus	NP_038894	------------------	S	------------------
	Mus caroli	XP_021039485	------------------	S	------------------
	Cricetulus griseus	XP_027252581	------------------	S	------------------
	Microtus ochrogaster	XP_013204159	------------------	S	------------------
	Peromyscus maniculatus bairdii	XP_006981858	------------------	S	------------------
	Peromyscus leucopus	XP_028730236	------------------	S	------------------
	Onychomys torridus	XP_036063644	------------------	S	------------------
	Mesocricetus auratus	XP_005067408	------------------	S	------------------
	Jaculus jaculus	XP_004649985	------------------	S	---A--------------
	Castor canadensis	XP_020019656	------------------	G	------------------
	Dipodomys ordii	XP_012887921	------------------	G	------------------
	Chinchilla lanigera	XP_005397280	------------------	G	------------------
	Octodon degus	XP_023578642	------------------	G	------------------
	Cavia porcellus	XP_005006485	------------------	G	------------------
	Heterocephalus glaber	EHB09045	------------------	G	------------------
	Fukomys damarensis	XP_010607686	------------------	S	------------------
	Oryctolagus cuniculus	XP_008254800	------------------	S	------------------
	Ochotona princeps	XP_004583203	-------------A----	S	------------------
	Pan troglodytes	PNI89927	------------------	S	------------------
	Pongo abelii	PNJ45030	------------------	S	------------------
	Macaca fascicularis	EHH66342	------------------	S	------------------
	Macaca mulatta	EHH20805	------------------	S	------------------
	Homo sapiens	NP_001276930	------------------	S	------------------
	Galeopterus variegatus	XP_008590954	------------------	S	------------------
	Rhinopithecus bieti	XP_017706424	------------------	S	------------------
	Macaca nemestrina	XP_011727019	------------------	S	------------------
	Aotus nancymaae	XP_012305795	------------------	S	------------------
	Colobus angolensis palliatus	XP_011785834	------------------	S	------------------
	Gorilla gorilla gorilla	XP_018893402	------------------	S	------------------
	Pan paniscus	XP_003807619	------------------	S	------------------
	Saimiri boliviensis boliviensi	XP_010348223	------------------	G	------------------
	Rhinopithecus roxellana	XP_010372160	------------------	S	------------------
	Papio anubis	XP_003906548	------------------	S	------------------
	Chlorocebus sabaeus	XP_008001710	--------I---------	S	------------------
	Otolemur garnettii	XP_003790510	------------------	S	--N---------------
	Propithecus coquereli	XP_012507969	------------------	S	--N---------------
	Rhinopithecus roxellana	XP_010372160	------------------	S	------------------
	Sapajus apella	XP_032147680	------------------	S	------------------
	Cercocebus atys	XP_011903348	------------------	S	------------------
	Hylobates moloch	XP_032029390	------------------	S	------------------
	Trachypithecus francoisi	XP_033077698	------------------	S	------------------
	Nomascus leucogenys	XP_030678364	------------------	G	------------------
	Callithrix jacchus	XP_002752602	------------------	S	---A--------------

Supplementary figure S27: Multiple sequence alignment of a conserved segment of the protein zinc finger protein 385A, showing a 1 amino acid deletion that is present in the suborder Sciuromorpha. This CSI was detected in all species within Sciuromorpha, and not detected in any other members of the Euarchontoglires in the top 250 BLASTp returns. The dashes (-) within the alignment indicate the same amino acid as the top line. The second column indicates accession numbers for each of the sequences and the position of the partial sequence is shown by the numbers at the top.

Group	Species	Accession	336	Insert	371
**Sciuromorpha (5/5)**	*Ictidomys tridecemlineatus*	XP_021590074	NLEITWYPFDVEDTTPSS	G	GAGNKTAALQRRMSMYSQ
	Marmota flaviventris	XP_027803908	------------------	-	-----A------------
	Marmota monax	VTJ51869	------------------	-	------------------
	Urocitellus parryii	XP_026263491	------------------	-	------------------
	Marmota marmota marmota	XP_015349568	------------------	-	------------------
**Other *Euarchontoglires***	*Rattus norvegicus*	EDL86515	------------------		-P----------------
	Arvicanthis niloticus	XP_034366716	------------------		-S----------------
	Mus musculus	BAE37527	------------------		-P----------------
	Mus pahari	XP_029403412	------------------		-P----------------
	Grammomys surdaster	XP_028632129	-----------------A		-S----------------
	Meriones unguiculatus	XP_021497075	------------------		-P----------------
	Mastomys coucha	XP_031214982	------------------		-P----------------
	Rattus rattus	XP_032741201	------------------		-P----------------
	Mus caroli	XP_021035845	------------------		-P----T-----------
	Cricetulus griseus	ERE77269	------------------		-P----------------
	Peromyscus maniculatus bairdii	XP_015851458	------------------		-P----------------
	Peromyscus leucopus	XP_028736495	------------------		-P----------------
	Onychomys torridus	XP_036043797	------------------		-P----------------
	Mesocricetus auratus	XP_021080716	------------------		-P------I---------
	Microtus ochrogaster	XP_005355029	------------------		-P----------------
	Nannospalax galili	XP_008845309	------------------		-----A------------
	Jaculus jaculus	XP_012806996	------------------		-----A------------
	Dipodomys ordii	XP_012890704	------------------		-T----------------
	Castor canadensis	XP_020028640	------------------		-----A------------
	Cavia porcellus	XP_013006861	-------------M----		-----A------------
	Octodon degus	XP_023555747	-------------M----		-----A------------
	Fukomys damarensis	XP_033617778	-------------M----		-----AV-----------
	Ochotona princeps	XP_012784640	---------------S--		--A------H--------
	Microcebus murinus	XP_012596795	------------------		-----A------------
	Callithrix jacchus	XP_035150646	------------------		-----A------------
	Sapajus apella	XP_032103494	------------------		-----A------------
	Carlito syrichta	XP_008057582	------------------		-----A------------
	Otolemur garnettii	XP_012659695	------------------		-----A------------
	Propithecus coquereli	XP_012494128	------------------		-G---A------------
	Aotus nancymaae	XP_021523321	------------------		-----A------------
	Saimiri boliviensis boliviensis	XP_010336306	------------------		-----A------------
	Callithrix jacchus	XP_035150635	------------------		-----A------------
	Homo sapiens	XP_006715338	-------------M-A--		-----A------------
	Pan troglodytes	XP_009448876	-------------M-A--		-----A------------
	Chlorocebus sabaeus	XP_037861693	-------------M-A--		-----A------------
	Papio anubis	XP_031522821	-------------M-A--		-----A------------
	Rhinopithecus roxellana	XP_010363480	-------------M-A--		-----A------------
	Piliocolobus tephrosceles	XP_023065499	-------------M-A--		-----A------------
	Theropithecus gelada	XP_025238673	-------------M-A--		-----A------------
	Trachypithecus francoisi	XP_033072291	-------------M-A--		-----A------------
	Macaca mulatta	XP_014991167	-------------M-A--		-----A------------
	Gorilla gorilla gorilla	XP_030868091	-------------M-A--		-----A------------
	Pongo abelii	XP_024104503	-------------M-A--		-----A------------
	Hylobates moloch	XP_031996253	-------------M-T--		-----A------------
	Nomascus leucogenys	XP_030673247	-------------M-T--		-----A------------
	Pan paniscus	XP_003823254	----A--------M-A--		-----A------------
	Macaca nemestrina	XP_011740990	------C------M-T--		-----A------------

Supplementary figure S28: Multiple sequence alignment of a conserved segment of the protein rho family-interacting cell polarization regulator 2, showing a 1 amino acid insertion that is present in the suborder Sciuromorpha. This CSI was detected in all species within Sciuromorpha, and not detected in any other members of the Euarchontoglires in the top 250 BLASTp returns. The dashes (-) within the alignment indicate the same amino acid as the top line. The second column indicates accession numbers for each of the sequences and the position of the partial sequence is shown by the numbers at the top.

Group	Species	Accession	587		625
***Sciuromorpha* (5/5)**	*Marmota flaviventris*	XP_027803906	PAATERLLSEDSASGES		FLDGSLEDALSGLFLAL
	Marmota marmota marmota	XP_015349568	-----------------		-----------------
	Ictidomys tridecemlineatus	XP_021590076	-----------------		-----------------
	Marmota monax	VTJ51869	-----H-----------		L----------------
	Urocitellus parryii	XP_026263491	-------------G---		-----------------
**Other *Euarchontoglires***	*Mastomys coucha*	XP_031214979	-V--DK-FF-G-VGS--	EAGRS	---------FN------
	Mus musculus	XP_006516650	-VT-DK-FF-G-VGS--	EAGRS	---------FN------
	Mus caroli	XP_029324299	-IT-DK-FF-G-VGS--	EAGRS	---------FN------
	Rattus norvegicus	XP_017456019	-IT-NK-FF-G-VGS--	EAGRS	---------FN------
	Rattus rattus	XP_032741200	-IT-NK-FF-G-VGS--	EAGRS	---------FN------
	Arvicanthis niloticus	XP_034366715	-IT-DK-FF-G-VGS--	EAGRS	---------F-------
	Grammomys surdaster	XP_028632128	-IT-DK-FF-G-VGS--	EAGRS	---------F-------
	Mus pahari	XP_021070973	-IT-DK-F--G-VGS--	EAGRS	---------FN------
	Microtus ochrogaster	XP_005355026	-I---K-FF-G-VGS--	EAGRS	---------FN------
	Cricetulus griseus	XP_035297406	-IV--K-FF-G-VGS--	EAGRS	---------FN------
	Mesocricetus auratus	XP_040611653	-I---K-CF-G-VGS--	EAGRS	---------FN------
	Peromyscus leucopus	XP_037061762	-I---K-VF-G-VGS--	EAGRS	---------FH------
	Onychomys torridus	XP_036043791	-IP--K-VF-G-VGS--	EAGRS	---------FH------
	Nannospalax galili	XP_008845305	-VTS----FDGPV-S--	EAAQS	---------FN------
	Castor canadensis	XP_020028640	-V------F-G-V----	ESCRS	---------FN------
	Cavia porcellus	XP_013006861	------Q-Y-G-V----	EGCRS	---------F-------
	Chinchilla lanigera	XP_005403412	---A-GQ-C-G-V----	EGCRS	---------F-------
	Octodon Degus	XP_023555747	---S--Q-Y-G-V----	EGCRS	---------F-------
	Fukomys damarensis	XP_033617778	---G--Q-Y-G-V----	EGCRS	---------F-------
	Carlito syrichta	XP_008057582	--------F-G-V----	EGCRS	---------FN------
	Microcebus murinus	XP_012596795	--------F-G-V----	EGCRS	---------FN------
	Otolemur garnettii	XP_012659695	-V------F-G-V----	EGCRS	---------FN------
	Pongo abelii	XP_002816537	-V--D-----G-VG---	EGCRS	---------FN------
	Pan troglodytes	XP_009448881	-M--D-----G-VG---	EGCRS	---------FN------
	Callithrix jacchus	XP_035150643	-V--D-----G-VG---	EGCRS	---------FN------
	Pan paniscus	XP_003823254	-M--D-----G-VG---	EGCRS	---------FN------
	Gorilla gorilla gorilla	XP_030868091	-M--D-----G-VG---	EGCRS	---------FN------
	Nomascus leucogenys	XP_030673247	-V--D-----G-VG---	EGCRS	---------FN------
	Hylobates moloch	XP_031996253	-V--D-----G-VG---	EGCRS	---------FN------
	Callithrix jacchus	XP_035150635	-V--D-----G-VG---	EGCRS	---------FN------
	Theropithecus gelada	XP_025238673	-V--D-V---G-VG---	EGCRS	---------FN------
	Macaca nemestrina	XP_011740990	-V--D-V---G-VG---	EGCRS	---------FN------
	Chlorocebus sabaeus	XP_037861693	-V--D-V---G-VG---	EGCRS	---------FN------
	Papio anubis	XP_031522821	-V--D-V---G-VG---	EGCRS	---------FN------
	Macaca mulatta	XP_014991167	-V--D-V---G-VG---	EGCRS	---------FN------
	Piliocolobus tephrosceles	XP_023065499	-V--D-V---G-VG---	EGCRS	---------FN------
	Rhinopithecus roxellana	XP_010363480	-V--D-V---G-VG---	EGCRS	---------FN------
	Trachypithecus francoisi	XP_033072291	-V--D-V---G-VG---	EGCRS	---------FN------
	Homo sapiens	XP_006715338	-M--D-----G-VG---	EGCRS	---------FN--L---
	Sapajus apella	XP_032103486	-V-PD-----G-VG--P	EGCRS	---------FN------
	Saimiri boliviensis boliviensis	XP_010336306	-V-PD-----G-VG--P	EGCRS	---------FN------

Supplementary figure S29: Multiple sequence alignment of a conserved segment of the protein rho family-interacting cell polarization regulator 2, showing a 5 amino acid deletion that is present in the suborder Sciuromorpha. This CSI was detected in all species within Sciuromorpha, and not detected in any other members of the Euarchontoglires in the top 250 BLASTp returns. The dashes (-) within the alignment indicate the same amino acid as the top line. The second column indicates accession numbers for each of the sequences and the position of the partial sequence is shown by the numbers at the top.

Group	Species	Accession	262		289
**_Myomorpha (18/19)_**	*Mus musculus*	XP_006524473	QRSKREK		NGTGEDKPKRKRQKIQTSCPF
	Arvicanthis niloticus	XP_034380878	----H--		-R--------R----------
	Grammomys surdaster	XP_028633808	----H--		-R--------R----------
	Mus caroli	XP_021041364	-------		-------A-------------
	Mus pahari	XP_021073619	-------		-R-------------------
	Meriones unguiculatus	XP_021483219	--I-H--		SRP-G-----R----------
	Mastomys coucha	XP_031224478	-------		-M-R-------T----A----
	Rattus norvegicus	XP_008765629	--R-H--		MRI-------R---M-A----
	Rattus rattus	XP_032757347	--R-H--		IRI----A--R---M-A----
	Mesocricetus auratus	XP_012973887	----H--		--AE--R--KR-R-V------
	Onychomys torridus	XP_036029614	----H--		--A-------R----------
	Peromyscus leucopus	XP_037066075	----H--		--A-------R-T--------
	Cricetulus griseus	ERE83173	----H--		T-A---M--KR-RR-------
	Microtus ochrogaster	XP_026642320	----H--		--AV---S----R--H-----
	Neotoma lepida	OBS80128	----H--		--A----X--R-R--------
	Peromyscus maniculatus bairdii	XP_015846187	---RH--		--A-------R-T--------
	Arvicola amphibius	XP_038196402	----H--		--AV---S----R--------
	Nannospalax galili	XP_029420171	----H--		KRVRD--S-KR--ET-M----
	Jaculus jaculus	XP_012803803	----H--	K	-RAE--R--KR--ES-AT---
**_Castorimorpha (2/2)_**	*Castor canadensis*	XP_020036107	--N-H--		SRAE-N----R--ESRAA---
	*Dipodomys ordii***	XP_012879066	----H-N		HRAEP-Q---R-PGGRAA---
**_Hystricomorpha (0/4)_**	*Heterocephalus glaber*	EHB05252	---QHKS	K	SRAE-E----R--EARAA---
	Chinchilla lanigera	XP_013362757	---RP-S	K	S-AEA-E---R-REGRAA---
	Cavia porcellus	XP_013006403	---PH-N	K	-RAEAE----R--EGR-A---
	Octodon degus	XP_004646958	--HQY-S	K	HRAEA-M---R--ESLAA---
**_Sciuromorpha (0/3)_**	*Ictidomys tridecemlineatus*	XP_005334016	----H--	K	-RAE-----KR--ETKAA---
	Marmota flaviventris	XP_027806874	----H--	K	-RAE-----KR--ETKAA---
	Marmota marmota marmota	XP_015356293	----H--	K	-RAE-----KR--ETKAA---
**Other _Euarchontoglires (1/31)_**	*Oryctolagus cuniculus*	XP_017199094	----H--	K	-KAE-EQ---R-RESRAA---
	Ochotona princeps	XP_004596500	----H--	K	SKAEKGQ---R-RESRAA---
	Tupaia chinensis	XP_027632392	---RLGR	K	DEAE-E----R-REAGPA---
	Colobus angolensis palliatus	XP_011808637	---RH--	K	K-AE-ER---R--EK-AA---
	Chlorocebus sabaeus	XP_007965744	---RH-N	K	K-AE-E----R--EK-AA---
	Microcebus murinus	XP_012634895	----H--	R	-EAE-E----R--EKRA----
	Propithecus coquereli	XP_012516720	----H--	R	-EAE-E----R--EKRA----
	Otolemur garnettii	XP_023369833	----H--	R	-EAE-E----R--EK-AA---
	Homo sapiens	XP_011518899	---RH--	K	K-AE-E----R--EK-AA---
	Rhinopithecus bieti	XP_017749761	---RH--	K	K-AE-E----R--EK-AA---
	Pan paniscus	XP_034791980	---RH--	K	K-AE-E----R--EK-AA---
	Nomascus leucogenys	XP_030660502	---RH--	K	K-AE-E----R--EK-AA---
	Pan troglodytes	XP_016778421	---RH--	K	K-AE-E----R--EK-AA---
	Gorilla gorilla gorilla	XP_030856791	---RH--	K	K-AE-E----R--EK-AA---
	Pongo abelii	PNJ30631	---RH--		K-AE-E----R--EK-VA---
	Rhinopithecus roxellana	XP_030794764	---RH--	K	K-AE-E----R--EK-AA---
	Macaca mulatta	XP_001114510	---RH--	K	K-AE-E----R--EK-AA---
	Theropithecus gelada	XP_025258352	---RH--	K	K-AE-E----R--EK-AA---
	Macaca fascicularis	EHH66054	---RH--	K	K-AE-E----R--EK-AA---
	Hylobates moloch	XP_032015471	---RH--	K	K-AE-E----R--EK-AA---
	Cercocebus atys	XP_011920118	---RH--	K	K-AE-E----R--EK-AA---
	Papio anubis	XP_021777710	---RH--	K	K-AE-E----R--EK-AA---
	Carlito syrichta	XP_021565713	----H--	K	H-AEQE----R--EK-AA---
	*Saimiri boliviensis boliviensis**	XP_010331258	---RHGE	K	K-AE-E----R--EK-AA---
	Galeopterus variegatus	XP_008576029	----H--	K	DKAE-E----R--EKRAA---
	Aotus nancymaae	XP_012328189	---RH--	K	K-AE-E----R--EK-AA---
	Macaca nemestrina	XP_024642869	---RH--	K	K-AE-E----R--EK-AA---
	Sapajus apella	XP_032110583	---RH--	K	K-AE-E----R--EK-AA---
	Trachypithecus francoisi	XP_033079286	---RH--	K	K-AE-E--E-R--EK-AA---
	Callithrix jacchus	XP_035111494	---RH--	K	K-AE-E----R-PEK-AA---
	Piliocolobus tephrosceles	XP_023048110	---RP-E	K	K-AE-E----R--EK-AA---

*Has SHRDHGIAA after site 267
**Has RAS after site 287

Supplementary figure S30: Multiple sequence alignment of a conserved segment of the protein ATP-dependent DNA helicase DDX11 isoform 1, showing a 1 amino acid deletion that is present in the suborders Myomorpha and Castorimorpha. This CSI was detected in all species within Myomorpha and Castorimorpha, except for 1, and only detected in 1 other member of the Euarchontoglires in the top 250 BLASTp returns. The dashes (-) within the alignment indicate the same amino acid as the top line. The second column indicates accession numbers for each of the sequences and the position of the partial sequence is shown by the numbers at the top.

375 **401**

Group	Species	Accession	Sequence	Insert	Sequence
**Myomorpha (18/19)**	*Rattus norvegicus*	XP_006254360	QMVAADKLAQCPP	Q	ESFDVILDENQLED
	Mus musculus	XP_006497377	-------------	-	--------------
	Arvicanthis niloticus	XP_034365587	-------------	-	--------------
	Mastomys coucha	XP_031224921	-------------	-	--------------
	Grammomys surdaster	XP_028620661	-------------	-	--------------
	Mus pahari	XP_021049074	-------------	-	--------------
	Mus caroli	XP_021012451	-------------	-	--------------
	Rattus rattus	XP_032740069	-------------	-	--------------
	Mastomys coucha	XP_031224918	-------------	-	--------------
	Meriones unguiculatus	XP_021484107	-------------		--------------
	Onychomys torridus	XP_036043106	-------------	-	--------------
	Cricetulus griseus	ERE79349	-------------	-	--------------
	Peromyscus maniculatus bairdii	XP_006989996	-------------	-	--------------
	Arvicola amphibius	XP_034365587	-------------	-	--------------
	Mesocricetus auratus	XP_021089306	-------------	-	--------------
	Microtus ochrogaster	XP_005354699	-------------	-	--------------
	Peromyscus leucopus	XP_028726335	-------------	-	--------------
	Jaculus jaculus	XP_004668460	-------------	-	--------------
	Nannospalax galili	XP_008822339	-------------	-	--------------
**Castorimorpha (1/2)**	*Dipodomys ordii*	XP_012870156	-------------	-	--------------
	Castor canadensis	XP_020041119	-------------		--------------
**Other Euarchontoglires**	*Cavia porcellus*	XP_012999994	-------------		-L------------
	Fukomys damarensis	KFO21627	-------------		-L------------
	Heterocephalus glaber	EHB07999	-------------		-L------------
	Octodon degus	XP_004860011	-L-----------		-M------------
	Chinchilla lanigera	XP_013370891	-------------		-L------------
	Urocitellus parryii	XP_026263513	-------------		-L------------
	Marmota flaviventris	XP_027784330	-------------		-L------------
	Ictidomys tridecemlineatus	XP_040134277	-------------		-L------------
	Marmota marmota marmota	XP_015338884	-------------		-L------------
	Oryctolagus cuniculus	1T3L_A	-------------		-L------------
	*Ochotona princeps**	XP_004578620	-------------		-L------------
	Pan troglodytes	BAK62103	-------------		-L------------
	Homo sapiens	AAV49501	-------------		-L------------
	Piliocolobus tephrosceles	XP_023078909	-------------		-L------------
	Trachypithecus francoisi	XP_033065321	-------------		-L------------
	Colobus angolensis palliatus	XP_011819262	-------------		-L------------
	Rhinopithecus bieti	XP_017724702	-------------		-L------------
	Pongo abelii	XP_024108784	-------------		-L------------
	Microcebus murinus	XP_012604108	-------------		-L------------
	Galeopterus variegatus	XP_008568035	-------------		-L------------
	Aotus nancymaae	XP_012320882	-------------		-L------------
	Chlorocebus sabaeus	XP_008000615	-------------		-L------------
	Macaca nemestrina	XP_011750424	-------------		-L------------
	Papio anubis	XP_031508328	-------------		-L------------
	Otolemur garnettii	XP_003786824	-------------		-L------------
	Saimiri boliviensis boliviensis	XP_003922026	-L-----------		-M------------
	Rhinopithecus roxellana	XP_010371204	-L-----------		-M------------
	Callithrix jacchus	XP_017829576	-------------		-L------------
	Sapajus apella	XP_032099827	-------------		-L------------
	Propithecus coquereli	XP_012516396	-------------		-L------------
	Macaca mulatta	NP_001244711	-------------		-L------------
	Hylobates moloch	XP_032021813	-------------		-L------------
	Nomascus leucogenys	XP_030652692	-L-----------		-M------------
	Carlito syrichta	XP_008053438	-------------		-L------------
	Gorilla gorilla gorilla	XP_004049162	-------------		-L------------

*Has CLSLQ after site 387

Supplementary figure S31: Multiple sequence alignment of a conserved segment of the protein voltage-dependent L-type calcium channel subunit beta-2, showing a 1 amino acid insertion that is present in the suborders Myomorpha and Castorimorpha. This CSI was detected in all species within Myomorpha and Castorimorpha except for 2, and not detected in any other members of the Euarchontoglires in the top 250 BLASTp returns. The dashes (-) within the alignment indicate the same amino acid as the top line. The second column indicates accession numbers for each of the sequences and the position of the partial sequence is shown by the numbers at the top.

			232		263
Muridae (9/9)	*Rattus norvegicus*	58865646	KNPVFAGVRLPEIK	DI	EAEPLESRYPKLPEVV
	Mus musculus	74177560	------------V-	-A	--------------A-
	Meriones unguiculatus	XP_021504310	--------------	-T	-T--------------
	Arvicanthis niloticus	XP_034364943	--------------	-T	----------------
	Grammomys surdaster	XP_028608446	--------------	-T	----------------
	Rattus rattus	XP_032771916	--------------	--	----------------
	Mus caroli	XP_021018902	------------V-	-A	----------------
	Mus pahari	XP_021066408	------------V-	-A	----------E-----
	Mastomys coucha	XP_031246845	--------------	-L	--------F-N-----
Cricetidae (7/7)	*Peromyscus maniculatus bairdii*	589962006	--------------	-T	-T--------------
	Mesocricetus auratus	524927979	--------------	-T	-T----N---------
	Microtus ochrogaster	532033272	--------------	-T	-T----C---------
	Cricetulus griseus	625274474	--------------	-T	-T----I---------
	Peromyscus leucopus	XP_028737737	--------------	-T	-T--------------
	Onychomys torridus	XP_036057056	--------------	-T	-T--------------
	Arvicola amphibius	XP_038195363	--------------	-T	-T----C---------
Other *Rodents* (0/14)	*Nannospalax galili*	674069292	------R-------		-T--------------
	Jaculus jaculus	847031168	------------V-		-I----K---------
	Dipodomys ordii	852760512	-------G------		-T----R---------
	Castor canadensis	XP_020038491	--------------		-TA---K---Q-S---
	Chinchilla lanigera	533123140	---A---------Q		-T----R-----S---
	Octodon degus	820966904	---A---A-----Q		-T----R-----S---
	Fukomys damarensis	731280676	-------------Q		-T----R-----SQ--
	Cavia porcellus	348567268	---A-T-------Q		-T----R-----S---
	Heterocephalus glaber	351700156	--------Q----Q		-K----R-----S---
	Ictidomys tridecemlineatus	532095232	--------------		-T----R-----S---
	Urocitellus parryii	XP_026264254	--------------		-T----R-----S---
	Marmota flaviventris	XP_027795614	--------------		-T----R-----SD--
	Marmota marmota marmota	XP_015354779	--------------		-T----R-----SD--
	Marmota monax	KAF7480154	--------------		-T----R-----SD--
Other *Euarchontoglires*	*Oryctolagus cuniculus*	655856638	--------------		-S----R-----S---
	Ochotona princeps	504173333	--------------		-T----R-----S---
	Tupaia chinensis	444730085	--------T-----		-M----K-----S---
	Propithecus coquereli	826337693	--------------		-T----R------D--
	Pan paniscus	675800575	--------------		-R----R-----S---
	Cercocebus atys	795495555	--------------		-R----R-----S---
	Pan troglodytes	694907555	--------------		-R----R-----S---
	Gorilla gorilla gorilla	426344684	--------------		-R----R-----S---
	Rhinopithecus roxellana	724892816	--------------		-R----R-----S---
	Macaca nemestrina	795624107	--------------		-R----R-----S---
	Pongo abelii	197099074	--------------		-R----R-----S---
	Mandrillus leucophaeus	795292170	--------------		-R----R-----S---
	Macaca fascicularis	544432681	--------------		-R----R-----S---
	Colobus angolensis palliatus	795341339	--------------		-R----R-----S---
	Papio anubis	685541353	--------------		-R----R-----S---
	Macaca mulatta	209862825	--------------		-R----R-----S---
	Otolemur garnettii	395834256	--------------		-TV---K---------
	Chlorocebus sabaeus	635043068	--------------		-RQ---R-----S---
	Microcebus murinus	829741582	--------------		-I----R-----SD--
	Callithrix jacchus	675649054	--------------		-RV---R-----S---
	Aotus nancymaae	817355354	--------------		-RV---R-----S---
	Saimiri boliviensis boliviensi	403281090	--------------		-RV---R-----S---
	Homo sapiens	578809602	--------------		-R----R-----S---
	Galeopterus variegatus	667255440	---M---I------		-M----R-----S---
	Rhinopithecus bieti	XP_017732078	--------------		-R----R-----S---
	Mandrillus leucophaeus	XP_011821566	--------------		-R----R-----S---
	Piliocolobus tephrosceles	XP_026302703	--------------		-R----R-----S---
	Trachypithecus francoisi	XP_033070305	---M----------		-R----R-----S---
	Nomascus leucogenys	XP_030674853	--------------		-R----R-----SK--
	Hylobates moloch	XP_032005902	--------------		-R----R-----SK--
	Sapajus apella	XP_032116912	--------------		-RI---R-----S---

Supplementary figure S32: Multiple sequence alignment of a conserved segment of the protein cyclin-dependent kinase-like 2, showing a 2 amino acid insertion that is present in the families Muridae and Cricetidae. This CSI was detected in all species within Muridae and Cricetidae, and not detected in any other members of the Euarchontoglires in the top 250 BLASTp returns. The dashes (-) within the alignment indicate the same amino acid as the top line. The second column indicates accession numbers for each of the sequences and the position of the partial sequence is shown by the numbers at the top.

Group	Species	Accession	782		817
**_Muridae & Cricetidae_ _(15/15)_**	*Mastomys coucha*	XP_031211290	PQVPRTPAKVPTSAPSLGKP		KSPHSSPTKLPSKSPTK
	Arvicanthis niloticus	XP_034373275	--M-----------------		-----------------
	Rattus norvegicus	NP_001258298	--M-----------------		-----------------
	Rattus rattus	XP_032742247	--M-----------------		-----------------
	Mus musculus	NP_001001884	--M-----------------		-----------------
	Meriones unguiculatus	XP_021490628	--M-----------------		-----------------
	Mus caroli	XP_021039134	--M-----------------		-----------------
	Mus pahari	XP_021072728	--M-----------------		----G------------
	Grammomys surdaster	XP_028644121	--M--------------V--		-----------------
	Peromyscus leucopus	XP_028730442	-------V----P-------		-----------------
	Peromyscus maniculatus bairdii	XP_015848635	-------V----P-------		-----------------
	Microtus ochrogaster	XP_005353973	------------P----N--		-----------------
	Mesocricetus auratus	XP_005067280	--M---------P----S--		----N------------
	Cricetulus griseus	XP_027252344	--M---------PV---S--		----N------------
	Arvicola amphibius	XP_038198837	------------PT---N--		-----------------
**Other _Euarchontoglires_**	*Jaculus jaculus*	XP_004649871	-------V------------	N	-----------------
	Nannospalax galili	XP_008846975	--M--A-----S-G------	N	-----------------
	Dipodomys ordii	XP_012874148	-------------T------	N	-----------------
	Castor canadensis	XP_020025053	-----I--------------	S	-----------------
	Heterocephalus glaber	XP_004863069	--------------------	S	-----------------
	Cavia porcellus	XP_013014851	-----------P--------	S	-----------------
	Chinchilla lanigera	XP_005411906	-----------PA-------	S	R----------------
	Octodon degus	XP_004637848	-----------P-------S	S	-----------------
	Fukomys damarensis	XP_033619435	--------------------	S	-----------------
	Ictidomys tridecemlineatus	XP_005324984	--------------------	N	-----------------
	Urocitellus parryii	XP_026262816	--------------------	N	-----------------
	Marmota marmota marmota	XP_015348814	--------------------	N	-----------------
	Marmota flaviventris	XP_027805547	--------------------	N	-----------------
	Marmota monax	VTJ72549	--------------------	N	-----------------
	Ochotona princeps	XP_004599040	--A--P-----A-V------	S	-----------------
	Tupaia chinensis	ELV10863	--------------------	N	----G------------
	Homo sapiens	XP_005269107	--------------------	N	-----------------
	Macaca nemestrina	XP_011758469	--------------------	N	-----------------
	Rhinopithecus roxellana	XP_030795019	--------------------	N	-----------------
	Macaca mulatta	XP_015007167	--------------------	N	-----------------
	Sapajus apella	XP_032149998	--------------------	N	-----------------
	Trachypithecus francoisi	XP_033078041	--------------------	N	-----------------
	Aotus nancymaae	XP_012296837	--------------------	N	-----------------
	Pongo abelii	XP_024112823	--------------------	N	-----------------
	Cercocebus atys	XP_011903741	--------------------	N	-----------------
	Callithrix jacchus	XP_035112745	--------------------	N	-----------------
	Rhinopithecus bieti	XP_017750218	--------------------	N	-----------------
	Pan paniscus	XP_034791808	--------------------	N	-----------------
	Gorilla gorilla gorilla	XP_018893738	--------------------	N	-----------------
	Pan troglodytes	XP_016778936	--------------------	N	-----------------
	Galeopterus variegatus	XP_008581132	--------------------	N	-----------------
	Papio anubis	XP_009178959	--------------------	N	-----------------
	Piliocolobus tephrosceles	XP_023066951	---------M----------	N	-----------------
	Hylobates moloch	XP_031999793	-------------------S	N	-----------------
	Microcebus murinus	XP_012617211	--A----------------H	N	-----------------
	Otolemur garnettii	XP_012662949	-------------T-----H	N	-----------------
	Chlorocebus sabaeus	XP_008001370	--------------------	N	---Q-------------
	Macaca fascicularis	XP_015286378	--------------------	N	-----------------
	Colobus angolensis palliatus	XP_011791548	--------------------	N	-----------------
	Mandrillus leucophaeus	XP_011820131	--------------------	N	-----------------
	Saimiri boliviensis boliviensis	XP_003939192	--------------------	N	-----------------
	Nomascus leucogenys	XP_030672464	-------------------S	N	-----------------
	Propithecus coquereli	XP_012507809	-------------------H	N	-----------------

Supplementary figure S33: Multiple sequence alignment of a conserved segment of the protein nck-associated protein 5-like, showing a 1 amino acid deletion that is present in the families Muridae and Cricetidae. This CSI was detected in all species within Muridae and Cricetidae, and not detected in any other members of the Euarchontoglires in the top 250 BLASTp returns. The dashes (-) within the alignment indicate the same amino acid as the top line. The second column indicates accession numbers for each of the sequences and the position of the partial sequence is shown by the numbers at the top.

Group	Species	Accession	276		308
**Muridae and Cricetidae (16/16)**	*Mus musculus*	568921788	QRDFISRDLGPALANSSH		DVKLLMLDDQRLLLP
	Rattus norvegicus	189083714	------H-----------		-----I---------
	Meriones unguiculatus	XP_021508871	-----A------------		--Q------------
	Mus caroli	XP_021013365	------------------		---------------
	Mus pahari	XP_029393109	-------------T----		---------------
	Rattus rattus	XP_032753888	------H-----------		-----I---------
	Arvicanthis niloticus	XP_034356303	E----AY------T----		----F----------
	Grammomys surdaster	XP_028608970	E----AY------T----		----F----------
	Microtus ochrogaster	532048862	-----A-------NR---		--Q-F----------
	Mesocricetus auratus	524960545	-----A-------S----		--Q-F----------
	Cricetulus griseus	354478868	-----A-------T----		--Q-F----------
	Peromyscus maniculatus bairdii	589927149	-----A-------T----		---------------
	Mastomys coucha	XP_031230337	------------------		---------------
	Peromyscus leucopus	XP_028746678	-----A-------T----		---------------
	Arvicola amphibius	XP_038167013	-----A-------TH---		--Q-F----------
	Onychomys torridus	XP_036046919	-----A-------T----		----F----------
**Rest of Euarchontoglires**	*Nannospalax galili*	674036932	-----A-----T----T-	H	N-Q------------
	Jaculus jaculus	507567332	-----A-----I----T-	R	-I-------------
	Dipodomys ordii	852746042	-----AH---------T-	R	--L------------
	Castor Canadensis	XP_020021777	-----A------------	R	GIQ------------
	Heterocephalus glaber	351704397	-----A-----T----T-	H	--Q-I----N-----
	Fukomys damarensis	731259387	-----A-----M----T-	H	-IQ-I----N-----
	Chinchilla lanigera	533198003	-----A-----T----T-	R	-IQ-I----N-----
	Cavia porcellus	514471713	-----A-----T----T-	R	-IR-I----N-----
	Octodon degus	XP_004629829	-----A-----T----T-	R	--Q-I----N-----
	Ictidomys tridecemlineatus	532091411	-----A----------T-	R	--Q-F----------
	Urocitellus parryii	XP_026260827	-----A-----T----T-	R	--Q-F----------
	Marmota flaviventris	XP_027776658	-----A-----T----T-	R	--Q-F----------
	Marmota marmota marmota	XP_015358265	-----A-----T----T-	R	--Q-F----------
	Ochotona princeps	504154187	-----A------------	R	--R--I---------
	Oryctolagus cuniculus	655846329	-----A----------T-	R	--R--I---------
	Tupaia chinensis	562849364	-----A-----T----T-	R	N-R------------
	Propithecus coquereli	826334563	-----A----------T-	R	--RVF----------
	Microcebus murinus	829781667	-----A----------T-	R	-IRVF----------
	Papio anubis	685518281	-----A----------T-	H	N-R------------
	Galeopterus variegatus	667291824	-----A-----T----T-	R	N-R------------
	Homo sapiens	193786852	-----A-----T---GT-	H	N-R------------
	Callithrix jacchus	675743744	-----A-----T----T-	R	N-R------------
	Aotus nancymaae	817322016	-----A-----T----T-	R	N-R------------
	Saimiri boliviensis boliviensi	725591860	-----A-----T----T-	R	N-R------------
	Pan paniscus	675742348	-----A-----T----T-	H	N-R------------
	Macaca fascicularis	544401919	-----A-----T----T-	H	N-R------------
	Chlorocebus sabaeus	635104416	-----A-----T----T-	H	N-R------------
	Pan troglodytes	694889189	-----A-----T----T-	H	N-R------------
	Colobus angolensis palliatus	795347462	-----A-----T----T-	H	N-R------------
	Rhinopithecus roxellana	724808059	-----A-----T----T-	H	N-R------------
	Gorilla gorilla gorilla	426331952	-----A-----T----T-	H	N-R------------
	Cercocebus atys	795354654	-----AH----T----T-	H	N-R------------
	Otolemur garnettii	831235099	-----A-----T----T-	R	N-H------N-----
	Nomascus leucogenys	332220593	-----A-----T----T-	H	N-R--V---------
	Pongo abelii	686701909	-----A-----T----T-	H	N-R----N--C----
	Trachypithecus francoisi	XP_033052140	-----A-----T----T-	H	N-R------------
	Mandrillus leucophaeus	XP_011842860	-----A-----T----T-	H	N-R------------
	Macaca mulatta	XP_014966754	-----A-----T----T-	H	N-R------------
	Piliocolobus tephrosceles	XP_026309408	-----A-----T----T-	H	N-R------------
	Sapajus apella	XP_032129097	-----A-----T----T-	R	N-R------------
	Saimiri boliviensis boliviensis	XP_039315823	-----A-----T----T-	R	N-R------------

Supplementary figure S34: Multiple sequence alignment of a conserved segment of the protein lysosomal acid glucosylceramidase, showing a 1 amino acid deletion that is present in the families Muridae and Cricetidae. This CSI was detected in all species within Muridae and Cricetidae, and not detected in any other members of the Euarchontoglires in the top 250 BLASTp returns. The dashes (-) within the alignment indicate the same amino acid as the top line. The second column indicates accession numbers for each of the sequences and the position of the partial sequence is shown by the numbers at the top.

Group	Species	Accession	61		91
Muridae and Cricetidae (16/16)	*Mus musculus*	NP_001104322	ETDDSADSE		VIDSHKRREILSRRPSYRKILN
	Rattus rattus	XP_032740217	---------		----------------------
	Arvicanthis niloticus	XP_034366033	---------		----------------------
	Meriones unguiculatus	XP_021508792	------E--		----------------------
	Rattus norvegicus	XP_038951310	---------		----------------------
	Mus caroli	XP_029328059	---------		----------------------
	Grammomys surdaster	XP_028638493	---------		----------------------
	Mus pahari	XP_029402715	---------		----------------------
	Grammomys surdaster	XP_028638488	---------		----------------------
	Neotoma lepida	OBS78623	------E--		----------------------
	Cricetulus griseus	EGV97836	------E--		----------------------
	Microtus ochrogaster	XP_005354842	---------		----------------------
	Arvicola amphibius	XP_038188538	------E--		----------------------
	Peromyscus maniculatus bairdii	XP_015841254	------E--		----------------------
	Onychomys torridus	XP_036043621	------EP-		----------------------
	Mesocricetus auratus	XP_040611293	------E--		----------------------
**Other *Euarchontoglires***	*Nannospalax galili*	XP_017653867	------E--	G	----------------------
	Jaculus Jaculus	XP_004668220	--NEP-E--	G	---------V------------
	Castor canadensis	XP_020041970	---E--E--	G	----------------------
	Dipodomys ordii	XP_012864839	---E--E--	G	---P------------------
	Chinchilla lanigera	XP_013376904	---E-----	G	------H--V--------R---
	Fukomys damarensis	XP_033621550	-A-E-----	G	----RQH-----------R---
	Hetrocephalus glaber	XP_004861900	---E-----	G	------H-----Q-----R---
	Octodon degus	XP_023557677	---EP----	G	I-----Q-----------R---
	Cavia porcellus	XP_023420430	---E-----	G	------H----------- ---
	Marmota flaviventris	XP_027784322	---E--ET-	G	----------------------
	Marmota marmota marmota	XP_015338922	---E--ET-	G	----------------------
	Urocitellus parryii	XP_026264888	---E--ET-	G	----------------------
	Ictidomys tridecemlineatus	XP_005330903	---E--ET-	G	----------------------
	Marmota monax	KAF7478353	---E--ET-	G	----------------------
	Oryctolagus cuniculus	XP_017206189	---E--E--	G	I---------------------
	Ochotona princeps	XP_012783373	---E-TEP-	G	I---------------------
	Tupaia chinensis	XP_006158380	---E--E--	G	----------------------
	Pongo abelii	XP_009243558	---E--E--	G	----------------------
	Homo sapiens	NP_001254495	---E--E--	G	----------------------
	Nomascus leucogenys	XP_030653511	---E--E--	G	----------------------
	Hylobates moloch	XP_031999309	---E--E--	G	----------------------
	Pan troglodytes	PNI60895	---E--E--	G	----------------------
	Pan paniscus	ABM54241	---E--E--	G	----------------------
	Macaca fascicularis	XP_015311266	---E--E--	G	----------------------
	Callithrix jacchus	XP_008998421	---E--E--	G	----------------------
	Macaca mulatta	XP_028682028	---E--E--	G	----------------------
	Papio anubis	XP_021798870	---E--E--	G	----------------------
	Colobus angolensis palliatus	XP_011811554	---E--E--	G	----------------------
	Cebus imitator	XP_017390825	---E--E--	G	----------------------
	Macaca nemestrina	XP_011764211	---E--E--	G	----------------------
	Sapajus apella	XP_032115643	---E--E--	G	----------------------
	Rhinopithecus roxellana	XP_010387698	---E--E--	G	----------------------
	Piliocolous tephrosceles	XP_023073929	---E--E--	G	----------------------
	Rhinopithecus bieti	XP_017703891	---E--E--	G	----------------------
	Mandrillus leucophaeus	XP_011850524	---E--E--	G	----------------------
	Otolemur garnettii	XP_023367172	---E--E--	G	----------------------
	Aotus nancymaae	XP_012301090	---E--E--	G	----Y-----------------
	Microcebus murinus	XP_012621118	---E--E--	G	----------------------
	Chlorocebus sabaeus	XP_008000988	---E--E--	G	----------------------
	Macaca fascicularis	XP_005565021	---E--E--	G	----------------------
	Cercocebus atys	XP_011940106	---E--E--	G	----------------------
	Galeopterus variegatus	XP_008587263	---E--G--	G	----------------------
	Trachypithecus francoisi	XP_033093097	--HE--E--	G	----------------------
	Gorilla gorilla gorilla	XP_018890909	---E--E--	G	----R-----------------
	Saimiri boliviensis boliviensis	XP_039334488	---E--E--	G	----------------------
	Propithecus coquereli	XP_012497646	---E--E--	G	----Q-----------------

Supplementary figure S35: Multiple sequence alignment of a conserved segment of the protein cAMP-responsive element modulator, showing a 1 amino acid deletion that is present in the families Muridae and Cricetidae. This CSI was detected in all species within Muridae and Cricetidae, and not detected in any other members of the Euarchontoglires in the top 250 BLASTp returns. The dashes (-) within the alignment indicate the same amino acid as the top line. The second column indicates accession numbers for each of the sequences and the position of the partial sequence is shown by the numbers at the top.

Group	Species	Accession	549		582
Muridae and Cricetidae (15/15)	*Rattus norvegicus*	XP_038951653	KVDNNLIVEKATKKTVV		GKESKPAATKEEPVSTK
	Grammomys surdaster	XP_028645075	-I---------------		-----------------
	Mastomys coucha	XP_031214036	------V----------		-----------------
	Arvicanthis niloticus	XP_034365758	-I---------------		-----------------
	Mus musculus	XP_006516830	------T----------		-----------------
	Mus caroli	XP_029324393	------T----------		---G-------------
	Mus pahari	XP_029403399	------T----------		-----S-----------
	Rattus rattus	XP_032740398	----------T------		-----------------
	*Meriones unguiculatus**	XP_021487057	--------D-V------		-----T-----------
	Mesocricetus auratus	XP_005071888	--------D-T------		-----A-----------
	Cricetulus griseus	XP_027265189	--------D-V------		-----A-----------
	Onychomys torridus	XP_036043784	--------D--------		---G-A-----------
	Peromyscus leucopus	XP_037062354	--------D--------		-----A-----------
	Microtus Ochrogaster	XP_013208587	-G------D--S-----		-----S-----------
	Arvicola amphibius	XP_038190992	--------D--S-----		-----A-----------
**Other *Euarchontoglires***	*Nannospalax galili*	XP_029420837	--E-----D--------	V	-----S-----------
	Jaculus jaculus	XP_012806736	--E-----D--------	V	T----S---------L-
	Castor canadensis	XP_020040393	--------D--I-----	V	-----S--------PL-
	Dipodomys ordii	XP_012888457	--------D--------	V	-----S---------L-
	Fukomys damarensis	XP_010609172	--------D------A-	V	-----S---------L-
	Cavia porcellus	XP_023420914	------VLD-T----A-	V	-----S---------L-
	Chinchilla lanigera	XP_013376609	-------LD------S-	V	-----S---------L-
	Heterocephalus glaber	XP_012925851	--------D------A-	I	-----S---------L-
	Octodon degus	XP_023569134	--E----LD------A-	V	-----S--I------L-
	Marmota marmota marmota	XP_015345576	--------D--------	V	-----S---------L-
	Urocitellus parryii	XP_026243389	--------D--------	V	-----S---------L-
	Marmota monax	VTJ87874	--------D--------	V	-----S---------L-
	Ictidomys tridecemlineatus	XP_013211395	--------D--------	V	-----S---------L-
	Marmota flaviventris	XP_027795424	--------D--------	V	-----S---------L-
	Oryctolagus cuniculus	XP_017199934	--------D--S----I	V	------------L-AV-
	Ochotona princeps	XP_036349610	--------D-TS---AA	V	--D--S------S--L-
	Tupaia chinensis	XP_006172220	--------D-----A--	V	-----S---------L-
	Microcebus murinus	XP_012607728	--------D-----A--	V	---------------L-
	Otolemur garnettii	XP_003797590	--------D-----A--	V	-----S---------L-
	Propithecus coquereli	XP_012509989	--------D-----A--	V	-----S---------L-
	Callithrix jacchus	XP_035109109	--E-----D-----A-I	V	-----S---------L-
	Pongo abelii	XP_024105592	--E-----D-----A-I	V	-----S---------L-
	Aotus nancymaae	XP_012328992	--E-----D-----A-I	V	-----S---------L-
	Nomascus leucogenys	XP_030651531	--E-----D-----A-I	V	-----S---------L-
	Sapajus apella	XP_032100334	--E-----D-----AAI	V	-----S---------L-
	Saimiri boliviensis boliviensi	XP_039318012	--E-----D-----A--	V	-----S--S------L-
	Galeopterus variegatus	XP_008589946	-----SV-D-----A--	V	---------------L-
	Chlorocebus sabaeus	XP_037844119	--E-----D--A--A-I	V	-----S---------L-
	Cercocebus atys	XP_011940309	--E-----D--A--A-I	V	-----S---------L-
	Macaca fascicularis	XP_005549804	--E-----D--A--A-I	V	-----S---------L-
	Rhinopithecus roxellana	XP_010362554	--E-----D--A--A-I	V	-----S---------L-
	Macaca nemestrina	XP_011767382	--E-----D--A--A-I	V	-----S---------L-
	Colobus angolensis palliatus	XP_011799915	--E-----D--A--A-I	V	-----S---------L-
	Rhinopithecus bieti	XP_017722851	--E-----D--A--A-I	V	-----S---------L-
	Homo sapiens	XP_011513899	--E-----D-----A-I	V	-----S------S--L-
	Gorilla gorilla gorilla	XP_030868859	--E-----D-----A-I	V	-----S------S--L-
	Pan paniscus	XP_034819887	--E-----D-----A-I	V	-----S------S--L-
	Hylobates moloch	XP_032614160	--E-----D-----A-I	V	-----S-T-------L-
	Piliocolobus tephrosceles	XP_023082269	--E-----D--A--A-I	V	-----S---------L-
	Trachypithecus francoisi	XP_033049628	--E-----D--A--A-I	V	-----S-S-------L-
	Pan paniscus	XP_034819887	--E-----D-----A-I	V	-----S------S--L-
	Pan troglodytes	XP_001139939	--E-----D-----A-I	V	-----S------S--L-
	Macaca mulatta	XP_014989168	--E-----D--A--A-I	V	-----S---------L-

*Extra S amino acid after position 576

Supplementary figure S36: Multiple sequence alignment of a conserved segment of the protein cyclin-dependent kinase 13, showing a 1 amino acid deletion that is present in the families Muridae and Cricetidae. This CSI was detected in all species within Muridae and Cricetidae, and not detected in any other members of the Euarchontoglires in the top 250 BLASTp returns. The dashes (-) within the alignment indicate the same amino acid as the top line. The second column indicates accession numbers for each of the sequences and the position of the partial sequence is shown by the numbers at the top.

Group	Species	Accession	444		474
**Muridae and Cricetidae (17/17)**	*Mastomys coucha*	XP_031224925	QGDQRTDRSAP		RSASQAEEEPCLEPVKK
	Rattus norvegicus	XP_038951225	-----------		-----------------
	Rattus rattus	XP_032740063	-----------		-----------------
	Arvicanthis niloticus	XP_034365588	-----------		--------------I--
	Grammomys surdaster	XP_028620663	-----------		--------------I--
	Mus musculus	XP_036013681	-----P-----		-----------------
	Mus caroli	XP_021012443	-----P-----		-----------------
	Mus pahari	XP_021049064	-----P-----		-----------------
	Meriones unguiculatus	XP_021484107	--N-----P--		-----------------
	Neotoma lepida	OBS77533	-----------		-----------------
	Onychomys torridus	XP_036043109	-----------		-----------------
	Peromyscus maniculatus bairdii	XP_015863694	-----------		-----------------
	Cricetulus griseus	ERE79348	-----------		-----------------
	Arvicola amphibius	XP_038188664	-----------		-----------------
	Peromyscus leucopus	XP_028726335	-----------		-----------------
	Microtus ochrogaster	XP_005354702	-----A-----		-----------------
	Mesocricetus auratus	XP_021089310	-----------		---------S-------
**Other *Euarchontoglires***	*Nannospalax galili*	XP_008822335	-AE--------	A	--T-----Q--------
	Dipodomys ordii	XP_012870158	-----M-----	A	--T--V----S------
	Castor canadensis	XP_020041119	-----------	A	--T--------------
	Heterocephalus glaber	XP_021096046	------E----	A	-----P-----------
	Chinchilla lanigera	XP_013370890	-----------	A	-----P---------R-
	Octodon degus	XP_004645250	-A---------	A	-----P-----------
	Cavia porcellus	XP_012999992	-----------	A	-----P-----------
	Fukomys damarensis	XP_010606847	---P-------	V	----RP---------R-
	Marmota flaviventris	XP_027784337	-----------	A	-----------------
	Marmota marmota marmota	XP_015338885	-----------	A	-----------------
	Marmota monax	VTJ72831	-----------	A	H----------------
	Ictidomys tridecemlineatus	XP_021582339	-----------	A	-----------------
	Urocitellus parryii	XP_026263522	-----------	A	-----------------
	Oryctolagus cuniculus	XP_017203069	-----------	A	--------------A--
	Ochotona princeps	XP_004578619	--E----H---	A	-------- S-------
	Tupaia chinensis	XP_006159793	-----------	A	-----P----SV-----
	Pan troglodytes	BAK62103	-----------	I	----------SV-----
	Propithecus coquereli	XP_012516397	--E--------	I	-----------------
	Homo sapiens	XP_005252648	-----------	I	----------SV-----
	Microcebus murinus	XP_012604108	--E---E----	I	-----------------
	Aotus nancymaae	XP_012320873	-----------	V	-----P----SM-----
	Carlito syrichta	XP_008053438	-----------	I	-----V----S------
	Saimiri boliviensis boliviensis	XP_003930699	-----------	V	----------SM-----
	Callithrix jacchus	XP_008998251	-----------	I	----------SM-----
	Cercocebus atys	XP_011911340	-----------	I	----------SV-----
	Rhinopithecus bieti	XP_017724708	-----------	I	----------SV-----
	Chlorocebus sabaeus	XP_008000608	-----------	I	----------SV-----
	Trachypithecus francoisi	XP_033065326	-----------	I	----------SV-----
	Piliocolobus tephrosceles	XP_023078908	-----------	I	----------SV-----
	Pongo abelii	XP_024108783	-----------	I	----------SV-----
	Pan paniscus	XP_034785953	-----------	I	----------SV-----
	Gorilla gorilla gorilla	XP_004049162	-----------	I	----------SV-----
	Hylobates moloch	XP_032021745	-----------	I	----------SV-----
	Sapajus apella	XP_032099824	-----------	I	----------GM-----
	Papio anubis	XP_003903483	-----------	I	----------SV-----
	Rhinopithecus roxellana	XP_010362319	-----------	I	----------SV-----
	Macaca fascicularis	XP_005564804	-----------	I	-------D--SV-----
	Macaca mulatta	NP_001244711	-----------	I	-------D--SV-----
	Macaca nemestrina	XP_011750419	-----------	I	-------D--SV-----
	Otolemur garnettii	XP_003786822	--E-K------	V	-----G-----P-----
	Galeopterus variegatus	XP_008568032	-----------	A	-----------P-L---
	Colobus angolensis palliatus	XP_011819259	-----------	I	----------SV-----

Supplementary figure S37: Multiple sequence alignment of a conserved segment of the protein voltage-dependent L-type calcium channel subunit beta-2, showing a 1 amino acid deletion that is present in the families Muridae and Cricetidae. This CSI was detected in all species within Muridae and Cricetidae, and not detected in any other members of the Euarchontoglires in the top 250 BLASTp returns. The dashes (-) within the alignment indicate the same amino acid as the top line. The second column indicates accession numbers for each of the sequences and the position of the partial sequence is shown by the numbers at the top.

Group	Species	Accession	270		297
***Muroidea* (17/17)**	*Mus caroli*	XP_021026397	FPALTSSSSTGSLA		HLGVGGTGQGMNTP
	Mus pahari	XP_029387816	--------------		--------------
	Mus musculus	XP_006509763	--------------		------A-------
	Rattus norvegicus	NP_001040580	-------G------		--------------
	Rattus rattus	XP_032774364	-------G------		------A-------
	Mastomys coucha	XP_031195971	--------------		------A-------
	Meriones unguiculatus	XP_004666068	--V-----------		-----SA-------
	Grammomys surdaster	XP_028641994	--------------		-----SA---L---
	Arvicanthis niloticus	XP_034376210	--------------		-----SA----S--
	Peromyscus leucopus	XP_037067753	--------------		----S-A-------
	Cricetulus griseus	XP_027250490	--------------		----SS--------
	Peromyscus maniculatus bairdii	XP_015862989	--------------		----S-A-------
	Mesocricetus auratus	XP_005086192	-------G------		----S--A------
	Onychomys torridus	XP_036065350	--------------		----SS-----S--
	Microtus ochrogaster	XP_005370610	--------------		---LSS----VS--
	Arvicola amphibius	XP_038183029	--------------		---LSS----VS--
	Nannospalax galili	XP_029415167	----S------N--		---ISS--------
**Other *Rodents* (0/12)**	*Jaculus Jaculus*	XP_004666068	----N--G---N--	TNLT	---IS A---IG--
	Castor canadensis	XP_020032326	----S--G-A-N--	TNLT	---L -A---IS--
	Heterocephalus glaber	XP_012924176	----SG-----N-S	GNLT	---L-------S--
	Chinchilla lanigera	XP_013362761	----SG-----N--	GNLT	---L--A----S--
	Cavia porcellus	XP_023418825	----SG-----N--	GNLT	---L--AS---S--
	Octodon degus	XP_023562973	--T-SG-----N--	GNLT	---L--A---VS—
	Fukomys damarensis	XP_033615289	----SG-----N-S	GNLT	---L--A----S--
	Urocitellus parryii	XP_026245749	----S------N--	TNLT	---I-------S--
	Ictidomys tridecemlineatus	XP_021587174	----S------N--	ANLT	---I--A----S--
	Marmota flaviventris	XP_027787862	----S------N--	ANLT	---I--A----S--
	Marmota monax	VTJ54693	----S------N--	ANLT	---I--A----S--
	Marmota marmota marmota	XP_015344391	----S------N--	ANLT	---I--A----S--
**Other *Euarchontoglires***	*Tupaia chinensis*	ELW67140	----S------N--	ANLT	---I------VS--
	Macaca mulatta	XP_028695434	----S------N--	ANLT	---I----------
	Nomascus leucogenys	XP_030676240	----S------N--	ANLT	---I--A-------
	Cercocebus atys	XP_011949868	----S------N--	ANLT	---I-------S--
	Trachypithecus francoisi	XP_033055947	----S------N--	ANLT	---I-------S--
	Macaca nemestrina	XP_011746244	----S------N--	ANLT	---I-------S--
	Papio anubis	XP_031514907	----S------N--	ANLT	---I-------S--
	Chlorocebus sabaeus	XP_007994025	----S------N--	ANLT	---I-------S--
	Macaca fascicularis	XP_015295703	----S------N--	ANLT	---I-------S--
	Colobus angolensis palliatus	XP_011781308	----S------N--	ANLT	---I-------S--
	Rhinopithecus bieti	XP_017708153	----S------N--	ANLT	---I-------S--
	Microcebus murinus	XP_020139555	----S--G---N--	ATLT	---I--A----S--
	Homo sapiens	XP_024307202	----S------N--	ANLT	---I--A----S--
	Pongo abelii	XP_024092425	----S------N--	ANLT	---I--A----S--
	Sapajus apella	XP_032123787	----S------N--	ANLT	---I--A----S--
	Pan troglodytes	PNI50399	----S------N--	ANLT	---I--A----S--
	Hylobates moloch	XP_032026067	----S------N--	ANLT	---I--A----S--
	Pan paniscus	XP_008969268	----S------N--	ANLT	---I--A----S--
	Propithecus coquereli	XP_012509563	----S--G---N--	ATLT	---I-------S--
	Callithrix jacchus	XP_035142232	--S-SG-----N--	ANLT	---I--A----S--
	Theropithecus gelada	XP_025223721	----S------N--	ANLT	---I-------S--
	Rhinopithecus roxellana	XP_030791441	----S------N--	ANLT	---I-------S--
	Otolemur garnettii	XP_003796627	----S------N--	ANLT	---I--A----S--
	Saimiri boliviensis boliviensis	XP_003941204	----S------N--	ANLT	---T--A----S--
	Carlito syrichta	XP_021563889	----S------N--	ANLT	---I- -----S--
	Gorilla gorilla gorilla	XP_030860292	----S------N--	ANLT	---I--A---

Supplementary figure S38: Multiple sequence alignment of a conserved segment of the protein CREB-regulated transcription coactivator 1, showing a 4 amino acid deletion that is present in the superfamily Muroidea. This CSI was detected in all species within Muroidea, and not detected in any other members of the Euarchontoglires in the top 250 BLASTp returns. The dashes (-) within the alignment indicate the same amino acid as the top line. The second column indicates accession numbers for each of the sequences and the position of the partial sequence is shown by the numbers at the top.

Group	Species	Accession	86		119
**Muroidea (17/17)**	*Rattus norvegicus*	149065159	EFTTNRKCFEEDFKTQVQD	T	KEWLELEEDAQKTY
	Mus musculus	148681817	-------------R-----	-	--------------
	Mus pahari	XP_029391123	-------------------	-	--------------
	Meriones unguiculatus	XP_021496972	-----K-------------	-	--------------
	Grammomys surdaster	XP_028618448	-------------------	-	--------------
	Arvicanthis niloticus	XP_034374645	-------------------	-	--------------
	Mastomys coucha	XP_031237256	-----K-------------	-	--------------
	Mus caroli	XP_021020975	-------------R-----	-	--------------
	Rattus rattus	XP_032762712	-------------------	-	--------------
	Peromyscus maniculatus bairdii	589933354	------R------------	-	--------------
	Cricetulus griseus	354470741	------R------R-----	-	--------------
	Mesocricetus auratus	524956403	------R-------M----	-	--------------
	Microtus ochrogaster	532046047	------R------R-----	A	--------------
	Onychomys torridus	XP_036038231	------R------------	-	--------------
	Peromyscus leucopus	XP_028722021	------R------------	-	--------------
	Arvicola amphibius	XP_038175472	---A--R------RA----	A	--------------
	Nannospalax galili	674074575	--S---R------------	A	N-------------
**Other *Euarchontoglires***	*Castor canadensis*	XP_020015567	------R-----------G		--------------
	Dipodomys ordii	XP_012875505	-----KR-----------G		--------------
	Fukomys damarensis	731236434	---A--R-----------G		--------------
	Cavia porcellus	348578933	---A--R-----------S		--------------
	Octodon degus	507693936	---S--R-----------G		--------------
	Chinchilla lanigera	533178444	---A--R-------I---G		--------------
	Heterocephalus glaber	512988993	---A--R-----------G		------------A-
	Ictidomys tridecemlineatus	532090959	------R-----------G		--------------
	Marmota flaviventris	XP_027805951	------R-----------G		--------------
	Urocitellus parryii	XP_026248585	------R-----------G		--------------
	Marmota marmota marmota	XP_015349395	------R-----------G		--------------
	Ochotona princeps	504165129	------R-----------N		------------A-
	Oryctolagus cuniculus	655818470	------R-----------N		------------A-
	Tupaia chinensis	444706709	------R-----------G		------------A-
	Microcebus murinus	829888671	---N--R-----------G		--------------
	Propithecus coquereli	826351699	---N--R-----------G		--------------
	Homo sapiens	767948354	---N--R-----------G		------------A-
	Aotus nancymaae	817317694	---N--R-----------G		------------A-
	Callithrix jacchus	390467167	---N--R-----------G		------------A-
	Saimiri boliviensis boliviensi	403256838	---N--R-----------G		------------A-
	Macaca nemestrina	795574889	---N--R-----------G		------------A-
	Pan troglodytes	694924087	---N--R-----------G		------------A-
	Chlorocebus sabaeus	635116205	---N--R-----------G		------------A-
	Papio anubis	402864798	---N--R-----------G		------------A-
	Macaca mulatta	355560984	---N--R-----------G		------------A-
	Colobus angolensis palliatus	795153015	---N--R-----------G		------------A-
	Gorilla gorilla gorilla	426357869	---N--R-----------G		------------A-
	Pan paniscus	397484761	---N--R-----------G		------------A-
	Cercocebus atys	795402992	---N--R-----------G		------------A-
	Rhinopithecus roxellana	724899559	---N--R-----------G		------------A-
	Macaca fascicularis	544422760	---N--R-----------G		------------A-
	Pongo abelii	395738956	---N--R-----------G		------------A-
	Rhinopithecus bieti	XP_017751698	---N--R-----------G		------------A-
	Carlito syrichta	XP_008069767	---N--R-----------G		------------A-
	Trachypithecus francoisi	XP_033048894	---N--R-----------G		------------A-
	Nomascus leucogenys	XP_030681402	---N--R-----------G		------------A-
	Theropithecus gelada	XP_025235323	---N--R-------S---G		------------A-
	Sapajus apella	XP_032131896	---N--R-----------G		------G-----A-
	Otolemur garnettii	XP_003789788	--AN--R-------M---G		------------A-

Supplementary figure S39: Multiple sequence alignment of a conserved segment of the protein striatin-interacting proteins 2, showing a 1 amino acid insertion that is present in the superfamily Muroidea. This CSI was detected in all species within Muroidea, and not detected in any other members of the Euarchontoglires in the top 250 BLASTp returns. The dashes (-) within the alignment indicate the same amino acid as the top line. The second column indicates accession numbers for each of the sequences and the position of the partial sequence is shown by the numbers at the top.

Group	Species	Accession	953		988
**_Muridae (7/9)_**	*Rattus norvegicus*	XP_038951682	ASGRDLGQIEDNDQARK	VRTE	FLFLSEVLQWRAQTTPDHLL
	Mus musculus	BAC29340	-----------------	----	--------------------
	Rattus rattus	XP_032740394	-----------------	----	--------------------
	Arvicanthis niloticus	XP_034366827	-----------------	----	--------------------
	Mastomys coucha	XP_031215443	-----------------	----	--------------------
	Mus caroli	XP_029324423	-----------------	----	--------------------
	Mus pahari	XP_029403560	-----------------	----	--------------------
	Meriones unguiculatus	XP_021497712	-----------------		--------------------
	Grammomys surdaster	XP_028625693	-----------------		--------------------
**_Cricetidae (8/8)_**	*Onychomys torridus*	XP_036044763	-----------------	----	--------------------
	Peromyscus leucopus	XP_028715289	-----------------	----	--------------------
	Microtus ochrogaster	XP_026644216	-----------------	----	--------------------
	Arvicola amphibius	XP_038190432	-----------------	----	--------------------
	Cricetulus griseus	XP_027260866	-----------------	----	--------------------
	Mesocricetus auratus	XP_012970124	-----------------	----	--------------------
	Peromyscus maniculatus bairdii	XP_015854224	-----------------	----	--------------------
**_Spalacidae_**	*Nannospalax galili*	XP_029423345	-----------------	----	------------------V-
**Other _Euarchontoglires_ _(1/40)_**	*Jaculus jaculus*	XP_004662224	-----------------		------------------V-
	Castor canadensis	XP_020016509	-----------------		------------------V-
	Dipodomys ordii	XP_012864679	-----------------		------------------V-
	Fukomys damarensis	KFO18507	-----------------		------------------V-
	Octodon degus	XP_023557096	-----------------		------------------V-
	Heterocephalus glaber	XP_021122018	-----------------		------------------V-
	Chinchilla lanigera	XP_013360167	-----------------		------------------V-
	Cavia porcellus	XP_023422285	-----------------		------------------V-
	Ictidomys tridecemlineatus	XP_021578740	-----------------		------------------V-
	Marmota marmota marmota	XP_015350748	-----------------		------------------V-
	Urocitellus parryii	XP_026268187	-----------------		------------------V-
	Marmota flaviventris	XP_027803831	-----------------		------------------V-
	Marmota monax	KAF7466056	-----------------		------------------V-
	Tupaia chinensis	ELW71662	-----------------		------------------V-
	Carlito syrichta	XP_021563661	-----------------	-G-Q	------------------V-
	Homo sapiens	BAG60597	-----------------		------------------I-
	Colobus angolensis palliatus	XP_011797692	-----------------		------------------V-
	Sus scrofa	XP_020920735	-----------------		------------------V-
	Cercocebus atys	XP_011948560	-----------------		------------------V-
	Saimiri boliviensis boliviensis	XP_010348013	-----------------		------------------V-
	Aotus nancymaae	XP_012322382	-----------------		------------------V-
	Otolemur garnettii	XP_012658806	-----------------		------------------V-
	Sapajus apella	XP_032122040	-----------------		------------------V-
	Nomascus leucogenys	XP_030675234	-----------------		------------------V-
	Propithecus coquereli	XP_012520396	-----------------		------------------V-
	Rhinopithecus roxellana	XP_030795849	-----------------		------------------V-
	Papio anubis	XP_031508430	-----------------		------------------V-
	Macaca nemestrina	XP_011755041	-----------------		------------------V-
	Pan troglodytes	PNI17473	-----------------		------------------V-
	Pongo abelii	PNJ08438	-----------------		------------------V-
	Macaca fascicularis	XP_005564543	-----------------		------------------V-
	Macaca mulatta	EHH18846	-----------------		------------------V-
	Callithrix jacchus	XP_035108629	-----------------		------------------V-
	Chlorocebus sabaeus	XP_037840912	----E-AHL--S-----		----AD------H-----P-
	Pan paniscus	XP_034803923	----E-AHL--S-----		----AD------H-----P-
	Theropithecus gelada	XP_025235911	----E-AHL--S-----		----AD------H-----P-
	Trachypithecus francoisi	XP_033088591	----E-AHL--S-----		----AD------H-----P-
	Rhinopithecus bieti	XP_017717658	-----------------		------------------V-
	Gorilla gorilla gorilla	XP_018873306	----E-AHL--S-----		----AD------H-----P-
	Hylobates moloch	XP_032030437	----E-AHL--S-----		----AD------H-----P-

Supplementary figure S40: Multiple sequence alignment of a conserved segment of the protein disco-interacting protein 2 homolog C, showing a 4 amino acid insertion that is present in the superfamily Muroidea. This CSI was detected in all species within Muroidea, except for 2, and only detected in 1 other member of the Euarchontoglires in the top 250 BLASTp returns. The dashes (-) within the alignment indicate the same amino acid as the top line. The second column indicates accession numbers for each of the sequences and the position of the partial sequence is shown by the numbers at the top.

Group	Species	Accession	2511		2535
Muroidea (18/18)	*Rattus rattus*	XP_032740137	GLQVL		ANAPAQSSPAPQAHIPGLQI
	Meriones unguiculatus	XP_021485995	-----		--------------------
	Mastomys coucha	XP_031215223	-----		--S-----------------
	Arvicanthis niloticus	XP_034365949	-----		--T----N------------
	Grammomys surdaster	XP_028618963	-----		--T----N------------
	Rattus norvegicus	XP_038951236	-----		----------T-T-------
	Mus pahari	XP_021071005	-----		----T------P--------
	Mus musculus	XP_006516902	-----		-----------P---Q----
	Mus caroli	XP_029324455	-----		-----------P---Q----
	Onychomys torridus	XP_036042879	-----		--------------------
	Peromyscus leucopus	XP_028723588	-----		--------------------
	Arvicola amphibius	XP_038189002	-----		--------------------
	Neotoma lepida	OBS59733	-----		--------------------
	Mesocricetus auratus	XP_005066417	-----		--------------------
	Microtus ochrogaster	XP_005355142	-----		-----------K--------
	Peromyscus maniculatus bairdii	XP_015846493	-----		------------V-------
	Cricetulus griseus	XP_035299812	-----		---S----------------
	Nannospalax galili	XP_029418688	-----		-R------------L-----
**Other *Euarchontoglires***	*Castor canadensis*	XP_020008398	-----	T	--P-S--N-PA---------
	Dipodomys ordii	XP_012891958	-----	T	--P-P------P--------
	Octodon degus	XP_023567846	-----	T	-SPSS----T----------
	Chinchilla lanigera	XP_013377305	-----	T	-SPSS----T----------
	Heterocephalus glaber	XP_004847740	-----	T	-SPSS----T----------
	Fukomys damarensis	XP_010621169	-----	A	-SPSS----T----------
	Cavia porcellus	XP_003468890	----V	A	-SP-SR--------------
	Marmota flaviventris	XP_027804013	-----	A	GTPSS---------L-----
	Marmota marmota marmota	XP_015349502	-----	A	GTPSS---------L-----
	Ictidomys tridecemlineatus	XP_013215113	-----	A	GTPSS---------L-----
	Marmota monax	VTJ53383	-----	A	GTPSS---------L-----
	Urocitellus parryii	XP_026240382	-----	A	GSPSS---------L-----
	Tupaia chinensis	XP_006141245	-----	A	-SP------G--GP------
	Propithecus coquereli	XP_012508610	-----	T	--PSS---------------
	Otolemur garnettii	XP_012659708	-----	T	--PAS---------------
	Callithrix jacchus	XP_002746269	-----	T	--PSP---------------
	Saimiri boliviensis boliviensi	XP_010336349	-----	T	--PSP---------------
	Sapajus apella	XP_032103601	-----	T	--PSP---------------
	Aotus nancymaae	XP_012331954	-----	T	--PSP---------------
	Pan paniscus	XP_034817333	-----	T	--PSS----V----------
	Rhinopithecus bieti	XP_017746654	-----	T	--PSS---------------
	Homo sapiens	P15822	-----	T	--PSS---------------
	Hylobates moloch	XP_031996134	-----	T	--PSS---------------
	Carlito syrichta	XP_008050455	-----	T	--PSS---------------
	Gorilla gorilla gorilla	XP_030868274	-----	T	--PSS---------------
	Rhinopithecus roxellana	XP_030784718	-----	T	--PSS---------------
	Pongo abelii	PNJ71153	-----	T	--PSS---------------
	Nomascus leucogenys	XP_030673156	-----	T	--PSS---------------
	Chlorocebus sabaeus	XP_007971868	-----	T	--PSS---------------
	Macaca nemestrina	XP_011740828	-----	T	--PSS---------------
	Colobus angolensis palliatus	XP_011818085	-----	T	--PSS---------------
	Theropithecus gelada	XP_025239942	-----	T	--PSS---------------
	Pan troglodytes	XP_009448792	-----	T	--PSS---------------
	Trachypithecus francoisi	XP_033072121	-----	T	--PSS---------------
	Papio anubis	XP_031522773	-----	T	--PSS---------------
	Cercocebus atys	XP_011886690	-----	T	--PSS---------------
	Macaca fascicularis	XP_005554085	-----	T	--PSS---------------
	Macaca mulatta	XP_002803653	-----	T	--PSS---------------

Supplementary figure S41: Multiple sequence alignment of a conserved segment of the protein zinc finger protein 40, showing a 1 amino acid deletion that is present in the superfamily Muroidea. This CSI was detected in all species within Muroidea, and not detected in any other members of the Euarchontoglires in the top 250 BLASTp returns. The dashes (-) within the alignment indicate the same amino acid as the top line. The second column indicates accession numbers for each of the sequences and the position of the partial sequence is shown by the numbers at the top.
